# Brain Morphological Characteristics of Cognitive Subgroups of Schizophrenia-Spectrum Disorders and Bipolar Disorder: A Systematic Review with Narrative Synthesis

**DOI:** 10.1007/s11065-021-09533-0

**Published:** 2022-02-22

**Authors:** James A. Karantonis, Sean P. Carruthers, Katherine E. Burdick, Christos Pantelis, Melissa Green, Susan L. Rossell, Matthew E. Hughes, Vanessa Cropley, Tamsyn E. Van Rheenen

**Affiliations:** 1 Melbourne Neuropsychiatry Centre, Level 3, Alan Gilbert Building, 161 Barry St, Carlton, VIC 3053 Australia; 2 Faculty of Health, Arts and Design, School of Health Sciences, Centre for Mental Health, Swinburne University, Melbourne, Australia; 3 St Vincent’s Mental Health, St Vincent’s Hospital, Fitzroy, VIC Australia; 4 Florey Institute of Neuroscience and Mental Health, Parkville, Australia; 5 Department of Electrical and Electronic Engineering, University of Melbourne, VIC, Australia; 6 Brigham and Women’s Hospital, Boston, MA USA; 7 Department of Psychiatry, Harvard Medical School, Boston, MA USA; 8 School of Psychiatry, University of New South Wales (UNSW), Sydney, NSW Australia; 9 Neuroscience Research Australia, Randwick, NSW Australia

**Keywords:** Cognition, Morphology, Bipolar disorder, Schizophrenia, Cognitive subgroups, Heterogeneity

## Abstract

Despite a growing body of research, there is yet to be a cohesive synthesis of studies examining differences in brain morphology according to patterns of cognitive function among both schizophrenia-spectrum disorder (SSD) and bipolar disorder (BD) individuals. We aimed to provide a systematic overview of the morphological differences—inclusive of grey and white matter volume, cortical thickness, and cortical surface area—between cognitive subgroups of these disorders and healthy controls, and between cognitive subgroups themselves. An initial search of PubMed and Scopus databases resulted in 1486 articles of which 20 met inclusion criteria and were reviewed in detail. The findings of this review do not provide strong evidence that cognitive subgroups of SSD or BD map to unique patterns of brain morphology. There is preliminary evidence to suggest that reductions in cortical thickness may be more strongly associated with cognitive impairment, whilst volumetric deficits may be largely tied to the presence of disease.

**Supplementary Information:**

The online version contains supplementary material available at 10.1007/s11065-021-09533-0.

## Introduction

Cognitive impairment appears to be a feature for many individuals with a schizophrenia-spectrum disorders (SSD) or bipolar disorder (BD), with the domains of executive functioning, attention, processing speed and memory most typically affected (Antonova et al., 2004; Bora et al., 2009; Burdick et al., 2011; Harvey & Rosenthal, 2018; Sperry et al., 2015; Van Rheenen & Rossell, 2014). Over recent years, there has been a surge in research on cognitive heterogeneity within these disorders, where the presence of two-to-four cognitive subgroups has been observed across a multitude of studies in SSD and BD independently. Despite the considerable methodological variation between them, there is evidence of an anchoring by one subgroup with either severe or global cognitive impairments, and another with relatively intact cognition (Ammari et al., 2010; Bora, 2016; Carruthers et al., 2019a, b; Gilbert et al., 2014; Green et al., 2012; Lewandowski et al., 2014; Reser et al., 2015; Shepherd et al., 2015; Van Rheenen et al., 2017; Woodward & Heckers, 2015). This cognitive cluster structure has also been demonstrated when SSD and BD samples are analyzed cross-diagnostically (Karantonis et al., 2020; Lee et al., 2017; Lewandowski et al., 2018; Van Rheenen et al., 2017).

Variation in objectively measured cognitive functioning may reflect variation in underlying neuropathology that likely cuts across diagnostic boundaries. As such, a number of recent studies have focused on characterizing the brain morphological correlates of cognitive subgroups, either in SSD or BD independently, or across both disorders cross-diagnostically. Findings pertaining to between-subgroup differences in brain morphology are of value, as they have implications for differences in illness trajectory and aetiology. However, the findings across studies vary (Shepherd et al., 2015; Wexler et al., 2009), and to date there has not been a systematic synthesis of these studies that provides insight into the extent of overlap or differences between them. Further, given that there are subgroups of patients who are cognitively similar to healthy controls, there is no unified understanding of whether the brain morphology of these patients deviates from the norm.

In this review, we aim to provide a foundation from which the findings of the available studies can be drawn together in a comprehensive synthesis of existing literature investigating structural brain morphology (including grey and white matter volume, cortical thickness and cortical surface area) in relation to cognitive subgroups in SSD or BD. Given overlapping cognitive patterns in these disorders (Carruthers et al., 2019b; Harvey et al., 2010; Karantonis et al., 2020, 2021; Lee et al., 2017; Shepherd et al., 2015; Van Rheenen et al., 2017, 2020; Woodward & Heckers, 2015), it is hoped that these diagnostic-based comparisons will further elucidate the nature and extent of their proximity on the psychosis-mood spectrum. Specifically, our objective is to clarify if and how brain morphology differs between cognitive subgroups identified in these disorders, and in relation to healthy controls. In doing so, we aim to answer three research questions; are there specific morphological abnormalities that are: 1) more heavily associated with cognitive impairment independent of SSD or BD presence; 2) uniquely associated with SSD or BD presence independent of cognitive impairment; and 3) associated with the interaction of cognitive impairment and disease presence, and that may indicate the extent to which the cognitive subgroups are either neurobiologically distinct or rather exist on a continuum. Given that our initial scope of the literature indicated a relative absence of longitudinal cognitive subgroup studies, in this review we also examine the nature of relationships evident in recent-onset individuals compared to those with an established illness in order to better understand the dynamics of brain-cognition relationships across the illness course.

**Fig. 1:**
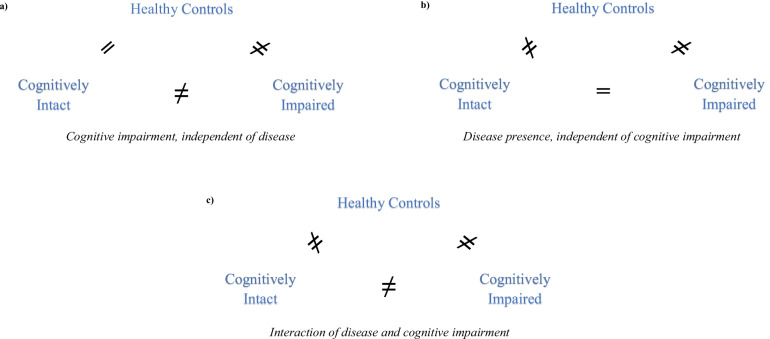
Three models to aid with data interpretation. Visual models of patterns of findings, **a**) findings likely to indicate brain morphological abnormalities more strongly associated with cognitive impairment, independent of SSD and/or BD disease presence; **b**) findings likely to indicate brain morphological abnormalities associated with SSD and/or BD disease presence, independent of cognitive impairment; **c**) findings likely to indicate brain morphological abnormalities reflecting the interaction of SSD and/or BD disease presence and cognitive impairment. Note ‘≠’ is indicative that there is a significant difference in brain morphology between (sub)groups; ‘=’ is indicative that there is no significant difference in brain morphology between (sub)groups. For brevity, the term ‘intact’ is used synonymously with ‘relatively intact’

We reason that a pattern of data indicating an absence of morphological differences between the relatively intact cognitive subgroup and healthy controls, in the presence of differences in the same regions between the cognitively impaired subgroup and both of these groups, will provide evidence to answer question 1 (Fig. 1a). In contrast, data indicating specific differences between cognitive subgroups and healthy controls in the absence of differences in the same regions between cognitive subgroups themselves, will provide evidence to address question 2 (Fig. 1b). Further, data indicating specific morphological abnormalities in cognitively impaired and relatively intact subgroups compared to healthy controls *and also* between the cognitive subgroups themselves, will provide evidence to address question 3 (Fig. 1c). Collectively, the findings of the review are expected to shed light on the extent to which brain morphology maps to cognitive subgroups on the SSD-BD spectrum.

## Method

### Search Strategy

This review was conducted in accordance with the Preferred Reporting Items for Systematic Reviews and Meta-Analyses (PRISMA) statement (Moher et al., 2009), when applicable. The literature was searched for studies published in the online databases of PubMed and Scopus after January 1^st^ 1990 and before 1^st^ of June 2020. The search syntax we employed was based on three concepts: diagnosis, cognition, and brain morphology and optimized for each database. Record type was limited to full-length, published empirical articles. The final search syntax optimized for each database is provided in the supplementary material.

### Eligibility Criteria

Study eligibility was assessed at two stages of screening. In stage one, titles, abstracts, and keywords were scanned by one author and deemed eligible if they met the following criteria: i) written in English and empirical in format; ii) included participants with SSD or BD; and iii) examined brain morphology and cognition. SSD were defined as disorders inclusive of schizophrenia, schizoaffective disorder, and schizophreniform disorder; BD were defined as subtypes inclusive of type I, II, and BD with or without psychotic features. Brain morphology was inclusive of grey and white volume, cortical thickness, and cortical surface area, with images recorded by structural imaging. In stage two, two reviewers independently screened full-text articles. A study was eligible if: i) the patient sample was categorized into subgroups using cognitive performance alone, ii) the brain morphology of these subgroups was examined using magnetic resonance imaging (MRI) (as opposed to the examination of correlations between brain morphology and cognitive performance within the subgroups); iii) it included participant samples with a mean age under 65 years of age; iv) non-social cognitive performance was examined. In addition, a paper was only eligible if it included an independent sample (i.e. papers with identical samples were excluded). However, in the instance that a sample had been analyzed more than once, the article was included if it involved a different brain measure (i.e. two papers with the same sample were accepted if one examined volume whilst the other examined cortical thickness). If a sample was published with overlapping brain measures across papers, only the most comprehensive analysis was included in the review, if eligible (i.e. if one paper examined volume, and the other examined volume and thickness, the second paper was included and the first excluded).

### Data Extraction

The following information was extracted from all papers: participant demographics including age and sex, MRI scanner type, brain atlases used, brain regions examined, cognitive domains and cognitive measures examined, statistical analyses used, number of subgroups identified, how subgroups were categorized, significant differences in brain morphology between cognitive subgroups and relative to healthy controls. A summary of this information is provided in Table 1 and Table 2. It is recommended that Table 1 is consulted when reading through the results section of this review for more detail on how subgroups were formed, their characteristics, their sample sizes, and between-group differences. Similarly, Table 2 can be used for an overview of the neuroimaging related parameters and a brief summary of findings between subgroups and between subgroups and healthy controls.

**Table 1:** Summary of cognitive subgroup classification

**Authors**	**Cognitive domains used to classify subgroups**	**Subgrouping Approach**	**Cognitive Subgroups**	**Cohort, Subgroup #,** **Mean Age ± SD (*n*)**	**Clinical differences between clusters**
(Alonso-Lana et al., 2016)	Executive Function, Verbal Memory/Learning, Visual Memory/Learning	Cut-off scores	1) Impaired: ≤ *7 RBMT,* ≤ *11 BADS* 2) Preserved: ≥ *8 RBMT,* ≥ *12 BADS*	BD 1): 46.17 ± 7.40 (28) BD 2): 44.13 ± 6.63 (33) HC: 44.01 ± 6.03 (28)	None
(Ayesa-Arriola et al., 2013)*	Premorbid IQ, Attention, Executive Function, Speed of Processing, Verbal Memory/Learning, Visual Memory/Learning, Working Memory	Cut-off scores	1) Non-deficit: *above average general cognitive functioning* 2) Deficit: *below average general cognitive functioning*	RO-SSD 1): 28.50 ± 6.93 (29) RO-SSD 2): 32.12 ± 9.97 (30) HC: N/A	Positive symptoms
(Cobia et al., 2011)	Episodic Memory, Executive Function, Verbal IQ, Working Memory	Data-driven	1) Neuropsychologically Impaired: *mild-moderately impaired mean neuropsychological scores* 2) Neuropsychologically Near normal: *average-low mean neuropsychological scores*	SSD 1): 36.57 ± 3.85 (34) SSD 2): 32.38 ± 13.37 (45) HC: 34.15 ± 12.75 (65)	Ethnicity, Parental SES
(Colibazzi et al., 2013)	Working Memory	Cut-off scores	1) Neuropsychologically impaired: *1 SD below HC mean* 2) Neuropsychologically near-normal: < *0.5 SD of HC mean*	SSD 1): N/A (50) SSD 2: N/A (21) HC: 38.36 ± 11.85 (57)	None
(Czepielewski et al., 2017)	Premorbid IQ, Verbal Memory/Learning, Visual Memory/Learning, Working Memory	Cut-off scores	1) IQ + /COG + : *average premorbid IQ and current cognition* 2) IQ + /COG − : *average premorbid IQ and impaired current cognition* 3) IQ − /COG − : *impaired premorbid IQ and current cognition*	SSD 1): 32.20 ± 12.39 (25) SSD 2): 32.22 ± 13.64 (31) SSD 3): 38.44 ± 12.67 (36) HC: 32.22 ± 13.64 (94)	Education, Negative symptoms, SES
(Geisler et al., 2015)	Executive Function, Speed of Processing, Verbal Fluency, Verbal Knowledge/Abstraction, Verbal Memory/Learning, Visual Memory/Learning, Working Memory	Data-driven	1) Diminished verbal fluency 2) Diminished verbal and poor motor control 3) Diminished face memory and slowed processing 4) Diminished intellectual function	SSD 1): 32.10 ± 10.40 (38) SSD 2): 26.80 ± 10.70 (26) SSD 3): 37.60 ± 13.20 (21) SSD 4): 29.80 ± 9.90 (44) HC: 31.40 ± 11.10 (165)	Duration of illness, Education, Positive symptoms, Premorbid IQ
(Gould et al., 2014)	Attention, Current IQ, Language, Memory, Premorbid IQ, Verbal Memory/Learning, Working Memory	Data-driven	1) Cognitively impaired: *based on current and premorbid IQ* 2) Cognitively preserved: *based on current and premorbid IQ*	SSD 1: N/A (74) SSD 2: N/A (126) HC: N/A (178)	Current IQ, Education, Negative symptoms, Premorbid IQ
(Guimond et al., 2016)	Verbal Memory/Learning	Cut-off scores	1) Low to mild: *ISLT z-score* ≤ *-1.4* 2) Moderate to severe: *ISLT z-score* > *-1.4*	SSD 1): 34.57 ± 8.70 (23) SSD 2): 35.85 ± 8.66 (27) HC: 33.26 ± 8.17 (23)	Current IQ, Negative symptoms
(Ho et al., 2020)*	Current IQ, premorbid IQ	Cut-off scores	1) Preserved IQ: *average premorbid IQ and current cognition* 2) Deteriorated IQ: *average premorbid IQ and impaired current cognition* 3) Compromised IQ: *impaired premorbid IQ and impaired cognition*	SSD/BD 1) 31.66 ± 8.20 (54) SSD/BD 2) 30.93 ± 8.14 (111) SSD/BD 3) 39.98 ± 10.78 (6) HC: 32.82 ± 9.73 (633)	Age, Current IQ, Education, Premorbid IQ, Age of onset
(Ortiz-Gil et al., 2011)	Executive Function, Verbal Memory/Learning, Visual Memory/Learning	Cut-off scores	1) Impaired: < *7 RBMT,* < *8 BADS* 2) Preserved: ≥ *8 RBMT,* ≥ *12 BADS*	SSD 1): 42.38 ± 8.23 (26) SSD 2): 40.10 ± 10.22 (23) HC: 40.10 ± 11.58 (39)	Current IQ, Duration of illness
(Poletti et al., 2014)	Working Memory	Cut-off scores	1) Bad performers: < *25% correct responses on N-back* 2) Good performers: > *25% correct responses on N-back*	SSD 1): 36.59 ± 8.13 (22) SSD 2): 34.66 ± 9.45 (28) HC: N/A	Current IQ
(Rusch et al., 2007)	Executive Function	Cut-off scores	1) Poor WCST performers: < *2 categories* 2) High WCST performers: *5 or 6 categories*	SSD 1): 38.8 ± 10.50 (21) SSD 2): 35.90 ± 10.70 (30) HC: 37.10 ± 10.50 (62)	Education
(Shepherd et al., 2015)	Working Memory	Cut-off scores	1) Executively deficit: < *50% accuracy on the N-back* 2) Executively spared: > *50% accuracy on the N-back*	SSD 1): 44.90 ± 11.80 (22) SSD 2): 38.20 ± 10.80 (18) BD 1): 44.90 ± 11.80 (10) BD 2): 38.20 ± 10.80 (20) HC: 32.6 ± 10.60 (34)	Age, Functioning, Negative symptoms
(Torres et al., 1997)	Verbal Memory/Learning, Visual Memory/Learning	Cut-off scores	1) Low memory: *top 10 performers on the WMS* 2) High memory: *bottom 10 performers on the WMS*	SSD 1): 26.90 ± 5.20 (10) SSD 2): 24.30 ± 7.70 (10) HC: N/A	Current IQ
(Van Rheenen et al., 2018)	Premorbid IQ, Attention, Verbal Memory/Learning, Visual Memory/Learning, Working Memory	Data-driven	1) Preserved IQ: *average premorbid IQ and current cognition* 2) Deteriorated IQ: *average premorbid IQ and impaired current cognition* 3) Compromised IQ: *impaired premorbid IQ and impaired cognition*	SSD 1): 37.80 ± 8.45 (73) SSD 2): 36.05 ± 9.33 (100) SSD 3): 46.06 ± 10.97 (47) HC: 39.74 ± 13.74 (168)	Duration of illness, Functioning, Medication, Negative symptoms, Sex
(Vaskinn et al., 2015)	Full Scale IQ	Cut-off scores	1) Intellectually normal: *100* ≤ *IQ* ≤ *115* 2) Intellectually superior: *IQ* ≥ *120* 3) Intellectually low: *80* ≤ *IQ* ≤ *95*	SSD 1): 29.80 ± 7.90 (41) SSD 2): 32.40 ± 5.80 (12) SSD 3) 34.50 ± 9.70 (16) HC: N/A (86)	Education
(Weinberg et al., 2016)	Full Scale IQ, Premorbid IQ	Data-driven	1) Putatively Preserved: *average premorbid IQ and current cognition* 2) Moderately Deteriorated: *average premorbid IQ and moderately impaired current cognition* 3) Severely Deteriorated: *average premorbid IQ and severely impaired current cognition* 4) Compromised: *average premorbid IQ and impaired current cognition*	SSD 1): 35.50 ± 10.10 (21) SSD 2): 37.20 ± 7.90 (19) SSD 3): 35.40 ± 7.70 (14) SSD 4): 32.20 ± 7.30 (5) HC: 31.90 ± 8.40 (87)	Education, Negative symptoms, Premorbid IQ, Total symptoms
(Wexler et al., 2009)	Working Memory	Cut-off scores	1) Neuropsychologically Impaired: > *1 SD of HC mean* 2) Neuropsychologically Near Normal: < *0.5 SD of HC mean*	SSD 1): 42.60 ± 9.60 (32) SSD 2): 39.50 ± 9.10 (14) HC: 7.50 ± 11.00 (30)	Education, Medication
(Woodward & Heckers, 2015)	Global Cognition, Premorbid IQ,	Cut-off scores	1) Neuropsychologically normal: *premorbid IQ* > *10*^*th*^* % of HC and* < *0.8 SD difference between actual and predicted SCIP score (after regressing out age, sex and premorbid IQ predicted level)* 2) Neuropsychologically impaired: *premorbid IQ* < *10*^*th*^* % of HC or* > *0.8 SD difference between actual and predicted SCIP score (after regressing out age, sex and premorbid IQ)* 2a) Compromised: *premorbid IQ* < *10*^*th*^* % of HC* 2b) Deteriorated: *premorbid IQ* > *10*^*th*^* % of HC*	SSD 1): 28.90 ± 10.30 (28) SSD 2): 34.60 ± 12.90 (73) BD 1): 28.90 ± 10.30 (13) BD 2): 34.60 ± 12.90 (17) HC: 33.3 ± 11.5 (56)	Age, Premorbid IQ
(Yasuda et al., 2020)	Full Scale IQ, Premorbid IQ	Cut-off scores	1) Preserved IQ: *average premorbid IQ and current cognition* 2) Deteriorated IQ: *average premorbid IQ and impaired current cognition* 3) Compromised IQ: *impaired premorbid IQ and impaired cognition*	SSD 1) 31.66 ± 8.20 (79) SSD 2) 30.93 ± 8.14 (69) SSD 3) 39.98 ± 10.78 (39) HC: 32.82 ± 9.73 (125)	Current IQ, Education, Premorbid IQ

Clinical differences between cognitive subgroups were significant at *p* < .05; *BADS* Behavioural Assessment of the Dysexecutive Syndrome; *BD* bipolar disorder; *ePIQ* estimated premorbid IQ; *IQ* intelligence quotient; *ISLT* International Shopping List Task; *N/A* not available; *RBMT* Rivermead Behavioural Memory Test; *RO-SSD* recent-onset schizophrenia-spectrum disorder; *SD* standard deviation; *SSD* schizophrenia-spectrum disorders; *WMS-III* Wechsler Memory Scale 3rd Edition. * indicates that the study included longitudinal data

**Table 2:** Summary of neuroimaging methods and findings

**Authors**	**Neuroimaging Method**	**Brain Morphology**	**Brain Space/Atlas**	**Brain Regions**	**Cognitive Subgroups**	**Findings between subgroups**	**Findings between subgroups and healthy controls**
(Alonso-Lana et al., 2016)	1.5 T MRI	Volume	MNI	Whole brain	1) Impaired 2) Preserved	No significant difference in volume between cognitively preserved and impaired BD	Cognitively preserved BD had significantly reduced GMV in a single small cluster in the right precentral gyrus, and reduced WMV bilaterally, in the inferior occipito-frontal and uncinate fasciculus to the genu of the corpus callosum, and left inferior frontal cortex, compared to HC (no comparison with cognitively impaired subgroup)
(Ayesa-Arriola et al., 2013)*	1.5 T MRI	Volume	Talairach	Whole brain	1) Non-deficit 2) Deficit	No significant differences between subgroups in any of the brain regions at baseline. Deficit group showed greater total GMV and parietal lobe volume decrease over time than the non-deficit group	N/A
(Cobia et al., 2011)	1.5 T MRI	Thickness	N/A	Frontal, occipital, parietal, and temporal regions	1) Neuropsychologically Impaired 2) Neuropsychologically Near normal	Differences between clusters did not survive FDR correction for vertex-wise analyses. For the ROI analysis, the neuropsychologically impaired subgroup had significantly thinner bilateral temporal and occipital, and right parietal regions than the neuropsychologically near-normal subgroup	Compared with HC, the neuropsychologically impaired had widespread cortical thinning. Compared with HC, the neuropsychologically near-normal had milder thinning which did not survive FDR correction
(Colibazzi et al., 2013)	1.5 T MRI	Volume & Thickness	Talairach	Whole brain	1) Neuropsychologically impaired 2) Neuropsychologically near-normal	No significant difference in cortical thickness between subgroups. No within group comparisons on GMV. Neuropsychologically impaired subgroup had reduced local WMV underlying the perisylvian cortices (uncorrected)	Reduced GMV in the neuropsychologically impaired patients compared to HC in the MFG, IFG, precentral, postcentral, supramarginal, superior temporal and middle temporal gyri, and reduced WMV in the right hemisphere. The neuropsychologically near-normal had GMV reductions along the middle frontal and posterior cingulate gyrus, and minimal WMV reductions. Both subgroups had greater perisylvian cortex cortical thickness, and reduced SFG cortical thickness
(Czepielewski et al., 2017)	1.5 T MRI	Volume & Thickness	Destrieux	Whole brain	1) IQ + /COG + 2) IQ + /COG − 3) IQ − /COG −	SSD with IQ-/COG- had smaller TBV and ICV than IQ + /COG-, and larger whole WMV than the other subgroups SSD with IQ-/COG- had smaller total GMV and cortical GMV than each of the other groups SSD with IQ + /COG- had smaller total GMV than SSD with IQ + /COG + , and larger WMV than IQ + /COG + IQ-/COG- showed reduced cortical thickness compared to SSD with IQ + / COG + SSD with IQ-/COG- also had smaller insula volume than SSD with IQ + /COG +	SSD with IQ-/COG- had larger WMV, and smaller TBV and ICV than HC SSD with IQ + /COG + had smaller TBV than HC, even when controlling for ICV SSD with IQ-/COG- and IQ + /COG- had smaller total GMV and cortical GMV than HC SSD with impaired cognition whether or not they had impaired premorbid IQ (IQ-/COG- and IQ + /COG-) had significantly thinner cortex than HC IQ + /COG- and IQ-/ COG- had smaller anterior insula volume than HC IQ + /COG- had larger WMV than HC
(Geisler et al., 2015)	1.5 T MRI	Volume & Thickness	N/A	Whole brain	1) Diminished verbal fluency 2) Diminished verbal and poor motor control 3) Diminished face memory and slowed processing 4) Diminished intellectual function	Diminished intellectual function subgroup had sig. reduced cortical thickness in the right precentral area compared to diminished verbal fluency subgroup	*Diminished verbal fluency*) Sig. decrease in cortical thickness in the supramarginal gyrus *Diminished verbal and poor motor control*) No effects on cortical thickness, but reduced right hippocampal volume *Diminished face memory and slowed processing*) Sig. reductions in cortical thickness in lingual gyrus, occipital lobe, left superior frontal, rostral anterior cingulate and middle temporal gyrus *Diminished intellectual function*) Widespread reductions across both hemispheres
(Gould et al., 2014)	1.5 T MRI	Volume	Talairach	Whole brain	1) Cognitively impaired 2) Cognitively preserved	Subgroups could be distinguished from each other based on combined GMV and WMV, and total GMV (and not WMV) with 56% and 59% accuracy, respectively. When subgroups were separated based on sex, accuracy in the female group increased, and WMV only in males became non-significant	Cognitive deficit subgroup could be distinguished from HC with 72% (GMV & WMV), 70% (GMV), and 64% (WMV). Similar values for females and males, WMV not significant for males Cognitively spared subgroup could be distinguished from HC with 67% (GMV & WMV), 63% (GMV), and 59% (WMV). Similar values for females and males, WMV not significant for females
(Guimond et al., 2016)	3 T MRI	Volume & Thickness	LPBA40	Frontal region, hippocampus, and parahippocampus	1) Low to mild 2) Moderate to severe	Moderate to severe subgroup had thinner cortices in the left frontal lobe (MFG, IFG, OFG, precentral gyrus) and PHG, than the low to mild subgroup. No difference in hippocampal volumes between subgroups	Significantly thinner cortices in the moderate to severe group compared to HC in the left frontal lobe (IFG, OFG, precentral gyrus) and bilateral PHG. Low to mild group had thinner bilateral PHG and precentral gyrus than HC. No difference in hippocampal volumes between subgroups and HC
(Ho et al., 2020)*	3 T MRI	Volume & Thickness	Desikan-Killiany	Whole brain	1) Preserved IQ 2) Deteriorated IQ 3) Compromised IQ	Minimal between subgroup differences across global and regional analyses of volume and thickness	Consistent volume and thickness reductions in both preserved and deteriorated subgroups in comparison with healthy controls across both global and regional assessments
(Ortiz-Gil et al., 2011)	1.5 T MRI	Volume	MNI	Whole brain	1) Impaired 2) Preserved	No significant difference in brain volume between subgroups	Cognitively preserved subgroup had significantly less GMV than HC from the orbital and medial prefrontal cortex to the anterior cingulate gyrus. No significant difference in WMV between preserved subgroup and healthy controls (no comparison with cognitively impaired subgroup)
(Poletti et al., 2014)	3 T MRI	Volume	MNI	Whole brain	1) Bad performers 2) Good performers	Poor performers had significantly less GMV in the inferior frontal gyrus than good performers. No significant difference in WMV	N/A
(Rusch et al., 2007)	1.5 T MRI	Volume	N/A	Frontal region, thalamus, whole brain	1) Poor WCST performers 2) High WCST performers	Patients with poor WSCT performance, as compared to patients with good performance, had reduced GMV in the dorsolateral prefrontal cortex bilaterally. No WMV differences in the frontal region	HC had greater total GMV and left dorsolateral prefrontal cortex volume than patient groups. WMV comparisons not assessed in relation to controls
(Shepherd et al., 2015)	3 T MRI	Volume	MNI	Whole brain	1) Executively deficit 2) Executively spared	Executive deficit subgroup showed GMV reductions in the right inferior frontal, post central and precentral gyri relative to executively spared subgroup. No significant differences in WMV	Executive deficit subgroup had GMV reductions in the bilateral superior and medial frontal gyri, right inferior opercula gyri and hippocampus relative to controls. Executively spared had GMV reductions in the right precuneus and left superior and medial orbital frontal gyri relative to controls. No significant differences in WMV
(Torres et al., 1997)	1.5 T MRI	Volume	Talairach	Hippocampus, temporal region, whole brain	1) Low memory 2) High memory	No significant differences between subgroups in any of the brain regions	N/A
(Van Rheenen et al., 2018)	1.5 T MRI	Volume, Thickness, & Surface Area	Desikan-Killiany	Whole brain	1) Preserved IQ 2) Deteriorated IQ 3) Compromised IQ	Compared to deteriorated and preserved patients, compromised subgroup showed unique volumetric reductions in left lateral orbitofrontal cortex, PHG, temporal pole, and right pars triangularis, and thinner PHG and left rostral anterior cingulate. Compared to the deteriorated subgroup, compromised showed volumetric reductions in right lateral occipital gyrus and bilateral superior frontal region, and thinner right temporal pole. No significant differences in WMV	Whole cortex, subcortical, and regional volume and thickness reductions were evident in all subgroups compared to controls. No significant differences in WMV
(Vaskinn et al., 2015)	1.5 T MRI	Volume, Thickness, & Surface Area	Desikan-Killiany	Accumbens, amygdala, caudate, cerebellum, hippocampus, pallidum, putamen, thalamus	1) Intellectually normal 2) Intellectually superior 3) Intellectually low	No significant differences in brain volume (GMV), thickness, or surface area between subgroups	No significant differences in brain volume, thickness, or surface area between subgroups and HC
(Weinberg et al., 2016)	3 T MRI	Volume	Desikan-Killiany	Whole brain	1) Putatively Preserved 2) Moderately Deteriorated 3) Severely Deteriorated 4) Compromised	The severely deteriorated group had significantly smaller banks of superior temporal sulcus, lingual gyrus, and hippocampal volumes compared to the preserved group and smaller lingual and supramarginal gyri and superior temporal volumes than the moderately deteriorated group. No significant differences in WMV	Patient subgroups had lower inferior parietal volumes than controls. Deteriorated subgroups had decreased insula volumes compared to HC. Severely deteriorated group had the most extensive abnormalities compared with controls, across total cortex, total grey, cortical white matter, hippocampus, and increased lateral ventricles (the compromised subgroup was not included in any analyses)
(Wexler et al., 2009)	1.5 T MRI	Volume	N/A	Orbitofrontal, dorsal prefrontal, premotor, subgenual, sensorimotor, midtemporal, parietal, and inferior occipital subregions, amygdala, cerebellum, hippocampus, thalamus	1) Neuropsychologically Impaired 2) Neuropsychologically Near Normal	No significant GMV differences between subgroups in any of the brain regions. The neuropsychologically impaired subgroup had significantly reduced WMV of the sensorimotor, parietal-occipital, inferior occipital regions	Ventricular size (neuropsychologically impaired/near-normal < HC), grey matter (neuropsychologically impaired/near-normal < HC), thalamic volume (neuropsychologically impaired < HC), amygdala volume (neuropsychologically near-normal < HC), hippocampus (neuropsychologically impaired < HC), cerebellum volume (neuropsychologically impaired/near-normal = HC). Neuropsychologically impaired subgroup had less WMV in the dorsal prefrontal, premotor, sensorimotor, parietal-occipital, orbito-frontal, subgenual regions, and inferior occipital region
(Woodward & Heckers, 2015)	3 T MRI	Volume	N/A	Whole brain	1) Neuropsychologically normal 2) Neuropsychologically impaired 2a) Compromised 2b) Deteriorated	ICV and TBV reduced in neuropsychologically impaired subgroup, compared to neuropsychologically near-normal. Compromised had reduced ICV compared to all groups, and reduced TBV compared to neuropsychologically near-normal. No significant difference in WMV between subgroups	Compromised had reduced ICV and TBV compared to HC. Neuropsychologically near-normal, neuropsychologically impaired and deteriorated groups had reduced WMV and TBV (ICV adjusted) compared to HC
(Yasuda et al., 2020)	1.5 & 3 T MRIs	Volume	Desikan-Killiany	Whole brain	1) Preserved IQ 2) Deteriorated IQ 3) Compromised IQ	Whole brain and total cortical grey matter, right fusiform gyrus, left pars orbitalis gyrus, right pars triangularis, left superior temporal gyrus and left insula volumes, and bilateral cortical thickness were reduced in the deteriorated group compared to preserved subgroup	Whole brain and total cortical grey matter, right fusiform gyrus, left pars orbitalis gyrus, right pars triangularis, left superior temporal gyrus and left insula volumes, and bilateral cortical thickness were reduced in the deteriorated group compared to healthy controls. Both subgroups had increased left lateral ventricle, right putamen and left pallidum, and reduced bilateral hippocampus, left precentral gyrus, right rostral middle frontal gyrus, and bilateral superior frontal gyrus volumes compared with controls

These findings are brief summaries, for a complete breakdown of reported findings, refer to Supplementary Table 3. Subgroup naming conventions are based on those used in each paper. *BD* bipolar disorder; *FDR* false discovery rate; *GMV* grey matter volume; *HC* healthy control; *ICV* intracranial volume; *IFG* inferior frontal gyrus; *IQ + /COG +*  average premorbid and current IQ; *IQ + /COG-* average premorbid IQ, impaired current IQ; *IQ-/COG-* impaired premorbid and current IQ; *MFG* medial frontal gyrus; *MNI* Montreal Neurological Institute; *MRI* magnetic resonance imaging; *N/A* not available; *OFG* orbitofrontal gyrus; *PHG* parahippocampal gyrus; *ROI* region of interest; *SFG* superior frontal gyrus; *SSD* schizophrenia-spectrum disorders; *TBV* total brain volume; *WCST* Wisconsin Card Sorting Test. * indicates that the study included longitudinal data

### Evaluation of Study Quality

An evaluation of the quality of each study is presented in supplementary Table S1; each study was inspected as to whether i) statistical correction was applied where appropriate, ii) automated methods for imaging analyses were used, iii) healthy control comparison groups were included in the sample, iv) statistical comparisons were made between all available (sub)groups, v) if the imaging parameters/protocol were clearly described to enable replication and vi) if cognitive measures/protocol were clearly described to enable replication. Either a zero or one was awarded for each of the respective six checks of quality, and the overall average score was 5.50, indicating a high quality generally to the studies included in this review.

### Explanation of Study Synthesis

A synthesis of results is provided in separate sections below, covering studies that report on two, three or four cognitive subgroups (supplementary Table S2). Each section begins with a brief summary of the cognitive subgrouping methodologies used in the studies within that section. Findings are then systematically grouped and presented by examination of volume, thickness, surface area, within which comparisons between subgroups and healthy controls are first detailed followed by comparisons between subgroups themselves. The terms recent-onset (i.e. RO-SSD/RO-BD) and established are used throughout the review to clearly delineate between illness stages. Sample sizes for each subgroup are only presented at the first instance a study is mentioned. This synthesis structure was used to facilitate readability and reduce confusion in the context of the high degree of methodological heterogeneity in this field. From the 20 studies, all reported the brain morphology findings between cognitive subgroups and healthy controls are presented in supplementary Table S3, ordered alphabetically by the relevant lobe and region.

## Results

### Search Selection

**Fig. 2:**
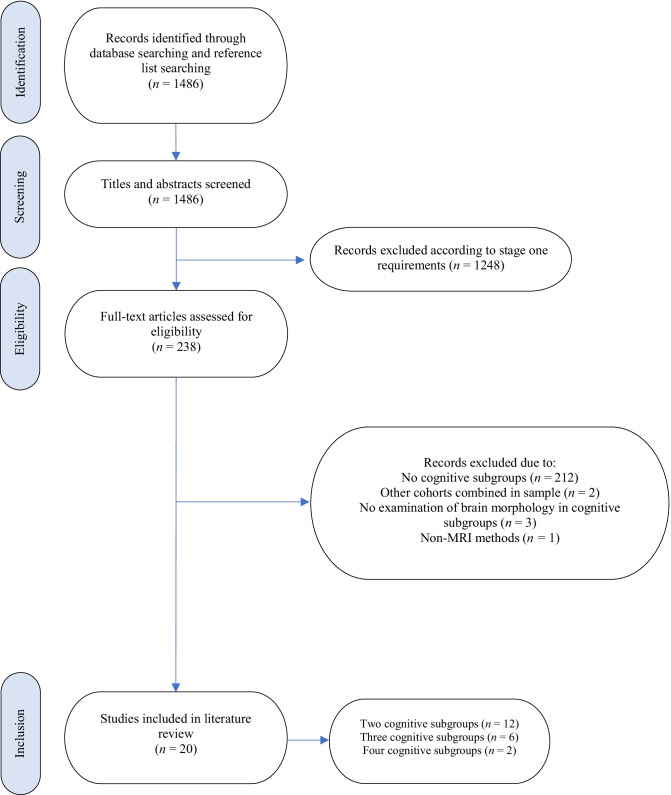
Flowchart of study selection

The search strategy yielded a total of 1486 records, from which 20 studies met the inclusion criteria. A flowchart of this selection process is presented in Fig. 2. A summary of study characteristics and results are provided in Tables 1 and 2. Of the 20 studies, 15 investigated SSD, three examined mixed (cross-diagnostic) samples of SSD and BD samples, and the remaining two investigated BD and RO-SSD, respectively. Seventeen of the 20 studies included healthy control comparison groups in their analyses. The majority of studies (n = 15) used predetermined performance cut-off scores to define their cognitive subgroups, whilst the remaining (n = 5) used data-driven approaches such as clustering analysis.

### Findings from Analyses of Two Cognitive Subgroups

#### Summary

Twelve studies identified two cognitive subgroups, 10 of which used cognitive cut-off scores (Alonso-Lana et al., 2016; Ayesa-Arriola et al., 2013; Colibazzi et al., 2013; Guimond et al., 2016; Ortiz-Gil et al., 2011; Poletti et al., 2014; Rusch et al., 2007; Shepherd et al., 2015; Torres et al., 1997; Wexler et al., 2009) and two used data-driven approaches (Cobia et al., 2011; Gould et al., 2014). Despite variations in naming conventions, these studies all report on cognitive subgroups that could be considered to have cognition that is either relatively intact or impaired. For ease of interpretation, we have adopted these naming conventions to report all relevant findings in these studies going forward.

Poletti et al. (2014), Rusch et al. (2007), Guimond et al. (2016) and Torres et al. (1997) each classified SSD patients in their study into either ‘high’ or ‘low’ performing subgroups based on executive function, working memory and verbal memory scores, respectively. Ortiz-Gil et al. (2011) and Alonso-Lana et al. (2016) categorised their respective SSD and BD patients into cognitively impaired and preserved subgroups based on their performance on the measures of executive function, verbal memory/learning, visual memory/learning. Colibazzi et al. (2013) and Wexler et al. (2009) both assessed attention, and verbal and working memory in SSD patients in their study, and classified them into impaired and relatively intact cognitive subgroups. Ayesa-Arriola et al. (2013) employed cut-off scores to classify their RO-SSD sample into a non-deficit and deficit subgroup based on a global cognitive functioning index summarizing standardized performance across eight cognitive domains. In their cross-diagnostic study, Shepherd et al. (2015) used cognitive cut-off scores to group SSD and BD patients into cognitively impaired and relatively intact subgroups based on working memory scores. Finally, the studies by Gould et al. (2014) and Cobia et al. (2011) employed data-driven approaches to classify SSD cognitive subgroups based on a wide range of neuropsychological tests.

Eight studies examined volume (Alonso-Lana et al., 2016; Ayesa-Arriola et al., 2013; Gould et al., 2014; Ortiz-Gil et al., 2011; Poletti et al., 2014; Rusch et al., 2007; Shepherd et al., 2015; Torres et al., 1997; Wexler et al., 2009), one cortical thickness (Cobia et al., 2011), and two examined volume and cortical thickness (Colibazzi et al., 2013; Guimond et al., 2016).

#### Volume (Two Subgroup Studies)

Nine of the 12 studies compared the volume of cognitive subgroups to healthy control samples. Rusch et al. (2007) observed that both relatively intact and impaired cognitive subgroups of SSD patients had significantly less total grey matter and left dorsolateral prefrontal cortex volume than healthy controls. White matter volume was not assessed in relation to healthy controls in this study. Ortiz-Gil et al. (2011) reported that the relatively intact SSD subgroup had significantly less grey matter volume than healthy controls in the regions between the orbital and medial prefrontal cortex to the anterior cingulate gyrus; no differences were identified between the cognitively impaired SSD subgroup and healthy controls. For reasons unspecified, only comparisons of white matter volume were made between the relatively intact SSD subgroup and healthy controls, to which no differences were observed.

Wexler et al. (2009) observed that their cognitively impaired subgroup of SSD patients had significantly larger ventricular volume in all compartments compared to healthy controls, while the relatively intact cognitive subgroup only had larger third ventricle volume. The two cognitive subgroups also showed reduced cortical grey matter volume relative to healthy controls in all regions except the orbitofrontal cortex, where only the relatively intact cognitive subgroup showed reductions. The impaired subgroup had further subcortical reductions in hippocampal and thalamic grey matter volume, while only the relatively intact cognitive subgroup differed to healthy controls in amygdala volume. In contrast, Guimond et al. (2016) observed no significant difference in hippocampal volumes between their healthy controls and their impaired and relatively intact subgroups. In terms of white matter reductions, only the cognitively impaired subgroup had reductions compared to healthy controls, found in the dorsal prefrontal, premotor, sensorimotor, parietal-occipital, orbito-frontal, subgenual, and inferior occipital regions. There were no reductions in cerebellar volume in either subgroup. In another study which formed subgroups based on working memory performance, Colibazzi et al. (2013) found that compared to healthy controls, the cognitively impaired subgroup of SSD patients had reduced medial frontal, inferior frontal, precentral, postcentral, supramarginal, superior temporal, and middle temporal gyri grey matter volume. This subgroup also had reduced white matter volume across the right hemisphere. In contrast, the relatively intact cognitive subgroup had grey matter volume reductions only in the middle frontal and posterior cingulate gyrus, and generalised, but minimal white matter volume reductions.

Gould et al. (2014) took a data-driven approach and applied support vector machine classification (using 200 resample iterations and leave-two out cross validation) to grey and white matter volume, separately and collectively, to determine if their cognitive subgroups of SSD patients were neuroanatomically different from healthy controls. This approach was able to distinguish cognitively impaired patients from healthy controls with 72% (grey and white matter volume), 70% (grey matter volume only), and 64% (white matter volume only) accuracy. Similarly, the relatively intact cognitive subgroup was distinguished from healthy controls with 67% (grey and white matter volume), 63% (grey matter volume only), and 59% (white matter volume only) accuracy. That is, the analysis was able to accurately distinguish between each of the subgroups and healthy controls at a rate higher than chance, based on the grey, white, and combined grey and white matter profiles.

In Alonso-Lana et al. (2016) sample of BD patients, the relatively intact cognitive subgroup showed a significant reduction in right precentral gyrus grey matter volume in comparison to healthy controls. The relatively intact cognitive also showed clusters of significantly reduced white matter volume bilaterally, from the inferior occipito-frontal and uncinate fasciculus to the genu of the corpus callosum, and in a small cluster adjacent to the inferior frontal cortex. For unspecified reasons, no comparisons of either grey or white matter volume were made between the cognitively impaired BD subgroup and healthy controls.

In comparing a cross-diagnostic SSD and BD sample with healthy controls, Shepherd et al. (2015) reported that the cognitively impaired subgroup had grey matter reductions in the bilateral superior and medial frontal gyri, right inferior opercular gyri, and hippocampus, while the relatively intact cognitive subgroup had grey matter reductions in the right precuneus, and left superior and medial orbital frontal gyri. With regards to white matter, there were no significant differences between cognitive subgroups and healthy controls.

In comparing between cognitive subgroups themselves, Rusch et al. (2007) reported that compared to their relatively intact cognitive subgroup, their cognitively impaired subgroup had significantly reduced dorsolateral prefrontal cortex volume bilaterally. Similarly, Poletti et al. (2014) reported that their impaired subgroup had reduced inferior frontal gyrus volume. Contrary to these findings, Torres et al. (1997), Wexler et al. (2009), Ortiz-Gil et al. (2011), Guimond et al. (2016), and Alonso-Lana et al. (2016) did not find any significant grey matter volumetric differences between cognitive subgroups despite finding volumetric differences between these subgroups and healthy controls. Of these studies, only Wexler et al. observed significant differences in white matter volume between their cognitive subgroups; the cognitively impaired subgroup had significantly reduced white matter volume to the relatively intact cognitive subgroup in sensorimotor and parietal-occipital regions.

With respect to SSD subgroups formed by data-driven methods, Gould et al. (2014) showed that cognitively impaired and relatively intact subgroups could be distinguished at a rate higher than chance using combined grey and white matter volume (56%) and grey matter volume only profiles (59%), but not white matter volume. When the subgroups were separated based on sex, this accuracy was similar for males (60% for grey and white volume, and 58% for grey matter volume only) but was further increased for females (83% for grey and white volume, and 65% for grey matter volume only). When stratified by sex, white matter-only profiles could also distinguish groups with much higher accuracy (77%), suggesting that sex differences in brain morphology are especially important for understanding relationships with cognitive performance in psychotic disorders.

In the only study with a RO-SSD sample, Ayesa-Arriola et al. (2013) examined brain volumetric differences at two timepoints, baseline and a three year follow-up. At baseline patients were classified into subgroups, and there were no significant differences between relatively intact and impaired cognitive subgroups in whole brain volume, and total, cortical, and subcortical grey and white matter. However, at follow-up, the cognitively impaired subgroup exhibited significantly lower total grey matter and parietal lobe volume than the relatively intact cognitive subgroup. This study did not include a healthy control group, thus comparisons with normative change were not reported.

Lastly, in the cross-diagnostic study by Shepherd et al. (2015), between-subgroup comparisons revealed significant reductions in the right inferior frontal, precentral, and postcentral gyri in the cognitively impaired subgroup compared to the relatively intact cognitive subgroup. No significant differences in white matter volume were evident between subgroups.

#### Cortical Thickness (Two Subgroup Studies)

Three of the 12 studies with two cognitive subgroups compared the cortical thickness of cognitive subgroups to that of healthy control samples. Colibazzi et al. (2013) reported that both cognitively impaired and relatively intact subgroups had thicker perisylvian cortices, but thinner superior frontal gyri relative to healthy controls. Cobia et al. (2011) also reported significantly thinner frontal, temporal, occipital and parietal lobes in the cognitively impaired group compared to healthy controls, specifically in the pars orbitalis, lateral orbital gyrus, posterior superior frontal, primary and association sensorimotor cortices, lateral occipital, superior parietal, paracentral, cuneus, lingual, parahippocampal, fusiform, insula, and supramarginal regions. In contrast, those with relatively intact cognition were not significantly different to healthy controls in these vertex-wise analyses. This pattern of quite generalised and widespread thickness reductions in the more impaired subgroup relative to controls was consistent with the findings of Guimond et al. (2016), who also identified significantly thinner left inferior frontal, orbito-frontal, and precentral gyri, and bilateral parahippocampal gyri in their cognitively impaired subgroup compared to healthy controls. The relatively intact cognitive subgroup in this study was found to have thinner cortices, limited to bilateral parahippocampal gyri and precentral gyri regions.

Comparing the cognitive subgroups to each other, Guimond et al. (2016) reported that their cognitively impaired patients displayed significantly thinner cortex in the medial, inferior, and occipital frontal gyrus, precentral gyrus, and parahippocampal gyrus compared to the relatively intact subgroup. Similarly, Cobia et al. (2011) observed the cognitively impaired subgroup to have significantly thinner cortex in bilateral temporal and occipital, and right parietal regions. With due consideration, the between-subgroup findings reported by Guimond et al. (2016) must be interpreted with caution, as they did not correct for multiple comparison in subgroup analyses. In contrast to these findings, Colibazzi et al. (2013) found no significant differences in cortical thickness between relatively intact and impaired cognitive subgroups.

### Findings from Analyses of Three Cognitive Subgroups

#### Summary

Six studies identified three cognitive subgroups, of which five used predetermined cognitive cut-off scores to derive the subgroups (Czepielewski et al., 2017; Ho et al., 2020; Vaskinn et al., 2015; Woodward & Heckers, 2015; Yasuda et al., 2020) and one used a data-driven approach (Van Rheenen et al., 2018). The majority of studies investigated putative cognitive symptom trajectories, derived from estimated premorbid and current IQ scores. These subgroups were considered to be cognitively preserved, compromised, or deteriorated in comparison to healthy controls.

On the other hand, Vaskinn et al. (2015) used full scale IQ scores to delineate subgroups that reflected normal, superior, and low intellectual functioning versus healthy controls, although the intellectually low subgroup was excluded from their analyses. Of these six studies, two examined volume only (Woodward and Heckers, Yasuda et al.), two examined volume and cortical thickness (Czepielewski et al., Ho et al.), and two examined volume, cortical thickness, and cortical surface area (Van Rheenen et al., 2018; Vaskinn et al., 2015). The Vaskinn et al. study reported no significant differences between SSD subgroups and healthy controls, or between SSD subgroups themselves with respect to brain volume, cortical thickness, or surface area. Thus, the following sub-sections synthesise the findings of the other five studies only. Furthermore, as Ho et al.’s compromised subgroup comprised only six participants, it was excluded from all analyses.

#### Volume (Three Subgroups)

Relative to healthy controls, all subgroups in the study of Van Rheenen et al. (2018) were reported to have significantly less total grey matter, whilst both Czepielewski et al. (2017) and Yasuda et al. (2020) observed significantly less volume in total and cortical grey matter in their compromised or deteriorated subgroups only. Yasuda et al. also reported reduced total brain volume in their deteriorated subgroup, whereas Czepielewski et al. indicated that their compromised subgroup had smaller absolute total brain and intracranial volume than their healthy controls. This finding contrasted Van Rheenen et al.’s observation that neither of these brain measures differed between any cognitive subgroup and healthy controls. However, when intracranial volume was adjusted for total brain volume, Van Rheenen et al. observed significant reductions in all subgroups compared to healthy controls. A reduction of this nature was observed in only the cognitively preserved subgroup in the Czepielewski et al. study.

In terms of white matter volume, Van Rheenen et al. observed no significant differences in white matter volume between all three subgroups and healthy controls, whilst Czepielewski et al. observed larger white matter volumes in the compromised and deteriorated subgroups.

In their regional analyses, Yasuda et al. (2020) reported reductions relative to healthy controls in both of their subgroups, across the hippocampus bilaterally, left precentral gyrus, right rostral middle frontal gyrus, and superior frontal gyrus bilaterally. Uniquely in their deteriorated subgroup, Yasuda et al. observed significantly reduced bilateral fusiform gyrus, bilateral superior temporal gyrus, right middle temporal gyrus, bilateral parahippocampal gyrus, left insula, right inferior parietal lobule, left pars orbitalis gyrus, bilateral pars triangularis, and right medial orbitofrontal cortex. Van Rheenen et al. (2018) also reported that relative to healthy controls, all subgroups had less volume of the left inferior parietal cortex, right supramarginal gyrus, and bilateral volume of the hippocampus, frontal pole, middle temporal gyrus and pars orbitalis. Further, Van Rheenen et al.’s compromised and deteriorated subgroups showed significant reductions in right precentral gyrus and left lateral orbitofrontal regions, whilst these subgroups in the study by Czepielewski et al. (2017) had reduced anterior insula volumes.

In their compromised subgroup only (relative to healthy controls), Van Rheenen et al. also identified specific bilateral reductions in the rostral middle frontal region, and left temporal pole, inferior temporal, superior frontal, and parahippocampal gyri, and the right lateral occipital and superior temporal gyri, right lateral and medial orbitofrontal cortices and right pars triangularis. They further observed a reduction in volume that was unique to the preserved subgroup, and that was found in the left superior temporal gyrus and right inferior temporal gyrus. Notably, both Van Rheenen et al. and Yasuda et al., also observed increased volume in select regions relative to healthy controls; including subcortical structures of the putamen and pallidum in each of Van Rheenen et al.’s subgroups, and left lateral ventricle, right putamen, and left pallidum in both Yasuda et al.’s deteriorated and preserved subgroups.

In their cross-diagnostic sample of patients with SSD and BD, Woodward and Heckers (2015) observed that cognitively compromised patients had significantly reduced intracranial and absolute total brain volume in comparison with healthy controls, whilst the deteriorated and preserved subgroup were no different. When analyses were adjusted for intracranial volume, significant reductions in total brain volume and total white matter—but not total grey matter—were now evident in both preserved and deteriorated subgroups, and no longer in the compromised subgroup. Similarly, Ho et al. (2020) observed that after adjusting for intracranial volume, their cross-diagnostic deteriorated and preserved subgroups had reduced absolute total brain volume, as well as total cortical grey matter volume, relative to healthy controls. Furthermore, their deteriorated subgroup also demonstrated reduced total subcortical grey matter and cortical white matter volume.

In voxel-based morphometry (VBM) analyses in comparison to healthy controls, Woodward and Heckers et al. did not identify any localised changes in grey matter in their cognitively preserved subgroup. They did however, observe that both compromised and deteriorated subgroups exhibited reductions of grey matter volume in the thalamus and medial temporal lobe. Further, the deteriorated subgroup had a greater magnitude of volume loss in the precentral gyrus, whereas the compromised subgroup had a greater magnitude of volume loss in the left superior temporal gyrus and cerebellum. In contrast, Ho et al. (2020) reported reduced bilateral hippocampal volume, reduced thalamic volume and enlarged ventricles in their deteriorated and preserved subgroups, and reduced left amygdala in the compromised subgroup. Longitudinally, Ho et al. reported a significant subgroup-by-time interaction for the hippocampus volume bilaterally in the deteriorated subgroup relative to healthy controls.

With regards to white matter volume compared to healthy controls, Woodward and Heckers et al.’s preserved subgroup had reductions in the frontal lobe, beneath the superior and middle frontal gyri, genu of the corpus callosum, and in the left medial parietal lobe. In the impaired subgroups, the deteriorated subgroup had significant white matter reductions in the centrum semiovale, periventricular regions, lateral ventricles and corpus callosum, while the compromised subgroup had significant volume loss limited to posterior periventricular regions, splenium of the corpus callosum, cerebral peduncles, and pons.

Comparing volume between cognitive subgroups, Czepielewski et al. (2017) showed that the deteriorated subgroup had significantly reduced total grey matter and increased white matter volume compared to the preserved subgroup. The deteriorated subgroup in the study of Yasuda et al. (2020) had similar reductions in total and cortical grey matter volume, as well as bilateral cortical thickness, right fusiform gyrus, left pars orbitalis gyrus, right pars triangularis, left superior temporal gyrus, and left insula. Increased right lateral ventricle volume was observed relative to the preserved subgroup.

Czepielewski et al.’s compromised subgroup also had significantly reduced global and cortical grey matter volumes and increased white matter volume compared to the deteriorated and preserved subgroups, reduced total brain volume and intracranial volume compared to the deteriorated subgroup, and smaller insula volume than the preserved subgroup. In Van Rheenen et al.’s (2018) study, the compromised subgroup had unique volume reductions in the left and right cortex globally, and regionally in the lateral orbitofrontal, parahippocampal gyrus, temporal pole, and right pars triangularis compared to the deteriorated and preserved subgroups. Compromised patients also demonstrated significant reductions in global grey matter, as well as regional volume reductions in the bilateral hippocampus, right lateral occipital gyrus, and bilateral superior frontal region relative to the deteriorated subgroup. No significant differences in white matter volume were observed between subgroups. Given that patients with a premorbid IQ less than 75 were not included in this study, it is possible that this resulted in less pronounced differences between the compromised subgroup and the other subgroups compared to those seen in the Czepielewski et al.’s study.

In the study by Woodward and Heckers (2015), their compromised subgroup had significantly reduced intracranial volume compared to both deteriorated and preserved subgroups and significantly reduced total brain volume relative to the preserved subgroup. When volumes were adjusted for intracranial volume, there were no significant differences between any of the subgroups in grey, white, or total brain volume. Ho et al. (2020) observed reduced bilateral hippocampal volume in the deteriorated subgroup, and reduced amygdala volume in the compromised subgroup, both relative to the preserved subgroup.

#### Cortical Thickness (Three Subgroups)

With regards to cortical thickness in cognitive subgroups compared to controls, Czepielewski et al. (2017) observed that both the compromised and deteriorated subgroups—and not the preserved subgroup—had significantly thinner cortices globally. In contrast, Van Rheenen et al. (2018) reported that all three subgroups exhibited significantly thinner cortices than healthy controls. Further, Yasuda et al. (2020) observed reduced left and right total cortical thickness in their deteriorated subgroup. Van Rheenen et al. also carried out regional analyses, indicating widespread thickness reductions relative to controls in all three groups in the left rostral anterior cingulate and right supramarginal gyrus, and broadly across several frontal and temporal regions. Specific thickness reductions relative to controls were also evident in the compromised group in the parahippocampus bilaterally, transverse temporal cortex, left entorhinal, and bank of the superior temporal sulcus, and in the right inferior parietal and left supramarginal regions. In the deteriorated subgroup, reductions in thickness were observed in the right inferior parietal and caudal anterior cingulate, and the left entorhinal cortex and precuneus compared to controls, and in the preserved subgroup in the right transverse temporal cortex, left bank of the superior temporal sulcus, and left isthmus of the cingulate. Both Ho et al. (2020) preserved and deteriorated subgroups demonstrated widespread thinning across both frontal and temporal regions, as well as the lateral occipital gyrus and inferior parietal cortex, relative to healthy controls. Cortical thinning of the lingual gyrus was common to both deteriorated and compromised subgroups, whilst reductions of the superior frontal gyrus and lateral orbitofrontal gyrus were unique to these subgroups, respectively.

When comparison of cortical thickness were conducted between the subgroups themselves, Czepielewski et al. (2017) reported that the compromised subgroup had significantly thinner cortices compared to the preserved subgroup. Van Rheenen et al. (2018) on the other hand, did not find between-subgroup differences in cortical thickness at a global level. Instead, Van Rheenen et al. observed more localised regional differences, where the compromised subgroup had significantly thinner cortices in the left rostral anterior cingulate and left parahippocampal gyrus relative to deteriorated and preserved subgroups. The compromised group also had significantly thinner cortices in the right temporal pole relative to deteriorated patients only. Ho et al. (2020) reported no between subgroup differences in cortical thickness, both cross-sectionally and longitudinally.

#### Cortical Surface Area (Three Subgroups)

Van Rheenen et al. (2018) examined surface area but found no differences between-subgroup or between any subgroup and healthy controls.

### Findings from Analyses Between Four Cognitive Subgroups

#### Summary

Two studies identified four cognitive subgroups, both of which were derived using data-driven approaches (Geisler et al., 2015; Weinberg et al., 2016) based on current cognitive functioning. Of these, one examined volume (Weinberg et al.) and one cortical thickness (Geisler et al.).

#### Volume (Four Subgroups)

Of the two studies with four cognitive subgroups, only Weinberg et al. (2016) examined volume in subgroups relative to healthy controls. They derived four subgroups reflecting putative cognitive symptom trajectories, namely putatively preserved, moderately deteriorated, severely deteriorated and compromised. It should be noted that the compromised subgroup was not included in the brain volume analyses given the small sample numbers in that subgroup. Relative to healthy controls, the putatively preserved and both deteriorated subgroups had significantly reduced inferior parietal volumes, while reduced insula volumes were evident only in both deteriorated subgroups. The most severely deteriorated group also had widespread volume reductions in several cortical regions compared to controls, as well as in the hippocampus, total cortex, and cortical grey and white matter volume.

Regarding volumetric differences between the subgroups themselves, Weinberg et al. (2016) observed that the severely deteriorated group had significantly smaller banks of superior temporal sulcus, lingual gyrus, and hippocampal volumes compared to the preserved subgroup, and smaller lingual and supramarginal gyri and superior temporal volumes compared to the moderately deteriorated subgroup.

#### Cortical Thickness (Four Subgroups)

Geisler et al. (2015) identified four differing profiles of cognitive impairment using data-driven clustering analysis of performance on several cognitive tests. These subgroups were classified as having diminished intellectual function diminished verbal fluency; diminished verbal and motor control, diminished face memory and slowed processing and diminished intellectual function. In an analysis focusing primarily on cortical thickness, compared to healthy controls no significant differences were observed in the cluster with diminished verbal memory and poor motor control. However, relative to healthy controls, reduced thickness of the supramarginal gyrus was evident in the subgroup with diminished verbal fluency; reductions in right hippocampal volume in the diminished verbal memory and poor motor control subgroup; and reductions in cortical thickness in the lingual gyrus, occipital lobe, left superior frontal, rostral anterior cingulate, and middle temporal gyrus, in the subgroup with reduced face memory and processing speed. Widespread thickness reductions across both hemispheres were also evident in the subgroup with diminished intellectual function. The only significant between-subgroup difference was observed between this subgroup and the subgroup with diminished verbal fluency, with former subgroup characterised by a significantly thinner right precentral region.

## Discussion

This review aimed to synthesize the evidence for differences in brain morphology between cognitive subgroups of SSD and BD, when examined in each disorder separately and cross-diagnostically. The identification of morphological differences between cognitive subgroups and healthy controls may be helpful to identify and differentiate specific brain morphological abnormalities more heavily associated with i) cognitive impairment, ii) the presence of SSD or BD, and iii) a compounding effect of both cognitive impairment and disease presence (Fig. 1a-c). It can also provide insight into whether the cognitive subgroups exhibit distinct patterns of brain morphology, or if brain morphological abnormalities in the subgroups follow a pattern of graded severity that accords with the extent of cognitive impairment. Our discussion of the findings in this context should be interpreted with the knowledge that there was a high degree of methodological heterogeneity between studies.

### Evidence for Brain Morphological Abnormalities more Heavily Associated with Cognitive Impairment, Independent of SSD or BD Disease Presence

In the absence of morphological differences between patients with relatively intact cognition and healthy controls, significant differences in the same measures between cognitively impaired patients and both of these groups can reveal which specific brain regions are more heavily associated with cognitive impairment, independent of the presence of SSD-BD spectrum disorders (Fig. 1a). Seven unique studies showed a pattern of significant differences in specific measures of brain morphology between cognitively impaired and intact patients, cognitively impaired patients and healthy controls, but not between healthy controls and the cognitively intact subgroup. Thus, there appear to be some specific, albeit limited, brain abnormalities that may be more strongly associated with cognitive impairment independent of SSD or BD diagnosis.

Indeed, cognitive impairment was found to be more strongly associated with thinner cortex in the frontal lobe globally and in the left middle frontal and orbitofrontal gyri (Guimond et al., 2016), as well as reduced white matter volume across the dorsolateral prefrontal, orbitofrontal, premotor, sensorimotor, and subgenual cingulate cortex (Wexler et al., 2009). It was also specifically associated with thinner temporal cortex globally (Cobia et al., 2011) and reduced grey matter volume of the hippocampus and banks of the superior temporal gyrus (Weinberg et al., 2016), as well as thinner left and right occipital cortex (Cobia et al., 2011), reduced grey matter volume of the lingual gyrus bilaterally (Weinberg et al., 2016), increased lateral ventricle volume (Yasuda et al., 2020), and reduced white matter volume of the parietal-occipital region bilaterally (Wexler et al., 2009). Reduced grey matter volume of the anterior insula (Czepielewski et al., 2017) and thinner cortex in the right parietal lobe were also associated with cognitive impairment (Cobia et al., 2011). Further, cognitive impairment was associated with reductions in total and cortical grey matter volume, bilateral and total cortical thickness (Czepielewski et al., 2017; Yasuda et al., 2020), total and cortical white volume (Czepielewski et al., 2017), as well as intracranial and total brain volume (Woodward & Heckers, 2015).

Six of the above seven studies characterized their cognitive subgroups based on current IQ performance or performance on a cognitive battery across many domains, such that they were relatively comparable in terms of cognitive impairment being generalized across domains. Although the occipital lobe (broadly) was uniquely associated with cognitive impairment in three separate studies, no two studies observed the same finding regarding specific brain regions uniquely associated with cognitive impairment. However, both Yasuda et al. (2020) and Czepielewski et al. (2017) did highlight total cortical grey matter volume and total cortical thickness as being more strongly associated with cognition. Notably, there were no findings relating to subcortical regions, or cortical surface area. Reductions in cortical thickness, rather than brain volume, were more commonly associated with cognitive impairment, a pertinent finding given that the number of studies in this review examining cortical thickness were less than half the total.

### Evidence for Brain Morphological Abnormalities Associated with SSD or BD Disease Presence, Independent of Cognitive Impairment

In the absence of specific brain morphology differences between cognitive subgroups themselves, significant differences in these regions between cognitive subgroups and healthy controls can reveal which brain morphology measures are associated with SSD or BD disease presence irrespective of cognitive impairment (Fig. 1b). Eleven unique studies reported findings meeting these criteria. In the frontal region, grey matter volume reductions uniquely associated with psychiatric disease were observed in the frontal pole and pars orbitalis (Van Rheenen et al., 2018), left medial and superior frontal gyrus (Colibazzi et al., 2013; Shepherd et al., 2015), and middle frontal gyrus. Widespread thinner cortex across the left and right frontal lobe was also associated (Cobia et al., 2011). In the parietal region, grey matter reductions uniquely associated with psychiatric disease were evident in the left and total inferior parietal lobe (Van Rheenen et al., 2018; Weinberg et al., 2016) as well as the right supramarginal gyrus (Van Rheenen et al., 2018). In the temporal lobe, grey matter volume reductions were observed in the hippocampus and middle temporal gyrus (Van Rheenen et al., 2018), as well as reductions in thickness of the hippocampus (Guimond et al., 2016). Increased third ventricle compartment volume was also uniquely associated (Wexler et al., 2009), as were reductions in total cortical grey matter (Ho et al., 2020), total grey matter volume (Ho et al., 2020; Rusch et al., 2007; Wexler et al., 2009; Woodward & Heckers, 2015), intracranial volume (Van Rheenen et al., 2018), total brain volume (Czepielewski et al., 2017), total brain volume correcting for intracranial volume (Czepielewski et al., 2017; Ho et al., 2020; Woodward & Heckers, 2015) and total white matter volume (Woodward & Heckers, 2015). Increased cortical thickness of the perisylvian cortex (Colibazzi et al., 2013) and increased volume of the pallidum and putamen (Van Rheenen et al., 2018) were also uniquely associated with psychiatric disease.

Across the ten studies, frontal lobe grey matter volume was associated with SSD or BD across five different studies, although there was no consistency in the specific region. Two studies were consistent in their implication of inferior parietal lobe grey matter volume reductions. Similarly, consistency between studies was found in global measures such as total grey matter volume (four studies) and total brain volume (corrected for intracranial volume; three studies). In comparison with the findings relating to brain morphology uniquely associated with cognitive impairment, the occipital lobe did not appear to be uniquely associated with the presence of disease. There was more evidence for an association of SSD or BD presence with grey matter volume reductions compared with reductions in cortical thickness, while surface area was not implicated at all.

### Evidence for brain morphological abnormalities reflecting the interactions of SSD or BD disease presence and cognitive impairment

Significant morphological abnormalities in both cognitively impaired and relatively intact subgroups compared to healthy controls and in the same regions between these cognitive subgroups themselves, may be indicative of an interaction between the presence of cognitive impairment and SSD and BD presence (Fig. 1c). Accordingly, four unique studies, all in SSD samples with two cognitive subgroups, reported findings that suggest a compounding effect of cognitive impairment in the presence of disease, as indicated by the graded effect of differences (in terms of effect size), in which the cognitively impaired subgroup had greater morphological deficits than the subgroup with relatively intact cognition, who in turn, had significantly greater deficits relative to healthy controls. These studies implicate cortical thickness of the left temporal lobe (Cobia et al., 2011), including the left precentral gyrus, parahippocampal gyrus (Guimond et al., 2016); as well as grey matter volume of the left dorsolateral prefrontal cortex grey matter volume (Rusch et al., 2007) and total grey, white, and whole brain volume (Gould et al., 2014). The evidence for this graded severity effect is relatively limited as there was no replication of effects of specific regions in which the effect was evident across studies.

In contrast to evidence of a severity grading in some studies, there were also limited studies reporting brain morphology patterns that were unique to particular cognitive subgroups. Indeed, four studies identified relationships in which the cognitively intact subgroup had morphological reductions relative to healthy controls that were not found when comparing the healthy controls and cognitively impaired patients. These include cortical thickness reductions in the right precentral gyrus (Guimond et al., 2016) and supramarginal gyrus (Geisler et al., 2015), and grey matter volume reductions in the right precuneus, right angular gyrus (Shepherd et al., 2015), and left superior and right inferior temporal gyri (Van Rheenen et al., 2018). However, it should be noted that beyond these four studies, the majority of these regions were affected in cognitively impaired subgroups in the broader literature. While the cognitively intact subgroup did not have significantly greater reductions than cognitively impaired patients in any circumstance, only the reductions in grey matter volume of the right angular gyrus (Shepherd et al., 2015) and cortical thickness of the right inferior temporal gyrus (Van Rheenen et al., 2018) were truly unique to cognitively intact patients across all studies. It is unlikely that these findings provide any evidence of true biological distinction in this subgroup, given the limited brain regions implicated. Instead, they may reflect the manifestation of individual differences in ‘cognitive reserve’; which has recently been empirically implicated in psychiatric disorders, and used to explain findings in in which more severe underlying brain pathology has been found in individuals with relatively minor clinical indicators of illness compared to those with much more severe symptoms (Leeson et al., 2011; Stern, 2012; Van Rheenen et al., 2019).

### Considerations and Future Directions

Through the synthesis of this review, a main challenge encountered was the high degree of methodological heterogeneity across all 20 studies. This heterogeneity broadly encompassed sample characteristics related to sample size, statistical methods used to define the subgroups, and also considerable variation in neuroimaging parameters, including the regions and indices of brain morphology under investigation, and brain atlases used to segment and label the brain. Indeed, several brain atlases, including the Desikan-Killiany, Destrieux or atlases otherwise unstated, were used across studies, which interfered to some extent in comparisons of regional effects across studies. Hence, the lack of consistency in findings across studies is not necessarily unexpected. Further, the sample sizes in some studies were relatively low, and different studies used different strengths of MRI scanner. Relevantly, some more recent studies used 3 Tesla scanners (Guimond et al., 2016; Poletti et al., 2014; Shepherd et al., 2015; Weinberg et al., 2016; Woodward & Heckers, 2015), which have greater spatial resolution and contrast detection than the 1.5 Tesla magnets used in the majority of studies. Notably, a 1.5 Tesla scanner was used in each study in which an absence of between-subgroup differences in brain morphology were observed (Alonso-Lana et al., 2016; Ayesa-Arriola et al., 2013; Colibazzi et al., 2013; Ortiz-Gil et al., 2011; Torres et al., 1997; Vaskinn et al., 2015; Wexler et al., 2009). It is possible that subtle between-subgroup differences were unable to be detected in these studies due to scanner strength rather than a true absence of differences. In light of these points, it is recommended that future studies on this topic strive for sufficient power and use higher resolution neuroimaging parameters.

Notably, a very limited number of studies examining recent onset SSD/BD and established BD samples, surface area or incorporating a longitudinal design were included in this review. This reflected a relative absence of studies of this type in the extant literature, rather than an inability of studies of this nature to meet our inclusion/exclusion criteria. Indeed, of the 20 studies examined, only two included surface area analyses (Van Rheenen et al., 2018; Vaskinn et al., 2015). Both of these studies focused on SSD samples and reported an absence of any differences between cognitive subgroups and healthy controls, as well as between-subgroup differences themselves. However, it is difficult to draw firm conclusions regarding the way in which surface area maps to different cognitive subgroups without an adequate number of studies in which it is measured. Thus, future research on this topic should endeavor to index this measure of brain morphology. Furthermore, the literature was predominantly focused on cross-sectional established SSD studies, with only two studies longitudinal in design (Ayesa-Arriola et al., 2013; Ho et al., 2020), one examined a recent-onset sample (Ayesa-Arriola et al., 2013), one examined a BD cohort explicitly (Alonso-Lana et al., 2016), and only two included BD patients alongside those with a SSD in cross-diagnostic analyses (Shepherd et al., 2015; Woodward & Heckers, 2015). Whilst this review did provide some preliminary evidence to suggest that cross-diagnostic SSD and BD studies mirror findings from studies of SSD explicitly, generalizing the findings to BD more generally is problematic without further research.

Also of note is that the conclusions drawn on the basis of the models in Fig. 1a-c relate to the presence or absence of statistically significant group differences and not the magnitude of effects. Few studies reported effects sizes for all comparisons irrespective of statistical significance, but it should be noted that inequality in the magnitude of effects could change the strength of evidence for a given pattern of findings. Similarly, conclusions drawn on the basis of certain patterns of findings assume the use of a healthy control sample. However, several studies did not include healthy controls in their analyses (Ayesa-Arriola et al., 2013; Poletti et al., 2014; Torres et al., 1997), or did not conduct analyses between healthy controls and certain subgroups for varying reasons (Alonso-Lana et al., 2016; Ortiz-Gil et al., 2011; Weinberg et al., 2016). Thus, the findings of these studies were unable to be well-integrated.

Moving forward, greater emphasis on BD and recent-onset studies, comprehensive analyses relative to healthy controls, those with a longitudinal design, and those explicitly reporting effect sizes for all comparisons will provide more insight into the association between brain morphology and cognition in SSD and BD. Further, as the scope of this review was limited to structural imaging analyses assessing brain volume, cortical thickness, and cortical surface area, future reviews of other aspects of brain morphology or brain function would be useful.

### Limitations

The findings of this review should be interpreted with the following limitations in mind. Only one author conducted title and abstract screening. Although this was a logistical issue rather than an issue by design, it is important to consider that screening by a single author may increase the risk of overlooking relevant studies to include in the review. The review was also not pre-registered, and the search was limited to two databases only. Although we intentionally included broad search terms and criteria to capture all of the relevant papers, it remains possible that some relevant studies were missed in the search process. Further, in the interests of being comprehensive, we also did not consider clinical and demographic factors in our eligibility criteria or results stratification, nor did we consider specific methodological approaches or measures used to define the cognitive subgroups. However, recent empirical evidence does suggest that cognitive subgroups of SSD and BD are not artefacts of the cognitive measures from which they arise (Karantonis et al., 2020). Nonetheless, caution is warranted in interpreting the findings of this review as the validity of inferences drawn from the compilation of cognitive subgroups defined by different cognitive measures is not established. Finally, studies examining social cognitive subgroups were excluded to achieve a focus solely on ‘cold’ cognition. Future research may wish to include studies pertaining to other facets such as emotion processing or theory of mind to develop a more holistic understanding of brain morphological characteristics related to ‘hot’ cognition.

### Conclusion

In summary, this review synthesized the findings from 20 available studies examining brain morphological characteristics of independent and cross-diagnostic samples of recent-onset and established SSD and BD. The majority of studies were focused on SSD samples and included measures of brain volume or thickness but not surface area. There was some evidence for widespread brain abnormalities associated with the presence of SSD or BD irrespective of cognitive impairment, including most consistently, in grey matter volume of the frontal lobe, inferior parietal lobe, and total brain and grey matter volume. In contrast, abnormalities in specific brain regions more strongly associated with cognitive impairment independent of SSD or BD disease presence were much more constrained. There was *some* evidence that cortical thickness was more strongly linked to cognitive function than disease state, and also *some* evidence of a severity grading effect, in which more severely cognitively impaired patients demonstrated a greater magnitude of brain structural abnormality compared to patients with better cognition. Taken together, the above findings do not provide strong evidence that cognitive subgroups of SSD or BD map to unique patterns of brain morphology. However, there is some preliminary evidence that cortical thickness may be more strongly tied to cognitive functioning, whilst volumetric deficits may be largely tied to the presence of disease.

## Supplementary Information

Below is the link to the electronic supplementary material.

Supplementary file1 (DOCX 248 KB)

## Data Availability

Data is available upon request.

## Code Availability

NA.
