# Correction to: Brain Morphological Characteristics of Cognitive Subgroups of Schizophrenia‑spectrum Disorders and Bipolar Disorder: a Systematic Review with Narrative Synthesis

**DOI:** 10.1007/s11065-022-09541-8

**Published:** 2022-03-28

**Authors:** James A. Karantonis, Sean P. Carruthers, Katherine E. Burdick, Christos Pantelis, Melissa Green, Susan L. Rossell, Matthew E. Hughes, Vanessa Cropley, Tamsyn E. Van Rheenen

**Affiliations:** 1 Melbourne Neuropsychiatry Centre, Level 3, Alan Gilbert, Building, 161 Barry St, Carlton, VIC 3053 Australia; 2 Faculty of Health, Arts and Design, School of Health Sciences, Centre for Mental Health, Swinburne University, Melbourne, Australia; 3 St Vincent’s Mental Health, St Vincent’s Hospital, Fitzroy, VIC Australia; 4 Florey Institute of Neuroscience and Mental Health, Parkville, Australia; 5 Department of Electrical and Electronic Engineering, University of Melbourne, Melbourne, VIC Australia; 6 Brigham and Women’s Hospital, Boston, MA USA; 7 Department of Psychiatry, Harvard Medical School, Boston, MA USA; 8 School of Psychiatry, University of New South Wales, (UNSW), Sydney, NSW Australia; 9 Neuroscience Research Australia, Randwick, NSW Australia

## Correction to: Neuropsychology Review https://doi.org/10.1007/s11065-021-09533-0

The original version of this article unfortunately contained a mistake in Fig. 1 caption. Below is the complete caption.

**Fig. 1** Three models to aid with data interpretation. Visual models of patterns of findings, **a**) findings likely to indicate brain morphological abnormalities more strongly associated with cognitive impairment, independent of SSD and/or BD disease presence; **b**) findings likely to indicate brain morphological abnormalities associated with SSD and/or BD disease presence, independent of cognitive impairment; **c**) findings likely to indicate brain morphological abnormalities reflecting the interaction of SSD and/or BD disease presence and cognitive impairment. Note ‘ ≠ ’ is indicative that there is a significant difference in brain morphology between (sub)groups; ‘ = ’ is indicative that there is no significant difference in brain morphology between (sub)groups. For brevity, the term ‘intact’ is used synonymously with ‘relatively intact’

The original article has been corrected.

