# Supplementary material for: Brain Morphological Characteristics of Cognitive Subgroups of Schizophrenia-Spectrum Disorders and Bipolar Disorder: A Systematic Review with Narrative Synthesis
Source: Neuropsychol Rev. 2022 Feb 22;33(1):192–220. doi: 10.1007/s11065-021-09533-0 (PMC9998576; doi:10.1007/s11065-021-09533-0)
Supplement: Supplementary file 1 — Supplementary file1 (DOCX 248 KB) [file 11065_2021_9533_MOESM1_ESM.docx]

**Brain morphological characteristics of cognitive subgroups of schizophrenia-spectrum disorders and bipolar disorder: a systematic review with narrative synthesis**

***Neuropsychology Review***

***Supplementary Material***

**Search terms**

Search terms were based on three concepts: diagnosis, cognition, and brain morphology. For diagnosis*,* the terms included: *schizophrenia, schizoaffective, schizophrenia spectrum, bipolar disorder, bipolar disorder, bipolar, manic, euthymic, bipolar depression, psychosis, first-episode psychosis, first episode/recent onset bipolar, first episode/recent onset schizophrenia* and *psychotic disorders.*

For cognition: *cognition, cognitive, cognition disorders, neurocognitive, neurocognition, neuropsychology, neuropsychological, cognitive deficit, cognitive function, cognitive ability, cognitive dysfunction, cognitive impairment, intelligence, IQ, intellect, memory, learning, attention, vigilance, speed of processing, executive, flexibility, reasoning, problem solving,* and *decision making*.

For brain morphology: *brain volume, brain surface area, brain thickness, brain structure, brain morphology, cortical volume, cortical surface area, cortical thickness, cortical structure, cortical morphology, grey matter, MRI, magnetic resonance, neuroimaging, voxel-based morphology,* and *VBM.*

**Search Strategy (PubMed database)**

("schizophrenia spectrum and other psychotic disorders"[MeSH Terms] OR "bipolar and related disorders"[MeSH Terms] OR schizophrenia[Title/Abstract] OR schizophrenic[Title/Abstract] OR bipolar[Title/Abstract] OR schizoaffective[Title/Abstract] OR “first episode psychosis”[Title/Abstract] OR “psychosis”[Title/Abstract] OR “first-episode psychosis”[Title/Abstract] OR “paediatric bipolar disorder” [Title/Abstract] OR “pediatric bipolar disorder” [Title/Abstract] OR “first episode bipolar disorder”[Title/Abstract] OR “first episode mania”[Title/Abstract] OR “recent onset bipolar disorder” [Title/Abstract])

AND

("cognition"[MeSH Terms] OR "cognition disorders"[MeSH Terms] OR "cognitive dysfunction"[MeSH Terms] OR cognit*[Title/Abstract] OR neuropsychol*[Title/Abstract] OR neurocognit*[Title/Abstract] OR “cognitive cluster”[Title/Abstract] OR “cognitive subgroup”[Title/Abstract] OR “cognitive subtype”[Title/Abstract] OR “cognitive heterogeneity”[Title/Abstract] OR “neuropsychological subgroup”[Title/Abstract] OR “neurocognitive subgroup”[Title/Abstract] OR “cluster analysis”[Title/Abstract] OR “clustering analyses”[Title/Abstract] OR intellectual[Title/Abstract] OR intelligence[Title/Abstract] OR memory[Title/Abstract] OR learning[Title/Abstract] OR attention[Title/Abstract] OR “speed of processing”[Title/Abstract] OR “processing speed”[Title/Abstract] OR “executive function”[Title/Abstract] OR “cognitive flexibility”[Title/Abstract] OR “problem solving”[Title/Abstract] OR “cognitive reserve”[Title/Abstract] OR “executive control”[Title/Abstract] OR recognition[Title/Abstract] OR priming[Title/Abstract] OR “inhibition”[Title/Abstract] OR “psychomotor speed”[Title/Abstract] OR “fluency”[Title/Abstract] OR “comprehension”[Title/Abstract] OR “IQ”[Title/Abstract] OR “WAIS”[Title/Abstract] OR “Weschler” OR “visuospatial”[Title/Abstract] OR “arithmetic”[Title/Abstract] OR ”motor functioning”[Title/Abstract] OR “executive dysfunction”[Title/Abstract] )

AND

((“cortical”[Title/Abstract] OR “brain”[Title/Abstract] OR “whole brain”[Title/Abstract] OR “whole-brain”[Title/Abstract] OR “cortex”[Title/Abstract] OR “grey matter”[Title/Abstract] OR “gray matter”[Title/Abstract] OR neocortical[Title/Abstract] OR neocortex[Title/Abstract] OR neuroanatomical[Title/Abstract] OR hippocampal[Title/Abstract] OR cerebral[Title/Abstract] OR hippocampus[Title/Abstract] OR cerebellum[Title/Abstract] OR cerebellar[Title/Abstract] OR “brain tissue”[Title/Abstract] or lobe[Title/Abstract] OR subcortical[Title/Abstract])

AND

(“thickness”[Title/Abstract] OR “surface area”[Title/Abstract] OR “volume”[Title/Abstract] OR “structure”[Title/Abstract] OR “structural”[Title/Abstract] OR “morphology”[Title/Abstract] OR “thinning”[Title/Abstract] OR “volumetric”[Title/Abstract] OR size[Title/Abstract] OR MRI[Title/Abstract] OR “magnetic resonance imag*”[Title/Abstract] OR gyrification[Title/Abstract] OR “voxel-based”[Title/Abstract] OR “mean curvature”[Title/Abstract] OR “freesurfer”[Title/Abstract] “abnormalities”[Title/Abstract] OR “intracranial volume”[Title/Abstract] OR “sulcal width”[Title/Abstract] OR size[Title/Abstract])

AND

("1990"[Date - Publication]: "3000"[Date - Publication]) NOT “systematic review”[Publication Type] NOT review[Publication Type]

**Search Strategy (Scopus database)**

TITLE-ABS ( schizophrenia OR schizophrenic OR schizoaffective OR psychosis OR paediatric AND bipolar AND disorder OR pediatric AND bipolar AND disorder OR first AND episode AND bipolar AND disorder OR bipolar OR first AND episode AND NOT rat AND mice ) AND TITLE-ABS ( cognit* OR neuropsychol* OR neurocognit* OR "cognitive cluster" OR "cognitive subgroup" OR "cognitive subtype" OR "cognitive heterogeneity" OR "neuropsychological subgroup" OR "neurocognitive subgroup" OR "cluster analysis" OR "clustering analyses" OR intellectual OR intelligence OR memory OR learning OR attention OR "speed of processing" OR "processing speed" OR "executive function" OR "cognitive flexibility" OR "problem solving" OR "cognitive reserve" OR "executive control" OR recognition OR priming OR inhibition OR "psychomotor speed" OR "fluency" OR comprehension OR iq OR visuospatial OR arithmetic OR "motor functioning" OR "executive dysfunction" ) AND ( TITLE-ABS ( "cortical" OR "brain" OR "whole brain" OR "whole-brain" OR "cortex" OR "grey matter" OR "gray matter" OR neocortical OR neocortex OR neuroanatomical OR hippocampal OR cerebral OR hippocampus OR cerebellum OR cerebellar OR lobe OR subcortical ) AND TITLE-ABS ( "thickness" OR "surface area" OR "volume" OR "structure" OR "structural" OR "morphology" OR "thinning" OR "volumetric" OR mri OR "magnetic resonance imag*" OR gyrification OR "voxel-based" OR "freesurfer" "abnormalities" OR "intracranial volume" ) ) AND DOCTYPE ( ar ) AND PUBYEAR > 1990

Supplementary Table 1. *Evaluation of study quality*

| Reference | Was statistical correction applied where appropriate? | Were automated methods for imaging analyses used? | Was a healthy comparison group included? | Were statistical comparisons made between all available (sub)groups? | Were the imaging parameters/protocol clearly described to enable replication? | Were the cognitive measures/protocol clearly described to enable replication? | Quality Check Score |
| --- | --- | --- | --- | --- | --- | --- | --- |
| (Alonso-Lana et al., 2016) | 1 | 1 | 1 | 0 | 1 | 1 | 5 |
| (Ayesa-Arriola et al., 2013) | 1 | 1 | 0 | 1 | 1 | 1 | 5 |
| (Cobia et al., 2011) | 1 | 1 | 1 | 1 | 1 | 1 | 6 |
| (Colibazzi et al., 2013) | 1 | 1 | 1 | 1 | 1 | 1 | 6 |
| (Czepielewski et al., 2017) | 1 | 1 | 1 | 1 | 1 | 1 | 6 |
| (Geisler et al., 2015) | 1 | 1 | 1 | 0 | 1 | 1 | 5 |
| (Gould et al., 2014) | 1 | 1 | 1 | 1 | 1 | 1 | 6 |
| (Guimond et al., 2016) | 1 | 1 | 1 | 1 | 1 | 1 | 6 |
| (Ho et al., 2020) | 1 | 1 | 1 | 1 | 1 | 1 | 6 |
| (Ortiz-Gil et al., 2011) | 1 | 1 | 1 | 0 | 1 | 1 | 5 |
| (Poletti et al., 2014) | 1 | 1 | 0 | 1 | 1 | 1 | 5 |
| (Rusch et al., 2007) | 1 | 1 | 1 | 1 | 1 | 1 | 6 |
| (Shepherd et al., 2015) | 1 | 1 | 1 | 1 | 1 | 1 | 6 |
| (Torres et al., 1997) | 1 | 0 | 0 | 1 | 1 | 1 | 4 |
| (Van Rheenen et al., 2018) | 1 | 1 | 1 | 1 | 1 | 1 | 6 |
| (Vaskinn et al., 2015) | 1 | 1 | 1 | 0 | 1 | 1 | 5 |
| (Weinberg et al., 2016) | 1 | 1 | 1 | 0 | 1 | 1 | 5 |
| (Wexler et al., 2009) | 1 | 1 | 1 | 1 | 1 | 1 | 6 |
| (Woodward & Heckers, 2015) | 1 | 1 | 1 | 1 | 1 | 1 | 6 |
| (Yasuda et al., 2020) | 1 | 1 | 1 | 0 | 1 | 1 | 5 |

Supplementary Table 2. *Cognitive subgroups classification*

| Number of Subgroups | Method | Author(s) |
| --- | --- | --- |
| 2 | *Cognitive cut-off scores* | (Alonso-Lana et al., 2016; Ayesa-Arriola et al., 2013; Colibazzi et al., 2013; Guimond et al., 2016; Ortiz-Gil et al., 2011; Poletti et al., 2014; Rusch et al., 2007; Shepherd et al., 2015; Torres et al., 1997; Wexler et al., 2009) |
| 2 | *Data-driven methods* | (Cobia et al., 2011; Gould et al., 2014) |
| 3 | *Cognitive cut-off scores* | (Czepielewski et al., 2017; Ho et al., 2020; Vaskinn et al., 2015; Woodward & Heckers, 2015; Yasuda et al., 2020) |
| 3 | *Data-driven methods* | (Van Rheenen et al., 2018) |
| 4 | *Data-driven methods* | (Geisler et al., 2015; Weinberg et al., 2016) |

Supplementary Table 3. *Paper findings, organized by lobe*

| Lobe | Region | Subgroup A: | had (a) ____: | in _____ relative to: | Subgroup B: | Cognition HC ≠ Impaired HC = Intact Impaired ≠ Intact | Disease HC ≠ Impaired HC ≠ Intact Impaired = Intact | Interaction HC ≠ Impaired HC ≠ Intact Impaired ≠ Intact | Reference |
| --- | --- | --- | --- | --- | --- | --- | --- | --- | --- |
| Cerebellum |  | Intact | No Difference | Grey Matter Volume | Healthy Controls | - | - | - | (Wexler et al., 2009) |
| Cerebellum |  | Impaired | No Difference | Grey Matter Volume | Healthy Controls | - | - | - | (Wexler et al., 2009) |
| Cerebellum |  | Compromised | Reduction | Grey Matter Volume | Healthy Controls | - | - | - | (Woodward and Heckers, 2015) |
| Cingulate Cortex | Anterior Cingulate | Impaired | Reduction | Cortical Thickness | Healthy Controls | - | - | - | (Cobia et al., 2011) |
| Cingulate Cortex | Anterior Cingulate | Preserved | No Difference | Grey Matter Volume | Healthy Controls | - | - | - | (Czepielewski et al., 2017) |
| Cingulate Cortex | Anterior Cingulate | Deteriorated | No Difference | Grey Matter Volume | Healthy Controls | - | - | - | (Czepielewski et al., 2017) |
| Cingulate Cortex | Anterior Cingulate | Compromised | No Difference | Grey Matter Volume | Healthy Controls | - | - | - | (Czepielewski et al., 2017) |
| Cingulate Cortex | Anterior Cingulate | Deteriorated | No Difference | Grey Matter Volume | Preserved | - | - | - | (Czepielewski et al., 2017) |
| Cingulate Cortex | Anterior Cingulate | Compromised | No Difference | Grey Matter Volume | Preserved | - | - | - | (Czepielewski et al., 2017) |
| Cingulate Cortex | Anterior Cingulate | Compromised | No Difference | Grey Matter Volume | Deteriorated | - | - | - | (Czepielewski et al., 2017) |
| Cingulate Cortex | Left Anterior Cingulate | Impaired | Reduction | Grey Matter Volume | Healthy Controls | - | - | - | (Shepherd et al., 2015) |
| Cingulate Cortex | Posterior Cingulate Gyrus | Intact | Reduction | Grey Matter Volume | Healthy Controls | - | - | - | (Colibazzi et al., 2013) |
| Cingulate Cortex | Rostral Anterior Cingulate | Severely Impaired | Reduction | Grey Matter Volume | Healthy Controls | - | - | - | (Weinberg et al., 2016) |
| Cingulate Cortex | Rostral Anterior Cingulate Cortex | Diminished Face Memory/Processing | Reduction | Cortical Thickness | Healthy Controls | - | - | - | (Geisler et al., 2015) |
| Frontal | Dorsolateral Prefrontal Cortex | Impaired | Reduction | White Matter Volume | Healthy Controls | Yes | - | - | (Wexler et al., 2009) |
| Frontal | Dorsolateral Prefrontal Cortex | Intact | No Difference | White Matter Volume | Healthy Controls | Yes | - | - | (Wexler et al., 2009) |
| Frontal | Dorsolateral Prefrontal Cortex | Impaired | Reduction | White Matter Volume | Intact | Yes | - | - | (Wexler et al., 2009) |
| Frontal | Frontal Pole | Preserved | Reduction | Grey Matter Volume | Healthy Controls | - | Yes | - | (Van Rheenen et al., 2018) |
| Frontal | Frontal Pole | Compromised | Reduction | Grey Matter Volume | Healthy Controls | - | Yes | - | (Van Rheenen et al., 2018) |
| Frontal | Frontal Pole | Deteriorated | Reduction | Grey Matter Volume | Healthy Controls | - | Yes | - | (Van Rheenen et al., 2018) |
| Frontal | Genu of the Corpus Callosum | Preserved | Reduction | White Matter Volume | Healthy Controls | - | - | - | (Woodward and Heckers, 2015) |
| Frontal | Inferior Frontal Gyrus | Impaired | Reduction | Cortical Thickness | Healthy Controls | - | - | - | (Cobia et al., 2011) |
| Frontal | Inferior Frontal Gyrus | Impaired | Reduction | Grey Matter Volume | Healthy Controls | - | - | - | (Colibazzi et al., 2013) |
| Frontal | Inferior Frontal Gyrus | Impaired | Reduction | Grey Matter Volume | Intact | - | - | - | (Poletti et al., 2014) |
| Frontal | Lateral Orbital Gyrus | Impaired | Reduction | Cortical Thickness | Healthy Controls | - | - | - | (Cobia et al., 2011) |
| Frontal | Lateral Orbitofrontal | Severely Impaired | Reduction | Grey Matter Volume | Healthy Controls | - | - | - | (Weinberg et al., 2016) |
| Frontal | Lateral Orbitofrontal Gyrus | Compromised | Reduction | Grey Matter Volume | Preserved | - | - | - | (Van Rheenen et al., 2018) |
| Frontal | Lateral Orbitofrontal Gyrus | Compromised | Reduction | Grey Matter Volume | Deteriorated | - | - | - | (Van Rheenen et al., 2018) |
| Frontal | Left Dorsolateral Prefrontal Cortex | Intact | Reduction | Grey Matter Volume | Healthy Controls | - | - | Yes | (Rusch et al., 2007) |
| Frontal | Left Dorsolateral Prefrontal Cortex | Impaired | Reduction | Grey Matter Volume | Healthy Controls | - | - | Yes | (Rusch et al., 2007) |
| Frontal | Left Dorsolateral Prefrontal Cortex | Impaired | Reduction | Grey Matter Volume | Intact | - | - | Yes | (Rusch et al., 2007) |
| Frontal | Left Frontal Lobe | Impaired | Reduction | Cortical Thickness | Healthy Controls | - | Yes | - | (Cobia et al., 2011) |
| Frontal | Left Frontal Lobe | Intact | Reduction | Cortical Thickness | Healthy Controls | - | Yes | - | (Cobia et al., 2011) |
| Frontal | Left Frontal Lobe | Impaired | Reduction | Cortical Thickness | Healthy Controls | Yes | - | - | (Guimond et al., 2016) |
| Frontal | Left Frontal Lobe | Impaired | Reduction | Cortical Thickness | Intact | Yes | - | - | (Guimond et al., 2016) |
| Frontal | Left Inferior Frontal Cortex | Intact | Reduction | White Matter Volume | Healthy Controls | - | - | - | (Alonso-Lana et al., 2016) |
| Frontal | Left Lateral Orbitofrontal Gyrus | Compromised | Reduction | Grey Matter Volume | Healthy Controls | - | - | - | (Van Rheenen et al., 2018) |
| Frontal | Left Lateral Orbitofrontal Gyrus | Deteriorated | Reduction | Grey Matter Volume | Healthy Controls | - | - | - | (Van Rheenen et al., 2018) |
| Frontal | Left Lateral Orbitofrontal Gyrus | Preserved | Reduction | Cortical Thickness | Healthy Controls | - | Yes | - | (Ho et al., 2020) |
| Frontal | Left Lateral Orbitofrontal Gyrus | Deteriorated | Reduction | Cortical Thickness | Healthy Controls | - | Yes | - | (Ho et al., 2020) |
| Frontal | Left Lateral Orbitofrontal Gyrus | Compromised | Reduction | Cortical Thickness | Healthy Controls | - | Yes | - | (Ho et al., 2020) |
| Frontal | Left Lateral Orbitofrontal Gyrus | Deteriorated | No Difference | Cortical Thickness | Preserved | - | Yes | - | (Ho et al., 2020) |
| Frontal | Left Lateral Orbitofrontal Gyrus | Compromised | No Difference | Cortical Thickness | Preserved | - | Yes | - | (Ho et al., 2020) |
| Frontal | Left Lateral Orbitofrontal Gyrus | Compromised | No Difference | Cortical Thickness | Deteriorated | - | Yes | - | (Ho et al., 2020) |
| Frontal | Left Medial Frontal Gyrus | Impaired | Reduction | Grey Matter Volume | Healthy Controls | - | Yes | - | (Shepherd et al., 2015) |
| Frontal | Left Medial Frontal Gyrus | Intact | Reduction | Grey Matter Volume | Healthy Controls | - | Yes | - | (Shepherd et al., 2015) |
| Frontal | Left Medial Orbital Frontal Gyrus | Preserved | Reduction | Cortical Thickness | Healthy Controls | - | Yes | - | (Ho et al., 2020) |
| Frontal | Left Medial Orbital Frontal Gyrus | Deteriorated | Reduction | Cortical Thickness | Healthy Controls | - | Yes | - | (Ho et al., 2020) |
| Frontal | Left Medial Orbital Frontal Gyrus | Compromised | No Difference | Cortical Thickness | Healthy Controls | - | Yes | - | (Ho et al., 2020) |
| Frontal | Left Medial Orbital Frontal Gyrus | Deteriorated | No Difference | Cortical Thickness | Preserved | - | Yes | - | (Ho et al., 2020) |
| Frontal | Left Medial Orbital Frontal Gyrus | Compromised | No Difference | Cortical Thickness | Preserved | - | Yes | - | (Ho et al., 2020) |
| Frontal | Left Medial Orbital Frontal Gyrus | Compromised | No Difference | Cortical Thickness | Deteriorated | - | Yes | - | (Ho et al., 2020) |
| Frontal | Left Middle Frontal Gyrus | Impaired | Reduction | Cortical Thickness | Healthy Controls | Yes | - | - | (Guimond et al., 2016) |
| Frontal | Left Middle Frontal Gyrus | Impaired | Reduction | Cortical Thickness | Intact | Yes | - | - | (Guimond et al., 2016) |
| Frontal | Left Orbitofrontal Gyrus | Impaired | Reduction | Cortical Thickness | Healthy Controls | Yes | - | - | (Guimond et al., 2016) |
| Frontal | Left Orbitofrontal Gyrus | Impaired | Reduction | Cortical Thickness | Intact | Yes | - | - | (Guimond et al., 2016) |
| Frontal | Left Pars Opercularis | Preserved | Reduction | Cortical Thickness | Healthy Controls | - | Yes | - | (Ho et al., 2020) |
| Frontal | Left Pars Opercularis | Deteriorated | Reduction | Cortical Thickness | Healthy Controls | - | Yes | - | (Ho et al., 2020) |
| Frontal | Left Pars Opercularis | Compromised | No Difference | Cortical Thickness | Healthy Controls | - | Yes | - | (Ho et al., 2020) |
| Frontal | Left Pars Opercularis | Deteriorated | No Difference | Cortical Thickness | Preserved | - | Yes | - | (Ho et al., 2020) |
| Frontal | Left Pars Opercularis | Compromised | No Difference | Cortical Thickness | Preserved | - | Yes | - | (Ho et al., 2020) |
| Frontal | Left Pars Opercularis | Compromised | No Difference | Cortical Thickness | Deteriorated | - | Yes | - | (Ho et al., 2020) |
| Frontal | Left Pars Orbitalis | Preserved | Reduction | Cortical Thickness | Healthy Controls | - | Yes | - | (Ho et al., 2020) |
| Frontal | Left Pars Orbitalis | Deteriorated | Reduction | Cortical Thickness | Healthy Controls | - | Yes | - | (Ho et al., 2020) |
| Frontal | Left Pars Orbitalis | Compromised | Reduction | Cortical Thickness | Healthy Controls | - | Yes | - | (Ho et al., 2020) |
| Frontal | Left Pars Orbitalis | Deteriorated | No Difference | Cortical Thickness | Preserved | - | Yes | - | (Ho et al., 2020) |
| Frontal | Left Pars Orbitalis | Compromised | No Difference | Cortical Thickness | Preserved | - | Yes | - | (Ho et al., 2020) |
| Frontal | Left Pars Orbitalis | Compromised | No Difference | Cortical Thickness | Deteriorated | - | Yes | - | (Ho et al., 2020) |
| Frontal | Left Pars Orbitalis | Deteriorated | Reduction | Grey Matter Volume | Healthy Controls | Yes | - | - | (Yasuda et al., 2020) |
| Frontal | Left Pars Orbitalis | Preserved | No Difference | Grey Matter Volume | Healthy Controls | Yes | - | - | (Yasuda et al., 2020) |
| Frontal | Left Pars Orbitalis | Deteriorated | Reduction | Grey Matter Volume | Preserved | Yes | - | - | (Yasuda et al., 2020) |
| Frontal | Left Pars Triangularis | Preserved | Reduction | Cortical Thickness | Healthy Controls | - | Yes | - | (Ho et al., 2020) |
| Frontal | Left Pars Triangularis | Deteriorated | Reduction | Cortical Thickness | Healthy Controls | - | Yes | - | (Ho et al., 2020) |
| Frontal | Left Pars Triangularis | Compromised | Reduction | Cortical Thickness | Healthy Controls | - | Yes | - | (Ho et al., 2020) |
| Frontal | Left Pars Triangularis | Deteriorated | No Difference | Cortical Thickness | Preserved | - | Yes | - | (Ho et al., 2020) |
| Frontal | Left Pars Triangularis | Compromised | No Difference | Cortical Thickness | Preserved | - | Yes | - | (Ho et al., 2020) |
| Frontal | Left Pars Triangularis | Compromised | No Difference | Cortical Thickness | Deteriorated | - | Yes | - | (Ho et al., 2020) |
| Frontal | Left Pars Triangularis | Deteriorated | Reduction | Grey Matter Volume | Healthy Controls | - | - | - | (Yasuda et al., 2020) |
| Frontal | Left Pars Triangularis | Preserved | No Difference | Grey Matter Volume | Healthy Controls | - | - | - | (Yasuda et al., 2020) |
| Frontal | Left Pars Triangularis | Deteriorated | No Difference | Grey Matter Volume | Preserved | - | - | - | (Yasuda et al., 2020) |
| Frontal | Left Precentral Gyrus | Impaired | Reduction | Cortical Thickness | Healthy Controls | - | - | Yes | (Guimond et al., 2016) |
| Frontal | Left Precentral Gyrus | Impaired | Reduction | Cortical Thickness | Intact | - | - | Yes | (Guimond et al., 2016) |
| Frontal | Left Precentral Gyrus | Intact | Reduction | Cortical Thickness | Healthy Controls | - | - | Yes | (Guimond et al., 2016) |
| Frontal | Left Precentral Gyrus | Deteriorated | Reduction | Grey Matter Volume | Healthy Controls | - | Yes | - | (Yasuda et al., 2020) |
| Frontal | Left Precentral Gyrus | Preserved | Reduction | Grey Matter Volume | Healthy Controls | - | Yes | - | (Yasuda et al., 2020) |
| Frontal | Left Precentral Gyrus | Deteriorated | No Difference | Grey Matter Volume | Preserved | - | Yes | - | (Yasuda et al., 2020) |
| Frontal | Left Rostral Middle Frontal Gyrus | Deteriorated | Reduction | Grey Matter Volume | Healthy Controls | - | Yes | - | (Yasuda et al., 2020) |
| Frontal | Left Rostral Middle Frontal Gyrus | Preserved | Reduction | Grey Matter Volume | Healthy Controls | - | Yes | - | (Yasuda et al., 2020) |
| Frontal | Left Rostral Middle Frontal Gyrus | Deteriorated | No Difference | Grey Matter Volume | Preserved | - | Yes | - | (Yasuda et al., 2020) |
| Frontal | Left Superior Frontal | Diminished Face Memory/Processing | Reduction | Cortical Thickness | Healthy Controls | - | - | - | (Geisler et al., 2015) |
| Frontal | Left Superior Frontal Gyrus | Intact | Reduction | Grey Matter Volume | Healthy Controls | - | Yes | - | (Shepherd et al., 2015) |
| Frontal | Left Superior Frontal Gyrus | Impaired | Reduction | Grey Matter Volume | Healthy Controls | - | Yes | - | (Shepherd et al., 2015) |
| Frontal | Left Superior Frontal Gyrus | Compromised | Reduction | Grey Matter Volume | Healthy Controls | - | - | - | (Van Rheenen et al., 2018) |
| Frontal | Left Superior Frontal Gyrus | Preserved | No Difference | Cortical Thickness | Healthy Controls | - | - | - | (Ho et al., 2020) |
| Frontal | Left Superior Frontal Gyrus | Deteriorated | Reduction | Cortical Thickness | Healthy Controls | - | - | - | (Ho et al., 2020) |
| Frontal | Left Superior Frontal Gyrus | Compromised | No Difference | Cortical Thickness | Healthy Controls | - | - | - | (Ho et al., 2020) |
| Frontal | Left Superior Frontal Gyrus | Deteriorated | No Difference | Cortical Thickness | Preserved | - | - | - | (Ho et al., 2020) |
| Frontal | Left Superior Frontal Gyrus | Compromised | No Difference | Cortical Thickness | Preserved | - | - | - | (Ho et al., 2020) |
| Frontal | Left Superior Frontal Gyrus | Compromised | No Difference | Cortical Thickness | Deteriorated | - | - | - | (Ho et al., 2020) |
| Frontal | Left Superior Frontal Gyrus | Deteriorated | Reduction | Grey Matter Volume | Healthy Controls | - | Yes | - | (Yasuda et al., 2020) |
| Frontal | Left Superior Frontal Gyrus | Preserved | Reduction | Grey Matter Volume | Healthy Controls | - | Yes | - | (Yasuda et al., 2020) |
| Frontal | Left Superior Frontal Gyrus | Deteriorated | No Difference | Grey Matter Volume | Preserved | - | Yes | - | (Yasuda et al., 2020) |
| Frontal | Medial Ortbital Frontal | Severely Impaired | Reduction | Grey Matter Volume | Healthy Controls | - | - | - | (Weinberg et al., 2016) |
| Frontal | Middle Frontal Gyrus | Impaired | Reduction | Grey Matter Volume | Healthy Controls | - | Yes | - | (Colibazzi et al., 2013) |
| Frontal | Middle Frontal Gyrus | Intact | Reduction | Grey Matter Volume | Healthy Controls | - | Yes | - | (Colibazzi et al., 2013) |
| Frontal | Middle Frontal Gyrus | Preserved | No Difference | Grey Matter Volume | Healthy Controls | - | - | - | (Czepielewski et al., 2017) |
| Frontal | Middle Frontal Gyrus | Deteriorated | No Difference | Grey Matter Volume | Healthy Controls | - | - | - | (Czepielewski et al., 2017) |
| Frontal | Middle Frontal Gyrus | Compromised | No Difference | Grey Matter Volume | Healthy Controls | - | - | - | (Czepielewski et al., 2017) |
| Frontal | Middle Frontal Gyrus | Deteriorated | No Difference | Grey Matter Volume | Preserved | - | - | - | (Czepielewski et al., 2017) |
| Frontal | Middle Frontal Gyrus | Compromised | No Difference | Grey Matter Volume | Preserved | - | - | - | (Czepielewski et al., 2017) |
| Frontal | Middle Frontal Gyrus | Compromised | No Difference | Grey Matter Volume | Deteriorated | - | - | - | (Czepielewski et al., 2017) |
| Frontal | Orbital/Medial Prefrontal Cortex to the anterior cingulate | Intact | Reduction | Grey Matter Volume | Healthy Controls | - | - | - | (Ortiz-Gil et al., 2011) |
| Frontal | Orbitofrontal | Impaired | Reduction | White Matter Volume | Healthy Controls | Yes | - | - | (Wexler et al., 2009) |
| Frontal | Orbitofrontal | Intact | No Difference | White Matter Volume | Healthy Controls | Yes | - | - | (Wexler et al., 2009) |
| Frontal | Orbitofrontal | Impaired | Reduction | White Matter Volume | Intact | Yes | - | - | (Wexler et al., 2009) |
| Frontal | Orbitofrontal Cortex | Intact | Reduction | Grey Matter Volume | Healthy Controls | - | - | - | (Wexler et al., 2009) |
| Frontal | Orbitofrontal Cortex | Impaired | No Difference | Grey Matter Volume | Healthy Controls | - | - | - | (Wexler et al., 2009) |
| Frontal | Pars Opercularis | Severely Impaired | Reduction | Grey Matter Volume | Healthy Controls | - | - | - | (Weinberg et al., 2016) |
| Frontal | Pars Orbitalis | Impaired | Reduction | Cortical Thickness | Healthy Controls | - | - | - | (Cobia et al., 2011) |
| Frontal | Pars Orbitalis | Preserved | Reduction | Grey Matter Volume | Healthy Controls | - | Yes | - | (Van Rheenen et al., 2018) |
| Frontal | Pars Orbitalis | Compromised | Reduction | Grey Matter Volume | Healthy Controls | - | Yes | - | (Van Rheenen et al., 2018) |
| Frontal | Pars Orbitalis | Deteriorated | Reduction | Grey Matter Volume | Healthy Controls | - | Yes | - | (Van Rheenen et al., 2018) |
| Frontal | Pars Triangularis | Compromised | Reduction | Grey Matter Volume | Healthy Controls | - | - | - | (Van Rheenen et al., 2018) |
| Frontal | Posterior Superior Frontal | Impaired | Reduction | Cortical Thickness | Healthy Controls | - | - | - | (Cobia et al., 2011) |
| Frontal | Precentral Gyrus | Impaired | Reduction | Grey Matter Volume | Healthy Controls | - | - | - | (Colibazzi et al., 2013) |
| Frontal | Precentral Gyrus | Deteriorated | Reduction | Grey Matter Volume | Healthy Controls | - | - | - | (Woodward and Heckers, 2015) |
| Frontal | Premotor Cortex | Impaired | Reduction | White Matter Volume | Healthy Controls | Yes | - | - | (Wexler et al., 2009) |
| Frontal | Premotor Cortex | Intact | No Difference | White Matter Volume | Healthy Controls | Yes | - | - | (Wexler et al., 2009) |
| Frontal | Premotor Cortex | Impaired | Reduction | White Matter Volume | Intact | Yes | - | - | (Wexler et al., 2009) |
| Frontal | Right Frontal Lobe | Impaired | Reduction | Cortical Thickness | Healthy Controls | - | Yes | - | (Cobia et al., 2011) |
| Frontal | Right Frontal Lobe | Intact | Reduction | Cortical Thickness | Healthy Controls | - | Yes | - | (Cobia et al., 2011) |
| Frontal | Right Lateral Orbitofrontal Cortex | Deteriorated | Reduction | Grey Matter Volume | Healthy Controls | - | - | - | (Yasuda et al., 2020) |
| Frontal | Right Lateral Orbitofrontal Cortex | Preserved | No Difference | Grey Matter Volume | Healthy Controls | - | - | - | (Yasuda et al., 2020) |
| Frontal | Right Lateral Orbitofrontal Cortex | Deteriorated | No Difference | Grey Matter Volume | Preserved | - | - | - | (Yasuda et al., 2020) |
| Frontal | Right Lateral Orbitofrontal Gyrus | Compromised | Reduction | Grey Matter Volume | Healthy Controls | - | - | - | (Van Rheenen et al., 2018) |
| Frontal | Right Lateral Orbitofrontal Gyrus | Preserved | No Difference | Cortical Thickness | Healthy Controls | - | - | - | (Ho et al., 2020) |
| Frontal | Right Lateral Orbitofrontal Gyrus | Deteriorated | Reduction | Cortical Thickness | Healthy Controls | - | - | - | (Ho et al., 2020) |
| Frontal | Right Lateral Orbitofrontal Gyrus | Compromised | Reduction | Cortical Thickness | Healthy Controls | - | - | - | (Ho et al., 2020) |
| Frontal | Right Lateral Orbitofrontal Gyrus | Deteriorated | No Difference | Cortical Thickness | Preserved | - | - | - | (Ho et al., 2020) |
| Frontal | Right Lateral Orbitofrontal Gyrus | Compromised | No Difference | Cortical Thickness | Preserved | - | - | - | (Ho et al., 2020) |
| Frontal | Right Lateral Orbitofrontal Gyrus | Compromised | No Difference | Cortical Thickness | Deteriorated | - | - | - | (Ho et al., 2020) |
| Frontal | Right Medial Orbital Frontal Gyrus | Preserved | No Difference | Cortical Thickness | Healthy Controls | - | - | - | (Ho et al., 2020) |
| Frontal | Right Medial Orbital Frontal Gyrus | Deteriorated | Reduction | Cortical Thickness | Healthy Controls | - | - | - | (Ho et al., 2020) |
| Frontal | Right Medial Orbital Frontal Gyrus | Compromised | No Difference | Cortical Thickness | Healthy Controls | - | - | - | (Ho et al., 2020) |
| Frontal | Right Medial Orbital Frontal Gyrus | Deteriorated | No Difference | Cortical Thickness | Preserved | - | - | - | (Ho et al., 2020) |
| Frontal | Right Medial Orbital Frontal Gyrus | Compromised | No Difference | Cortical Thickness | Preserved | - | - | - | (Ho et al., 2020) |
| Frontal | Right Medial Orbital Frontal Gyrus | Compromised | No Difference | Cortical Thickness | Deteriorated | - | - | - | (Ho et al., 2020) |
| Frontal | Right Medial Orbitofrontal Cortex | Deteriorated | Reduction | Grey Matter Volume | Healthy Controls | - | - | - | (Yasuda et al., 2020) |
| Frontal | Right Medial Orbitofrontal Cortex | Preserved | No Difference | Grey Matter Volume | Healthy Controls | - | - | - | (Yasuda et al., 2020) |
| Frontal | Right Medial Orbitofrontal Cortex | Deteriorated | No Difference | Grey Matter Volume | Preserved | - | - | - | (Yasuda et al., 2020) |
| Frontal | Right Medial Orbitofrontal Gyrus | Compromised | Reduction | Grey Matter Volume | Healthy Controls | - | - | - | (Van Rheenen et al., 2018) |
| Frontal | Right Opercular Gyrus | Impaired | Reduction | Grey Matter Volume | Healthy Controls | - | - | - | (Shepherd et al., 2015) |
| Frontal | Right Pars Opercularis | Preserved | Reduction | Cortical Thickness | Healthy Controls | - | Yes | - | (Ho et al., 2020) |
| Frontal | Right Pars Opercularis | Deteriorated | Reduction | Cortical Thickness | Healthy Controls | - | Yes | - | (Ho et al., 2020) |
| Frontal | Right Pars Opercularis | Compromised | No Difference | Cortical Thickness | Healthy Controls | - | Yes | - | (Ho et al., 2020) |
| Frontal | Right Pars Opercularis | Deteriorated | No Difference | Cortical Thickness | Preserved | - | Yes | - | (Ho et al., 2020) |
| Frontal | Right Pars Opercularis | Compromised | No Difference | Cortical Thickness | Preserved | - | Yes | - | (Ho et al., 2020) |
| Frontal | Right Pars Opercularis | Compromised | No Difference | Cortical Thickness | Deteriorated | - | Yes | - | (Ho et al., 2020) |
| Frontal | Right Pars Orbitalis | Preserved | Reduction | Cortical Thickness | Healthy Controls | - | Yes | - | (Ho et al., 2020) |
| Frontal | Right Pars Orbitalis | Deteriorated | Reduction | Cortical Thickness | Healthy Controls | - | Yes | - | (Ho et al., 2020) |
| Frontal | Right Pars Orbitalis | Compromised | Reduction | Cortical Thickness | Healthy Controls | - | Yes | - | (Ho et al., 2020) |
| Frontal | Right Pars Orbitalis | Deteriorated | No Difference | Cortical Thickness | Preserved | - | Yes | - | (Ho et al., 2020) |
| Frontal | Right Pars Orbitalis | Compromised | No Difference | Cortical Thickness | Preserved | - | Yes | - | (Ho et al., 2020) |
| Frontal | Right Pars Orbitalis | Compromised | No Difference | Cortical Thickness | Deteriorated | - | Yes | - | (Ho et al., 2020) |
| Frontal | Right Pars Triangularis | Compromised | Reduction | Grey Matter Volume | Preserved | - | - | - | (Van Rheenen et al., 2018) |
| Frontal | Right Pars Triangularis | Compromised | Reduction | Grey Matter Volume | Deteriorated | - | - | - | (Van Rheenen et al., 2018) |
| Frontal | Right Pars Triangularis | Preserved | Reduction | Cortical Thickness | Healthy Controls | - | Yes | - | (Ho et al., 2020) |
| Frontal | Right Pars Triangularis | Deteriorated | Reduction | Cortical Thickness | Healthy Controls | - | Yes | - | (Ho et al., 2020) |
| Frontal | Right Pars Triangularis | Compromised | No Difference | Cortical Thickness | Healthy Controls | - | Yes | - | (Ho et al., 2020) |
| Frontal | Right Pars Triangularis | Deteriorated | No Difference | Cortical Thickness | Preserved | - | Yes | - | (Ho et al., 2020) |
| Frontal | Right Pars Triangularis | Compromised | No Difference | Cortical Thickness | Preserved | - | Yes | - | (Ho et al., 2020) |
| Frontal | Right Pars Triangularis | Compromised | No Difference | Cortical Thickness | Deteriorated | - | Yes | - | (Ho et al., 2020) |
| Frontal | Right Pars Triangularis | Deteriorated | Reduction | Grey Matter Volume | Healthy Controls | Yes | - | - | (Yasuda et al., 2020) |
| Frontal | Right Pars Triangularis | Preserved | No Difference | Grey Matter Volume | Healthy Controls | Yes | - | - | (Yasuda et al., 2020) |
| Frontal | Right Pars Triangularis | Deteriorated | Reduction | Grey Matter Volume | Preserved | Yes | - | - | (Yasuda et al., 2020) |
| Frontal | Right Precentral Gyrus | Intact | Reduction | Grey Matter Volume | Healthy Controls | - | - | - | (Alonso-Lana et al., 2016) |
| Frontal | Right Precentral Gyrus | Diminished Intellectual Functioning | Reduction | Cortical Thickness | Diminished Verbal Fluency | - | - | - | (Geisler et al., 2015) |
| Frontal | Right Precentral Gyrus | Intact | Reduction | Cortical Thickness | Healthy Controls | - | - | - | (Guimond et al., 2016) |
| Frontal | Right Precentral Gyrus | Impaired | Reduction | Grey Matter Volume | Intact | - | - | - | (Shepherd et al., 2015) |
| Frontal | Right Precentral Gyrus | Compromised | Reduction | Grey Matter Volume | Healthy Controls | - | - | - | (Van Rheenen et al., 2018) |
| Frontal | Right Precentral Gyrus | Deteriorated | Reduction | Grey Matter Volume | Healthy Controls | - | - | - | (Van Rheenen et al., 2018) |
| Frontal | Right Rolandic Gyrus | Impaired | Reduction | Grey Matter Volume | Healthy Controls | - | - | - | (Shepherd et al., 2015) |
| Frontal | Right Rolandic Operculum | Impaired | Reduction | Grey Matter Volume | Intact | - | - | - | (Shepherd et al., 2015) |
| Frontal | Right Rostral Middle Frontal Gyrus | Preserved | Reduction | Cortical Thickness | Healthy Controls | - | Yes | - | (Ho et al., 2020) |
| Frontal | Right Rostral Middle Frontal Gyrus | Deteriorated | Reduction | Cortical Thickness | Healthy Controls | - | Yes | - | (Ho et al., 2020) |
| Frontal | Right Rostral Middle Frontal Gyrus | Compromised | No Difference | Cortical Thickness | Healthy Controls | - | Yes | - | (Ho et al., 2020) |
| Frontal | Right Rostral Middle Frontal Gyrus | Deteriorated | No Difference | Cortical Thickness | Preserved | - | Yes | - | (Ho et al., 2020) |
| Frontal | Right Rostral Middle Frontal Gyrus | Compromised | No Difference | Cortical Thickness | Preserved | - | Yes | - | (Ho et al., 2020) |
| Frontal | Right Rostral Middle Frontal Gyrus | Compromised | No Difference | Cortical Thickness | Deteriorated | - | Yes | - | (Ho et al., 2020) |
| Frontal | Right Superior Frontal Gyrus | Preserved | No Difference | Cortical Thickness | Healthy Controls | - | - | - | (Ho et al., 2020) |
| Frontal | Right Superior Frontal Gyrus | Deteriorated | Reduction | Cortical Thickness | Healthy Controls | - | - | - | (Ho et al., 2020) |
| Frontal | Right Superior Frontal Gyrus | Compromised | No Difference | Cortical Thickness | Healthy Controls | - | - | - | (Ho et al., 2020) |
| Frontal | Right Superior Frontal Gyrus | Deteriorated | No Difference | Cortical Thickness | Preserved | - | - | - | (Ho et al., 2020) |
| Frontal | Right Superior Frontal Gyrus | Compromised | No Difference | Cortical Thickness | Preserved | - | - | - | (Ho et al., 2020) |
| Frontal | Right Superior Frontal Gyrus | Compromised | No Difference | Cortical Thickness | Deteriorated | - | - | - | (Ho et al., 2020) |
| Frontal | Right Superior Frontal Gyrus | Deteriorated | Reduction | Grey Matter Volume | Healthy Controls | - | Yes | - | (Yasuda et al., 2020) |
| Frontal | Right Superior Frontal Gyrus | Preserved | Reduction | Grey Matter Volume | Healthy Controls | - | Yes | - | (Yasuda et al., 2020) |
| Frontal | Right Superior Frontal Gyrus | Deteriorated | No Difference | Grey Matter Volume | Preserved | - | Yes | - | (Yasuda et al., 2020) |
| Frontal | Rostral Middle Frontal Cortex | Compromised | Reduction | Grey Matter Volume | Healthy Controls | - | - | - | (Van Rheenen et al., 2018) |
| Frontal | Sensorimotor Cortex | Impaired | Reduction | Cortical Thickness | Healthy Controls | - | - | - | (Cobia et al., 2011) |
| Frontal | Sensorimotor Cortex | Impaired | Reduction | White Matter Volume | Healthy Controls | Yes | - | - | (Wexler et al., 2009) |
| Frontal | Sensorimotor Cortex | Intact | No Difference | White Matter Volume | Healthy Controls | Yes | - | - | (Wexler et al., 2009) |
| Frontal | Sensorimotor Cortex | Impaired | Reduction | White Matter Volume | Intact | Yes | - | - | (Wexler et al., 2009) |
| Frontal | Subgenual | Impaired | Reduction | White Matter Volume | Healthy Controls | Yes | - | - | (Wexler et al., 2009) |
| Frontal | Subgenual | Intact | No Difference | White Matter Volume | Healthy Controls | Yes | - | - | (Wexler et al., 2009) |
| Frontal | Subgenual | Impaired | Reduction | White Matter Volume | Intact | Yes | - | - | (Wexler et al., 2009) |
| Frontal | Superior Frontal Cortex | Compromised | Reduction | Grey Matter Volume | Deteriorated | - | - | - | (Van Rheenen et al., 2018) |
| Frontal | Superior Frontal Cortex | Severely Impaired | Reduction | Grey Matter Volume | Healthy Controls | - | - | - | (Weinberg et al., 2016) |
| Frontal | Superior Frontal Gyrus | Intact | Reduction | Cortical Thickness | Healthy Controls | - | Yes | - | (Colibazzi et al., 2013) |
| Frontal | Superior Frontal Gyrus | Impaired | Reduction | Cortical Thickness | Healthy Controls | - | Yes | - | (Colibazzi et al., 2013) |
| Frontal | Superior/Middle Frontal Gyrus | Preserved | Reduction | White Matter Volume | Healthy Controls | - | - | - | (Woodward and Heckers, 2015) |
| Insula Cortex | Anterior Insula | Preserved | No Difference | Grey Matter Volume | Healthy Controls | Yes | - | - | (Czepielewski et al., 2017) |
| Insula Cortex | Anterior Insula | Deteriorated | Reduction | Grey Matter Volume | Healthy Controls | Yes | - | - | (Czepielewski et al., 2017) |
| Insula Cortex | Anterior Insula | Compromised | Reduction | Grey Matter Volume | Healthy Controls | Yes | - | - | (Czepielewski et al., 2017) |
| Insula Cortex | Anterior Insula | Deteriorated | No Difference | Grey Matter Volume | Preserved | Yes | - | - | (Czepielewski et al., 2017) |
| Insula Cortex | Anterior Insula | Compromised | Reduction | Grey Matter Volume | Preserved | Yes | - | - | (Czepielewski et al., 2017) |
| Insula Cortex | Anterior Insula | Compromised | No Difference | Grey Matter Volume | Deteriorated | Yes | - | - | (Czepielewski et al., 2017) |
| Insula Cortex | Insula | Impaired | Reduction | Cortical Thickness | Healthy Controls | - | - | - | (Cobia et al., 2011) |
| Insula Cortex | Insula | Severely Impaired | Reduction | Grey Matter Volume | Healthy Controls | - | - | - | (Weinberg et al., 2016) |
| Insula Cortex | Insula | Severely Impaired | Reduction | Grey Matter Volume | Moderately Impaired | - | - | - | (Weinberg et al., 2016) |
| Insula Cortex | Left Insula | Deteriorated | Reduction | Grey Matter Volume | Healthy Controls | Yes | - | - | (Yasuda et al., 2020) |
| Insula Cortex | Left Insula | Preserved | No Difference | Grey Matter Volume | Healthy Controls | Yes | - | - | (Yasuda et al., 2020) |
| Insula Cortex | Left Insula | Deteriorated | Reduction | Grey Matter Volume | Preserved | Yes | - | - | (Yasuda et al., 2020) |
| Occipital | Cuneus | Impaired | Reduction | Cortical Thickness | Healthy Controls | - | - | - | (Cobia et al., 2011) |
| Occipital | Lateral Occipital Cortex | Impaired | Reduction | Cortical Thickness | Healthy Controls | - | - | - | (Cobia et al., 2011) |
| Occipital | Left Cuneus | Preserved | Reduction | Cortical Thickness | Healthy Controls | - | - | - | (Ho et al., 2020) |
| Occipital | Left Cuneus | Deteriorated | No Difference | Cortical Thickness | Healthy Controls | - | - | - | (Ho et al., 2020) |
| Occipital | Left Cuneus | Compromised | No Difference | Cortical Thickness | Healthy Controls | - | - | - | (Ho et al., 2020) |
| Occipital | Left Cuneus | Deteriorated | No Difference | Cortical Thickness | Preserved | - | - | - | (Ho et al., 2020) |
| Occipital | Left Cuneus | Compromised | No Difference | Cortical Thickness | Preserved | - | - | - | (Ho et al., 2020) |
| Occipital | Left Cuneus | Compromised | No Difference | Cortical Thickness | Deteriorated | - | - | - | (Ho et al., 2020) |
| Occipital | Left Lingual Gyrus | Preserved | Reduction | Cortical Thickness | Healthy Controls | - | Yes | - | (Ho et al., 2020) |
| Occipital | Left Lingual Gyrus | Deteriorated | Reduction | Cortical Thickness | Healthy Controls | - | Yes | - | (Ho et al., 2020) |
| Occipital | Left Lingual Gyrus | Compromised | Reduction | Cortical Thickness | Healthy Controls | - | Yes | - | (Ho et al., 2020) |
| Occipital | Left Lingual Gyrus | Deteriorated | No Difference | Cortical Thickness | Preserved | - | Yes | - | (Ho et al., 2020) |
| Occipital | Left Lingual Gyrus | Compromised | No Difference | Cortical Thickness | Preserved | - | Yes | - | (Ho et al., 2020) |
| Occipital | Left Lingual Gyrus | Compromised | No Difference | Cortical Thickness | Deteriorated | - | Yes | - | (Ho et al., 2020) |
| Occipital | Left Lingual Gyrus | Deteriorated | Reduction | Grey Matter Volume | Healthy Controls | - | - | - | (Yasuda et al., 2020) |
| Occipital | Left Lingual Gyrus | Preserved | No Difference | Grey Matter Volume | Healthy Controls | - | - | - | (Yasuda et al., 2020) |
| Occipital | Left Lingual Gyrus | Deteriorated | No Difference | Grey Matter Volume | Preserved | - | - | - | (Yasuda et al., 2020) |
| Occipital | Left Occipital Gyrus | Preserved | Reduction | Cortical Thickness | Healthy Controls | - | Yes | - | (Ho et al., 2020) |
| Occipital | Left Occipital Gyrus | Deteriorated | Reduction | Cortical Thickness | Healthy Controls | - | Yes | - | (Ho et al., 2020) |
| Occipital | Left Occipital Gyrus | Compromised | No Difference | Cortical Thickness | Healthy Controls | - | Yes | - | (Ho et al., 2020) |
| Occipital | Left Occipital Gyrus | Deteriorated | No Difference | Cortical Thickness | Preserved | - | Yes | - | (Ho et al., 2020) |
| Occipital | Left Occipital Gyrus | Compromised | No Difference | Cortical Thickness | Preserved | - | Yes | - | (Ho et al., 2020) |
| Occipital | Left Occipital Gyrus | Compromised | No Difference | Cortical Thickness | Deteriorated | - | Yes | - | (Ho et al., 2020) |
| Occipital | Left Occipital Lobe | Impaired | Reduction | Cortical Thickness | Healthy Controls | Yes | - | - | (Cobia et al., 2011) |
| Occipital | Left Occipital Lobe | Impaired | Reduction | Cortical Thickness | Healthy Controls | Yes | - | - | (Cobia et al., 2011) |
| Occipital | Lingual Gyrus | Diminished Face Memory/Processing | Reduction | Cortical Thickness | Healthy Controls | - | - | - | (Geisler et al., 2015) |
| Occipital | Lingual Gyrus | Severely Impaired | Reduction | Grey Matter Volume | Healthy Controls | Yes | - | - | (Weinberg et al., 2016) |
| Occipital | Lingual Gyrus | Severely Impaired | Reduction | Grey Matter Volume | Preserved | Yes | - | - | (Weinberg et al., 2016) |
| Occipital | Lingual Gyrus | Severely Impaired | Reduction | Grey Matter Volume | Moderately Impaired | Yes | - | - | (Weinberg et al., 2016) |
| Occipital | Parietal-Occipital | Impaired | Reduction | White Matter Volume | Healthy Controls | Yes | - | - | (Wexler et al., 2009) |
| Occipital | Parietal-Occipital | Intact | No Difference | White Matter Volume | Healthy Controls | Yes | - | - | (Wexler et al., 2009) |
| Occipital | Parietal-Occipital | Impaired | Reduction | White Matter Volume | Intact | Yes | - | - | (Wexler et al., 2009) |
| Occipital | Right Cuneus | Preserved | No Difference | Cortical Thickness | Healthy Controls | - | - | - | (Ho et al., 2020) |
| Occipital | Right Cuneus | Deteriorated | Reduction | Cortical Thickness | Healthy Controls | - | - | - | (Ho et al., 2020) |
| Occipital | Right Cuneus | Compromised | No Difference | Cortical Thickness | Healthy Controls | - | - | - | (Ho et al., 2020) |
| Occipital | Right Cuneus | Deteriorated | No Difference | Cortical Thickness | Preserved | - | - | - | (Ho et al., 2020) |
| Occipital | Right Cuneus | Compromised | No Difference | Cortical Thickness | Preserved | - | - | - | (Ho et al., 2020) |
| Occipital | Right Cuneus | Compromised | No Difference | Cortical Thickness | Deteriorated | - | - | - | (Ho et al., 2020) |
| Occipital | Right Lateral Occipital Gyrus | Compromised | Reduction | Grey Matter Volume | Healthy Controls | - | - | - | (Van Rheenen et al., 2018) |
| Occipital | Right Lingual Gyrus | Preserved | No Difference | Cortical Thickness | Healthy Controls | - | - | - | (Ho et al., 2020) |
| Occipital | Right Lingual Gyrus | Deteriorated | Reduction | Cortical Thickness | Healthy Controls | - | - | - | (Ho et al., 2020) |
| Occipital | Right Lingual Gyrus | Compromised | No Difference | Cortical Thickness | Healthy Controls | - | - | - | (Ho et al., 2020) |
| Occipital | Right Lingual Gyrus | Deteriorated | No Difference | Cortical Thickness | Preserved | - | - | - | (Ho et al., 2020) |
| Occipital | Right Lingual Gyrus | Compromised | No Difference | Cortical Thickness | Preserved | - | - | - | (Ho et al., 2020) |
| Occipital | Right Lingual Gyrus | Compromised | No Difference | Cortical Thickness | Deteriorated | - | - | - | (Ho et al., 2020) |
| Occipital | Right Lingual Gyrus | Deteriorated | Reduction | Grey Matter Volume | Healthy Controls | - | - | - | (Yasuda et al., 2020) |
| Occipital | Right Lingual Gyrus | Preserved | No Difference | Grey Matter Volume | Healthy Controls | - | - | - | (Yasuda et al., 2020) |
| Occipital | Right Lingual Gyrus | Deteriorated | No Difference | Grey Matter Volume | Preserved | - | - | - | (Yasuda et al., 2020) |
| Occipital | Right Occipital Gyris | Compromised | Reduction | Grey Matter Volume | Deteriorated | - | - | - | (Van Rheenen et al., 2018) |
| Occipital | Right Occipital Gyrus | Preserved | Reduction | Cortical Thickness | Healthy Controls | - | Yes | - | (Ho et al., 2020) |
| Occipital | Right Occipital Gyrus | Deteriorated | Reduction | Cortical Thickness | Healthy Controls | - | Yes | - | (Ho et al., 2020) |
| Occipital | Right Occipital Gyrus | Compromised | No Difference | Cortical Thickness | Healthy Controls | - | Yes | - | (Ho et al., 2020) |
| Occipital | Right Occipital Gyrus | Deteriorated | No Difference | Cortical Thickness | Preserved | - | Yes | - | (Ho et al., 2020) |
| Occipital | Right Occipital Gyrus | Compromised | No Difference | Cortical Thickness | Preserved | - | Yes | - | (Ho et al., 2020) |
| Occipital | Right Occipital Gyrus | Compromised | No Difference | Cortical Thickness | Deteriorated | - | Yes | - | (Ho et al., 2020) |
| Occipital | Right Occipital Lobe | Impaired | Reduction | Cortical Thickness | Healthy Controls | Yes | - | - | (Cobia et al., 2011) |
| Occipital | Right Occipital Lobe | Impaired | Reduction | Cortical Thickness | Intact | Yes | - | - | (Cobia et al., 2011) |
| Occipital |  | Diminished Face Memory/Processing | Reduction | Cortical Thickness | Healthy Controls | - | - | - | (Geisler et al., 2015) |
| Parietal | Inferior Parietal | Preserved | Reduction | Grey Matter Volume | Healthy Controls | - | Yes | - | (Weinberg et al., 2016) |
| Parietal | Inferior Parietal | Moderately Impaired | Reduction | Grey Matter Volume | Healthy Controls | - | Yes | - | (Weinberg et al., 2016) |
| Parietal | Inferior Parietal | Severely Impaired | Reduction | Grey Matter Volume | Healthy Controls | - | Yes | - | (Weinberg et al., 2016) |
| Parietal | Left Inferior Parietal Cortex | Preserved | Reduction | Grey Matter Volume | Healthy Controls | - | Yes | - | (Van Rheenen et al., 2018) |
| Parietal | Left Inferior Parietal Cortex | Compromised | Reduction | Grey Matter Volume | Healthy Controls | - | Yes | - | (Van Rheenen et al., 2018) |
| Parietal | Left Inferior Parietal Cortex | Deteriorated | Reduction | Grey Matter Volume | Healthy Controls | - | Yes | - | (Van Rheenen et al., 2018) |
| Parietal | Left Inferior Parietal Cortex | Preserved | Reduction | Cortical Thickness | Healthy Controls | - | Yes | - | (Ho et al., 2020) |
| Parietal | Left Inferior Parietal Cortex | Deteriorated | Reduction | Cortical Thickness | Healthy Controls | - | Yes | - | (Ho et al., 2020) |
| Parietal | Left Inferior Parietal Cortex | Compromised | No Difference | Cortical Thickness | Healthy Controls | - | Yes | - | (Ho et al., 2020) |
| Parietal | Left Inferior Parietal Cortex | Deteriorated | No Difference | Cortical Thickness | Preserved | - | Yes | - | (Ho et al., 2020) |
| Parietal | Left Inferior Parietal Cortex | Compromised | No Difference | Cortical Thickness | Preserved | - | Yes | - | (Ho et al., 2020) |
| Parietal | Left Inferior Parietal Cortex | Compromised | No Difference | Cortical Thickness | Deteriorated | - | Yes | - | (Ho et al., 2020) |
| Parietal | Left Medial Parietal Lobe | Preserved | Reduction | White Matter Volume | Healthy Controls | - | - | - | (Woodward and Heckers, 2015) |
| Parietal | Left Parietal Lobe | Impaired | Reduction | Cortical Thickness | Healthy Controls | - | - | - | (Cobia et al., 2011) |
| Parietal | Paracentral Gyrus | Impaired | Reduction | Cortical Thickness | Healthy Controls | - | - | - | (Cobia et al., 2011) |
| Parietal | Parietal Lobe | Impaired | Reduction | Grey Matter Volume | Intact | - | - | - | (Ayesa-Arriola et al., 2013) |
| Parietal | Postcentral Gyrus | Impaired | Reduction | Grey Matter Volume | Healthy Controls | - | - | - | (Colibazzi et al., 2013) |
| Parietal | Precuneus | Severely Impaired | Reduction | Grey Matter Volume | Healthy Controls | - | - | - | (Weinberg et al., 2016) |
| Parietal | Right Angular Gyrus | Intact | Reduction | Grey Matter Volume | Healthy Controls | - | - | - | (Shepherd et al., 2015) |
| Parietal | Right Inferior Parietal | Deteriorated | Reduction | Grey Matter Volume | Healthy Controls | - | - | - | (Yasuda et al., 2020) |
| Parietal | Right Inferior Parietal | Preserved | No Difference | Grey Matter Volume | Healthy Controls | - | - | - | (Yasuda et al., 2020) |
| Parietal | Right Inferior Parietal | Deteriorated | No Difference | Grey Matter Volume | Preserved | - | - | - | (Yasuda et al., 2020) |
| Parietal | Right Parietal Lobe | Impaired | Reduction | Cortical Thickness | Healthy Controls | Yes | - | - | (Cobia et al., 2011) |
| Parietal | Right Parietal Lobe | Impaired | Reduction | Cortical Thickness | Intact | Yes | - | - | (Cobia et al., 2011) |
| Parietal | Right Postcentral Gyrus | Impaired | Reduction | Grey Matter Volume | Intact | - | - | - | (Shepherd et al., 2015) |
| Parietal | Right Precuneus | Intact | Reduction | Grey Matter Volume | Healthy Controls | - | - | - | (Shepherd et al., 2015) |
| Parietal | Right Supramarginal Gyrus | Preserved | Reduction | Grey Matter Volume | Healthy Controls | - | Yes | - | (Van Rheenen et al., 2018) |
| Parietal | Right Supramarginal Gyrus | Compromised | Reduction | Grey Matter Volume | Healthy Controls | - | Yes | - | (Van Rheenen et al., 2018) |
| Parietal | Right Supramarginal Gyrus | Deteriorated | Reduction | Grey Matter Volume | Healthy Controls | - | Yes | - | (Van Rheenen et al., 2018) |
| Parietal | Superior Parietal Cortex | Impaired | Reduction | Cortical Thickness | Healthy Controls | - | - | - | (Cobia et al., 2011) |
| Parietal | Supramarginal Gyrus | Impaired | Reduction | Cortical Thickness | Healthy Controls | - | - | - | (Cobia et al., 2011) |
| Parietal | Supramarginal Gyrus | Impaired | Reduction | Grey Matter Volume | Healthy Controls | - | - | - | (Colibazzi et al., 2013) |
| Parietal | Supramarginal Gyrus | Diminished Verbal Fluency | Reduction | Cortical Thickness | Healthy Controls | - | - | - | (Geisler et al., 2015) |
| Perisylvian Cortex |  | Intact | Reduction | Cortical Thickness | Healthy Controls | - | Yes | - | (Colibazzi et al., 2013) |
| Perisylvian Cortex |  | Impaired | Reduction | Cortical Thickness | Healthy Controls | - | Yes | - | (Colibazzi et al., 2013) |
| Perisylvian Cortex |  | Impaired | Reduction | White Matter Volume | Intact | - | - | - | (Colibazzi et al., 2013) |
| Subcortical | Amygdala | Intact | Reduction | Grey Matter Volume | Healthy Controls | - | - | - | (Wexler et al., 2009) |
| Subcortical | Amygdala | Impaired | No Difference | Grey Matter Volume | Healthy Controls | - | - | - | (Wexler et al., 2009) |
| Subcortical | Lateral Ventricles | Severely Impaired | Reduction | Grey Matter Volume | Healthy Controls | - | - | - | (Weinberg et al., 2016) |
| Subcortical | Left Amygdala | Preserved | No Difference | Grey Matter Volume | Healthy Controls | Yes | - | - | (Ho et al., 2020) |
| Subcortical | Left Amygdala | Deteriorated | No Difference | Grey Matter Volume | Healthy Controls | Yes | - | - | (Ho et al., 2020) |
| Subcortical | Left Amygdala | Compromised | Reduction | Grey Matter Volume | Healthy Controls | Yes | - | - | (Ho et al., 2020) |
| Subcortical | Left Amygdala | Deteriorated | No Difference | Grey Matter Volume | Preserved | Yes | - | - | (Ho et al., 2020) |
| Subcortical | Left Amygdala | Compromised | Reduction | Grey Matter Volume | Preserved | Yes | - | - | (Ho et al., 2020) |
| Subcortical | Left Amygdala | Compromised | No Difference | Grey Matter Volume | Deteriorated | Yes | - | - | (Ho et al., 2020) |
| Subcortical | Left Pallidum | Deteriorated | Increase | Grey Matter Volume | Healthy Controls | - | Yes | - | (Yasuda et al., 2020) |
| Subcortical | Left Pallidum | Preserved | Increase | Grey Matter Volume | Healthy Controls | - | Yes | - | (Yasuda et al., 2020) |
| Subcortical | Left Pallidum | Deteriorated | No Difference | Grey Matter Volume | Preserved | - | Yes | - | (Yasuda et al., 2020) |
| Subcortical | Left Thalamus | Preserved | No Difference | Grey Matter Volume | Healthy Controls | - | - | - | (Ho et al., 2020) |
| Subcortical | Left Thalamus | Deteriorated | Reduction | Grey Matter Volume | Healthy Controls | - | - | - | (Ho et al., 2020) |
| Subcortical | Left Thalamus | Compromised | No Difference | Grey Matter Volume | Healthy Controls | - | - | - | (Ho et al., 2020) |
| Subcortical | Left Thalamus | Deteriorated | No Difference | Grey Matter Volume | Preserved | - | - | - | (Ho et al., 2020) |
| Subcortical | Left Thalamus | Compromised | No Difference | Grey Matter Volume | Preserved | - | - | - | (Ho et al., 2020) |
| Subcortical | Left Thalamus | Compromised | No Difference | Grey Matter Volume | Deteriorated | - | - | - | (Ho et al., 2020) |
| Subcortical | Pallidum | Preserved | Increase | Grey Matter Volume | Healthy Controls | - | Yes | - | (Van Rheenen et al., 2018) |
| Subcortical | Pallidum | Compromised | Increase | Grey Matter Volume | Healthy Controls | - | Yes | - | (Van Rheenen et al., 2018) |
| Subcortical | Pallidum | Deteriorated | Increase | Grey Matter Volume | Healthy Controls | - | Yes | - | (Van Rheenen et al., 2018) |
| Subcortical | Putamen | Preserved | Increase | Grey Matter Volume | Healthy Controls | - | Yes | - | (Van Rheenen et al., 2018) |
| Subcortical | Putamen | Compromised | Increase | Grey Matter Volume | Healthy Controls | - | Yes | - | (Van Rheenen et al., 2018) |
| Subcortical | Putamen | Deteriorated | Increase | Grey Matter Volume | Healthy Controls | - | Yes | - | (Van Rheenen et al., 2018) |
| Subcortical | Right Pallidum | Deteriorated | Increase | Grey Matter Volume | Healthy Controls | - | - | - | (Yasuda et al., 2020) |
| Subcortical | Right Pallidum | Preserved | No Difference | Grey Matter Volume | Healthy Controls | - | - | - | (Yasuda et al., 2020) |
| Subcortical | Right Pallidum | Deteriorated | No Difference | Grey Matter Volume | Preserved | - | - | - | (Yasuda et al., 2020) |
| Subcortical | Right Putamen | Deteriorated | Increase | Grey Matter Volume | Healthy Controls | - | Yes | - | (Yasuda et al., 2020) |
| Subcortical | Right Putamen | Preserved | Increase | Grey Matter Volume | Healthy Controls | - | Yes | - | (Yasuda et al., 2020) |
| Subcortical | Right Putamen | Deteriorated | No Difference | Grey Matter Volume | Preserved | - | Yes | - | (Yasuda et al., 2020) |
| Subcortical | Right Thalamus | Preserved | Reduction | Grey Matter Volume | Healthy Controls | - | Yes | - | (Ho et al., 2020) |
| Subcortical | Right Thalamus | Deteriorated | Reduction | Grey Matter Volume | Healthy Controls | - | Yes | - | (Ho et al., 2020) |
| Subcortical | Right Thalamus | Compromised | No Difference | Grey Matter Volume | Healthy Controls | - | Yes | - | (Ho et al., 2020) |
| Subcortical | Right Thalamus | Deteriorated | No Difference | Grey Matter Volume | Preserved | - | Yes | - | (Ho et al., 2020) |
| Subcortical | Right Thalamus | Compromised | No Difference | Grey Matter Volume | Preserved | - | Yes | - | (Ho et al., 2020) |
| Subcortical | Right Thalamus | Compromised | No Difference | Grey Matter Volume | Deteriorated | - | Yes | - | (Ho et al., 2020) |
| Subcortical | Thalamus | Intact | No Difference | Grey Matter Volume | Healthy Controls | - | - | - | (Wexler et al., 2009) |
| Subcortical | Thalamus | Impaired | Reduction | Grey Matter Volume | Healthy Controls | - | - | - | (Wexler et al., 2009) |
| Subcortical | Thalamus | Compromised | Reduction | Grey Matter Volume | Healthy Controls | - | - | - | (Woodward and Heckers, 2015) |
| Subcortical | Thalamus | Deteriorated | Reduction | Grey Matter Volume | Healthy Controls | - | - | - | (Woodward and Heckers, 2015) |
| Subcortical | Thalamus/Cerebellum/Pons | Impaired | No Difference | Grey Matter Volume | Intact | - | - | - | (Rusch et al., 2007) |
| Subcortical | Thalamus/Cerebellum/Pons | Impaired | No Difference | Grey Matter Volume | Healthy Controls | - | - | - | (Rusch et al., 2007) |
| Subcortical | Thalamus/Cerebellum/Pons | Intact | No Difference | Grey Matter Volume | Healthy Controls | - | - | - | (Rusch et al., 2007) |
| Subcortical |  | Intellectually Normal | No Difference | Grey Matter Volume | Healthy Controls | - | - | - | (Vaskinn et al., 2015) |
| Subcortical |  | Intellectually Superior | No Difference | Grey Matter Volume | Healthy Controls | - | - | - | (Vaskinn et al., 2015) |
| Subcortical |  | Intellectually Superior | No Difference | Grey Matter Volume | Intellectually Normal | - | - | - | (Vaskinn et al., 2015) |
| Subcortical |  | Preserved | No Difference | Grey Matter Volume | Healthy Controls | - | - | - | (Weinberg et al., 2016) |
| Subcortical |  | Moderately Impaired | No Difference | Grey Matter Volume | Healthy Controls | - | - | - | (Weinberg et al., 2016) |
| Subcortical |  | Severely Impaired | No Difference | Grey Matter Volume | Healthy Controls | - | - | - | (Weinberg et al., 2016) |
| Subcortical |  | Moderately Impaired | No Difference | Grey Matter Volume | Preserved | - | - | - | (Weinberg et al., 2016) |
| Subcortical |  | Severely Impaired | No Difference | Grey Matter Volume | Preserved | - | - | - | (Weinberg et al., 2016) |
| Subcortical |  | Severely Impaired | No Difference | Grey Matter Volume | Moderately Impaired | - | - | - | (Weinberg et al., 2016) |
| Temporal | Banks of the Superior Temporal Sulcus | Severely Impaired | Reduction | Grey Matter Volume | Healthy Controls | Yes | - | - | (Weinberg et al., 2016) |
| Temporal | Banks of the Superior Temporal Sulcus | Severely Impaired | Reduction | Grey Matter Volume | Preserved | Yes | - | - | (Weinberg et al., 2016) |
| Temporal | Entorhinal | Severely Impaired | Reduction | Grey Matter Volume | Healthy Controls | - | - | - | (Weinberg et al., 2016) |
| Temporal | Fusiform Gyrus | Impaired | Reduction | Cortical Thickness | Healthy Controls | - | - | - | (Cobia et al., 2011) |
| Temporal | Fusiform Gyrus | Severely Impaired | Reduction | Grey Matter Volume | Healthy Controls | - | - | - | (Weinberg et al., 2016) |
| Temporal | Hippocampus | Preserved | No Difference | Grey Matter Volume | Healthy Controls | - | - | - | (Czepielewski et al., 2017) |
| Temporal | Hippocampus | Deteriorated | No Difference | Grey Matter Volume | Healthy Controls | - | - | - | (Czepielewski et al., 2017) |
| Temporal | Hippocampus | Compromised | No Difference | Grey Matter Volume | Healthy Controls | - | - | - | (Czepielewski et al., 2017) |
| Temporal | Hippocampus | Deteriorated | No Difference | Grey Matter Volume | Preserved | - | - | - | (Czepielewski et al., 2017) |
| Temporal | Hippocampus | Compromised | No Difference | Grey Matter Volume | Preserved | - | - | - | (Czepielewski et al., 2017) |
| Temporal | Hippocampus | Compromised | No Difference | Grey Matter Volume | Deteriorated | - | - | - | (Czepielewski et al., 2017) |
| Temporal | Hippocampus | Intact | Reduction | Cortical Thickness | Healthy Controls | - | - | Yes | (Guimond et al., 2016) |
| Temporal | Hippocampus | Impaired | Reduction | Cortical Thickness | Healthy Controls | - | - | Yes | (Guimond et al., 2016) |
| Temporal | Hippocampus | Impaired | Reduction | Cortical Thickness | Intact | - | - | Yes | (Guimond et al., 2016) |
| Temporal | Hippocampus | Impaired | Reduction | Grey Matter Volume | Healthy Controls | - | - | - | (Shepherd et al., 2015) |
| Temporal | Hippocampus | Preserved | Reduction | Grey Matter Volume | Healthy Controls | - | Yes | - | (Van Rheenen et al., 2018) |
| Temporal | Hippocampus | Compromised | Reduction | Grey Matter Volume | Healthy Controls | - | Yes | - | (Van Rheenen et al., 2018) |
| Temporal | Hippocampus | Deteriorated | Reduction | Grey Matter Volume | Healthy Controls | - | Yes | - | (Van Rheenen et al., 2018) |
| Temporal | Hippocampus | Compromised | Reduction | Grey Matter Volume | Deteriorated | - | Yes | - | (Van Rheenen et al., 2018) |
| Temporal | Hippocampus | Severely Impaired | Reduction | Grey Matter Volume | Healthy Controls | Yes | - | - | (Weinberg et al., 2016) |
| Temporal | Hippocampus | Severely Impaired | Reduction | Grey Matter Volume | Preserved | Yes | - | - | (Weinberg et al., 2016) |
| Temporal | Hippocampus | Intact | No Difference | Grey Matter Volume | Healthy Controls | - | - | - | (Wexler et al., 2009) |
| Temporal | Hippocampus | Impaired | Reduction | Grey Matter Volume | Healthy Controls | - | - | - | (Wexler et al., 2009) |
| Temporal | Inferior Temporal | Severely Impaired | Reduction | Grey Matter Volume | Healthy Controls | - | - | - | (Weinberg et al., 2016) |
| Temporal | Left Banks of the Superior Temporal Sulcus | Preserved | Reduction | Cortical Thickness | Healthy Controls | - | Yes | - | (Ho et al., 2020) |
| Temporal | Left Banks of the Superior Temporal Sulcus | Deteriorated | Reduction | Cortical Thickness | Healthy Controls | - | Yes | - | (Ho et al., 2020) |
| Temporal | Left Banks of the Superior Temporal Sulcus | Compromised | No Difference | Cortical Thickness | Healthy Controls | - | Yes | - | (Ho et al., 2020) |
| Temporal | Left Banks of the Superior Temporal Sulcus | Deteriorated | No Difference | Cortical Thickness | Preserved | - | Yes | - | (Ho et al., 2020) |
| Temporal | Left Banks of the Superior Temporal Sulcus | Compromised | No Difference | Cortical Thickness | Preserved | - | Yes | - | (Ho et al., 2020) |
| Temporal | Left Banks of the Superior Temporal Sulcus | Compromised | No Difference | Cortical Thickness | Deteriorated | - | Yes | - | (Ho et al., 2020) |
| Temporal | Left Fusiform Gyrus | Deteriorated | Reduction | Grey Matter Volume | Healthy Controls | - | - | - | (Yasuda et al., 2020) |
| Temporal | Left Fusiform Gyrus | Preserved | No Difference | Grey Matter Volume | Healthy Controls | - | - | - | (Yasuda et al., 2020) |
| Temporal | Left Fusiform Gyrus | Deteriorated | No Difference | Grey Matter Volume | Preserved | - | - | - | (Yasuda et al., 2020) |
| Temporal | Left Hippocampus | Preserved | No Difference | Grey Matter Volume | Healthy Controls | Yes | - | - | (Ho et al., 2020) |
| Temporal | Left Hippocampus | Deteriorated | Reduction | Grey Matter Volume | Healthy Controls | Yes | - | - | (Ho et al., 2020) |
| Temporal | Left Hippocampus | Compromised | No Difference | Grey Matter Volume | Healthy Controls | Yes | - | - | (Ho et al., 2020) |
| Temporal | Left Hippocampus | Deteriorated | Reduction | Grey Matter Volume | Preserved | Yes | - | - | (Ho et al., 2020) |
| Temporal | Left Hippocampus | Compromised | No Difference | Grey Matter Volume | Preserved | Yes | - | - | (Ho et al., 2020) |
| Temporal | Left Hippocampus | Compromised | No Difference | Grey Matter Volume | Deteriorated | Yes | - | - | (Ho et al., 2020) |
| Temporal | Left Hippocampus | Deteriorated | Reduction | Grey Matter Volume | Healthy Controls | - | Yes | - | (Yasuda et al., 2020) |
| Temporal | Left Hippocampus | Preserved | Reduction | Grey Matter Volume | Healthy Controls | - | Yes | - | (Yasuda et al., 2020) |
| Temporal | Left Hippocampus | Deteriorated | No Difference | Grey Matter Volume | Preserved | - | Yes | - | (Yasuda et al., 2020) |
| Temporal | Left Inferior Temporal Gyrus | Compromised | Reduction | Grey Matter Volume | Healthy Controls | - | - | - | (Van Rheenen et al., 2018) |
| Temporal | Left Parahippocampal Gyrus | Compromised | Reduction | Grey Matter Volume | Healthy Controls | - | - | - | (Van Rheenen et al., 2018) |
| Temporal | Left Parahippocampal Gyrus | Deteriorated | Reduction | Grey Matter Volume | Healthy Controls | - | - | - | (Yasuda et al., 2020) |
| Temporal | Left Parahippocampal Gyrus | Preserved | No Difference | Grey Matter Volume | Healthy Controls | - | - | - | (Yasuda et al., 2020) |
| Temporal | Left Parahippocampal Gyrus | Deteriorated | No Difference | Grey Matter Volume | Preserved | - | - | - | (Yasuda et al., 2020) |
| Temporal | Left Superior Temporal Gyrus | Intact | Reduction | Grey Matter Volume | Healthy Controls | - | - | - | (Van Rheenen et al., 2018) |
| Temporal | Left Superior Temporal Gyrus | Compromised | Reduction | Grey Matter Volume | Healthy Controls | - | - | - | (Woodward and Heckers, 2015) |
| Temporal | Left Superior Temporal Gyrus | Deteriorated | Reduction | Grey Matter Volume | Healthy Controls | Yes | - | - | (Yasuda et al., 2020) |
| Temporal | Left Superior Temporal Gyrus | Preserved | No Difference | Grey Matter Volume | Healthy Controls | Yes | - | - | (Yasuda et al., 2020) |
| Temporal | Left Superior Temporal Gyrus | Deteriorated | Reduction | Grey Matter Volume | Preserved | Yes | - | - | (Yasuda et al., 2020) |
| Temporal | Left Temporal Lobe | Impaired | Reduction | Cortical Thickness | Healthy Controls | - | - | Yes | (Cobia et al., 2011) |
| Temporal | Left Temporal Lobe | Intact | Reduction | Cortical Thickness | Healthy Controls | - | - | Yes | (Cobia et al., 2011) |
| Temporal | Left Temporal Lobe | Impaired | Reduction | Cortical Thickness | Intact | - | - | Yes | (Cobia et al., 2011) |
| Temporal | Left Temporal Pole | Compromised | Reduction | Grey Matter Volume | Healthy Controls | - | - | - | (Van Rheenen et al., 2018) |
| Temporal | Medial Temporal Lobe | Compromised | Reduction | Grey Matter Volume | Healthy Controls | - | - | - | (Woodward and Heckers, 2015) |
| Temporal | Medial Temporal Lobe | Deteriorated | Reduction | Grey Matter Volume | Healthy Controls | - | - | - | (Woodward and Heckers, 2015) |
| Temporal | Middle Temporal Gyrus | Impaired | Reduction | Cortical Thickness | Healthy Controls | - | - | - | (Cobia et al., 2011) |
| Temporal | Middle Temporal Gyrus | Impaired | Reduction | Grey Matter Volume | Healthy Controls | - | - | - | (Colibazzi et al., 2013) |
| Temporal | Middle Temporal Gyrus | Diminished Face Memory/Processing | Reduction | Cortical Thickness | Healthy Controls | - | - | - | (Geisler et al., 2015) |
| Temporal | Middle Temporal Gyrus | Preserved | Reduction | Grey Matter Volume | Healthy Controls | - | Yes | - | (Van Rheenen et al., 2018) |
| Temporal | Middle Temporal Gyrus | Compromised | Reduction | Grey Matter Volume | Healthy Controls | - | Yes | - | (Van Rheenen et al., 2018) |
| Temporal | Middle Temporal Gyrus | Deteriorated | Reduction | Grey Matter Volume | Healthy Controls | - | Yes | - | (Van Rheenen et al., 2018) |
| Temporal | Middle Temporal Gyrus | Severely Impaired | Reduction | Grey Matter Volume | Healthy Controls | - | - | - | (Weinberg et al., 2016) |
| Temporal | Parahippocampal Gyrus | Impaired | Reduction | Cortical Thickness | Healthy Controls | - | - | - | (Cobia et al., 2011) |
| Temporal | Parahippocampal Gyrus | Impaired | Reduction | Cortical Thickness | Healthy Controls | - | - | Yes | (Guimond et al., 2016) |
| Temporal | Parahippocampal Gyrus | Impaired | Reduction | Cortical Thickness | Intact | - | - | Yes | (Guimond et al., 2016) |
| Temporal | Parahippocampal Gyrus | Intact | Reduction | Cortical Thickness | Healthy Controls | - | - | Yes | (Guimond et al., 2016) |
| Temporal | Parahippocampal Gyrus | Compromised | Reduction | Grey Matter Volume | Preserved | - | - | - | (Van Rheenen et al., 2018) |
| Temporal | Parahippocampal Gyrus | Compromised | Reduction | Grey Matter Volume | Deteriorated | - | - | - | (Van Rheenen et al., 2018) |
| Temporal | Right Banks of the Superior Temporal Sulcus | Preserved | No Difference | Cortical Thickness | Healthy Controls | - | - | - | (Ho et al., 2020) |
| Temporal | Right Banks of the Superior Temporal Sulcus | Deteriorated | Reduction | Cortical Thickness | Healthy Controls | - | - | - | (Ho et al., 2020) |
| Temporal | Right Banks of the Superior Temporal Sulcus | Compromised | No Difference | Cortical Thickness | Healthy Controls | - | - | - | (Ho et al., 2020) |
| Temporal | Right Banks of the Superior Temporal Sulcus | Deteriorated | No Difference | Cortical Thickness | Preserved | - | - | - | (Ho et al., 2020) |
| Temporal | Right Banks of the Superior Temporal Sulcus | Compromised | No Difference | Cortical Thickness | Preserved | - | - | - | (Ho et al., 2020) |
| Temporal | Right Banks of the Superior Temporal Sulcus | Compromised | No Difference | Cortical Thickness | Deteriorated | - | - | - | (Ho et al., 2020) |
| Temporal | Right Fusiform Gyrus | Deteriorated | Reduction | Grey Matter Volume | Healthy Controls | Yes | - | - | (Yasuda et al., 2020) |
| Temporal | Right Fusiform Gyrus | Preserved | No Difference | Grey Matter Volume | Healthy Controls | Yes | - | - | (Yasuda et al., 2020) |
| Temporal | Right Fusiform Gyrus | Deteriorated | Reduction | Grey Matter Volume | Preserved | Yes | - | - | (Yasuda et al., 2020) |
| Temporal | Right Hippocampus | Preserved | No Difference | Grey Matter Volume | Healthy Controls | Yes | - | - | (Ho et al., 2020) |
| Temporal | Right Hippocampus | Deteriorated | Reduction | Grey Matter Volume | Healthy Controls | Yes | - | - | (Ho et al., 2020) |
| Temporal | Right Hippocampus | Compromised | Reduction | Grey Matter Volume | Healthy Controls | Yes | - | - | (Ho et al., 2020) |
| Temporal | Right Hippocampus | Deteriorated | Reduction | Grey Matter Volume | Preserved | Yes | - | - | (Ho et al., 2020) |
| Temporal | Right Hippocampus | Compromised | No Difference | Grey Matter Volume | Preserved | Yes | - | - | (Ho et al., 2020) |
| Temporal | Right Hippocampus | Compromised | No Difference | Grey Matter Volume | Deteriorated | Yes | - | - | (Ho et al., 2020) |
| Temporal | Right Hippocampus | Deteriorated | Reduction | Grey Matter Volume | Healthy Controls | - | Yes | - | (Yasuda et al., 2020) |
| Temporal | Right Hippocampus | Preserved | Reduction | Grey Matter Volume | Healthy Controls | - | Yes | - | (Yasuda et al., 2020) |
| Temporal | Right Hippocampus | Deteriorated | No Difference | Grey Matter Volume | Preserved | - | Yes | - | (Yasuda et al., 2020) |
| Temporal | Right Hippocampus | Diminished Verbal/Motor Control | Reduction | Grey Matter Volume | Healthy Controls | - | - | - | (Geisler et al., 2015) |
| Temporal | Right Inferior Temporal Gyrus | Intact | Reduction | Grey Matter Volume | Healthy Controls | - | - | - | (Van Rheenen et al., 2018) |
| Temporal | Right Inferior Temporal Gyrus | Deteriorated | Reduction | Grey Matter Volume | Healthy Controls | - | - | - | (Yasuda et al., 2020) |
| Temporal | Right Inferior Temporal Gyrus | Preserved | No Difference | Grey Matter Volume | Healthy Controls | - | - | - | (Yasuda et al., 2020) |
| Temporal | Right Inferior Temporal Gyrus | Deteriorated | No Difference | Grey Matter Volume | Preserved | - | - | - | (Yasuda et al., 2020) |
| Temporal | Right Middle Temporal Gyrus | Preserved | Reduction | Cortical Thickness | Healthy Controls | - | Yes | - | (Ho et al., 2020) |
| Temporal | Right Middle Temporal Gyrus | Deteriorated | Reduction | Cortical Thickness | Healthy Controls | - | Yes | - | (Ho et al., 2020) |
| Temporal | Right Middle Temporal Gyrus | Compromised | No Difference | Cortical Thickness | Healthy Controls | - | Yes | - | (Ho et al., 2020) |
| Temporal | Right Middle Temporal Gyrus | Deteriorated | No Difference | Cortical Thickness | Preserved | - | Yes | - | (Ho et al., 2020) |
| Temporal | Right Middle Temporal Gyrus | Compromised | No Difference | Cortical Thickness | Preserved | - | Yes | - | (Ho et al., 2020) |
| Temporal | Right Middle Temporal Gyrus | Compromised | No Difference | Cortical Thickness | Deteriorated | - | Yes | - | (Ho et al., 2020) |
| Temporal | Right Middle Temporal Gyrus | Deteriorated | Reduction | Grey Matter Volume | Healthy Controls | - | - | - | (Yasuda et al., 2020) |
| Temporal | Right Middle Temporal Gyrus | Preserved | No Difference | Grey Matter Volume | Healthy Controls | - | - | - | (Yasuda et al., 2020) |
| Temporal | Right Middle Temporal Gyrus | Deteriorated | No Difference | Grey Matter Volume | Preserved | - | - | - | (Yasuda et al., 2020) |
| Temporal | Right Parahippocampal Gyrus | Deteriorated | Reduction | Grey Matter Volume | Healthy Controls | - | - | - | (Yasuda et al., 2020) |
| Temporal | Right Parahippocampal Gyrus | Preserved | No Difference | Grey Matter Volume | Healthy Controls | - | - | - | (Yasuda et al., 2020) |
| Temporal | Right Parahippocampal Gyrus | Deteriorated | No Difference | Grey Matter Volume | Preserved | - | - | - | (Yasuda et al., 2020) |
| Temporal | Right Superior Temporal Gyrus | Compromised | Reduction | Grey Matter Volume | Healthy Controls | - | - | - | (Van Rheenen et al., 2018) |
| Temporal | Right Superior Temporal Gyrus | Preserved | Reduction | Cortical Thickness | Healthy Controls | - | Yes | - | (Ho et al., 2020) |
| Temporal | Right Superior Temporal Gyrus | Deteriorated | Reduction | Cortical Thickness | Healthy Controls | - | Yes | - | (Ho et al., 2020) |
| Temporal | Right Superior Temporal Gyrus | Compromised | No Difference | Cortical Thickness | Healthy Controls | - | Yes | - | (Ho et al., 2020) |
| Temporal | Right Superior Temporal Gyrus | Deteriorated | No Difference | Cortical Thickness | Preserved | - | Yes | - | (Ho et al., 2020) |
| Temporal | Right Superior Temporal Gyrus | Compromised | No Difference | Cortical Thickness | Preserved | - | Yes | - | (Ho et al., 2020) |
| Temporal | Right Superior Temporal Gyrus | Compromised | No Difference | Cortical Thickness | Deteriorated | - | Yes | - | (Ho et al., 2020) |
| Temporal | Right Superior Temporal Gyrus | Deteriorated | Reduction | Grey Matter Volume | Healthy Controls | - | - | - | (Yasuda et al., 2020) |
| Temporal | Right Superior Temporal Gyrus | Preserved | No Difference | Grey Matter Volume | Healthy Controls | - | - | - | (Yasuda et al., 2020) |
| Temporal | Right Superior Temporal Gyrus | Deteriorated | No Difference | Grey Matter Volume | Preserved | - | - | - | (Yasuda et al., 2020) |
| Temporal | Right Temporal Lobe | Impaired | Reduction | Cortical Thickness | Healthy Controls | Yes | - | - | (Cobia et al., 2011) |
| Temporal | Right Temporal Lobe | Impaired | Reduction | Cortical Thickness | Intact | Yes | - | - | (Cobia et al., 2011) |
| Temporal | Superior Temporal | Severely Impaired | Reduction | Grey Matter Volume | Healthy Controls | - | - | - | (Weinberg et al., 2016) |
| Temporal | Superior Temporal | Severely Impaired | Reduction | Grey Matter Volume | Moderately Impaired | - | - | - | (Weinberg et al., 2016) |
| Temporal | Superior Temporal Gyrus | Impaired | Reduction | Cortical Thickness | Healthy Controls | - | - | - | (Cobia et al., 2011) |
| Temporal | Superior Temporal Gyrus | Impaired | Reduction | Grey Matter Volume | Healthy Controls | - | - | - | (Colibazzi et al., 2013) |
| Temporal | Temporal Pole | Compromised | Reduction | Grey Matter Volume | Preserved | - | - | - | (Van Rheenen et al., 2018) |
| Temporal | Temporal Pole | Compromised | Reduction | Grey Matter Volume | Deteriorated | - | - | - | (Van Rheenen et al., 2018) |
| Whole Brain | All Ventricles | Impaired | Increase | Ventricular Compartment Volume | Healthy Controls | - | - | - | (Wexler et al., 2009) |
| Whole Brain | Centrum Semiovale | Deteriorated | Reduction | White Matter Volume | Healthy Controls | - | - | - | (Woodward and Heckers, 2015) |
| Whole Brain | Cerebral Peduncles | Compromised | Reduction | White Matter Volume | Healthy Controls | - | - | - | (Woodward and Heckers, 2015) |
| Whole Brain | Corpus Callosum | Deteriorated | Reduction | White Matter Volume | Healthy Controls | - | - | - | (Woodward and Heckers, 2015) |
| Whole Brain | Inferior occipito-frontal and uncinate fasciculus to the genu of the corpus callosum | Intact | Reduction | White Matter Volume | Healthy Controls | - | - | - | (Alonso-Lana et al., 2016) |
| Whole Brain | Lateral Ventricles | Deteriorated | Reduction | White Matter Volume | Healthy Controls | - | - | - | (Woodward and Heckers, 2015) |
| Whole Brain | Left Cortex | Compromised | Reduction | Grey Matter Volume | Preserved | - | - | - | (Van Rheenen et al., 2018) |
| Whole Brain | Left Cortical Thickness | Deteriorated | Reduction | Total Cortical Thickness | Healthy Controls | Yes | - | - | (Yasuda et al., 2020) |
| Whole Brain | Left Cortical Thickness | Preserved | No Difference | Total Cortical Thickness | Healthy Controls | Yes | - | - | (Yasuda et al., 2020) |
| Whole Brain | Left Cortical Thickness | Deteriorated | Reduction | Total Cortical Thickness | Preserved | Yes | - | - | (Yasuda et al., 2020) |
| Whole Brain | Left Lateral Ventricle | Deteriorated | Increase | Ventricle Volume | Healthy Controls | - | Yes | - | (Yasuda et al., 2020) |
| Whole Brain | Left Lateral Ventricle | Preserved | Increase | Ventricle Volume | Healthy Controls | - | Yes | - | (Yasuda et al., 2020) |
| Whole Brain | Left Lateral Ventricle | Deteriorated | No Difference | Ventricle Volume | Preserved | - | Yes | - | (Yasuda et al., 2020) |
| Whole Brain | Periventricular | Deteriorated | Reduction | White Matter Volume | Healthy Controls | - | - | - | (Woodward and Heckers, 2015) |
| Whole Brain | Pons | Compromised | Reduction | White Matter Volume | Healthy Controls | - | - | - | (Woodward and Heckers, 2015) |
| Whole Brain | Posterior Periventricular | Compromised | Reduction | White Matter Volume | Healthy Controls | - | - | - | (Woodward and Heckers, 2015) |
| Whole Brain | Right Cortex | Compromised | Reduction | Grey Matter Volume | Deteriorated | - | - | - | (Van Rheenen et al., 2018) |
| Whole Brain | Right Cortical Thickness | Deteriorated | Reduction | Total Cortical Thickness | Healthy Controls | Yes | - | - | (Yasuda et al., 2020) |
| Whole Brain | Right Cortical Thickness | Preserved | No Difference | Total Cortical Thickness | Healthy Controls | Yes | - | - | (Yasuda et al., 2020) |
| Whole Brain | Right Cortical Thickness | Deteriorated | Reduction | Total Cortical Thickness | Preserved | Yes | - | - | (Yasuda et al., 2020) |
| Whole Brain | Right Hemisphere | Impaired | Reduction | White Matter Volume | Healthy Controls | - | - | - | (Colibazzi et al., 2013) |
| Whole Brain | Right Lateral Ventricle | Deteriorated | Increase | Ventricle Volume | Healthy Controls | Yes | - | - | (Yasuda et al., 2020) |
| Whole Brain | Right Lateral Ventricle | Preserved | No Difference | Ventricle Volume | Healthy Controls | Yes | - | - | (Yasuda et al., 2020) |
| Whole Brain | Right Lateral Ventricle | Deteriorated | Increase | Ventricle Volume | Preserved | Yes | - | - | (Yasuda et al., 2020) |
| Whole Brain | Splenium of the Corpus Callosum | Compromised | Reduction | White Matter Volume | Healthy Controls | - | - | - | (Woodward and Heckers, 2015) |
| Whole Brain | Third Ventricles | Intact | Increase | Ventricular Compartment Volume | Healthy Controls | - | Yes | - | (Wexler et al., 2009) |
| Whole Brain |  | Intact | No Difference | Total Grey Matter Volume | Impaired | - | - | - | (Alonso-Lana et al., 2016) |
| Whole Brain |  | Impaired | Reduction | Total Grey Matter Volume | Intact | - | - | - | (Ayesa-Arriola et al., 2013) |
| Whole Brain |  | Impaired | No Difference | Total Cortical Thickness | Intact | - | - | - | (Colibazzi et al., 2013) |
| Whole Brain |  | Preserved | No Difference | Cortical White Matter Volume | Healthy Controls | Yes | - | - | (Czepielewski et al., 2017) |
| Whole Brain |  | Deteriorated | No Difference | Cortical White Matter Volume | Healthy Controls | Yes | - | - | (Czepielewski et al., 2017) |
| Whole Brain |  | Compromised | Increase | Cortical White Matter Volume | Healthy Controls | Yes | - | - | (Czepielewski et al., 2017) |
| Whole Brain |  | Deteriorated | Increase | Cortical White Matter Volume | Preserved | Yes | - | - | (Czepielewski et al., 2017) |
| Whole Brain |  | Compromised | Increase | Cortical White Matter Volume | Preserved | Yes | - | - | (Czepielewski et al., 2017) |
| Whole Brain |  | Compromised | Increase | Cortical White Matter Volume | Deteriorated | Yes | - | - | (Czepielewski et al., 2017) |
| Whole Brain |  | Preserved | No Difference | Intracranial Brain Volume | Healthy Controls | - | - | - | (Czepielewski et al., 2017) |
| Whole Brain |  | Deteriorated | No Difference | Intracranial Brain Volume | Healthy Controls | - | - | - | (Czepielewski et al., 2017) |
| Whole Brain |  | Compromised | Reduction | Intracranial Brain Volume | Healthy Controls | - | - | - | (Czepielewski et al., 2017) |
| Whole Brain |  | Deteriorated | No Difference | Intracranial Brain Volume | Preserved | - | - | - | (Czepielewski et al., 2017) |
| Whole Brain |  | Compromised | No Difference | Intracranial Brain Volume | Preserved | - | - | - | (Czepielewski et al., 2017) |
| Whole Brain |  | Compromised | No Difference | Intracranial Brain Volume | Deteriorated | - | - | - | (Czepielewski et al., 2017) |
| Whole Brain |  | Preserved | Reduction | Total Brain Volume | Healthy Controls | - | Yes | - | (Czepielewski et al., 2017) |
| Whole Brain |  | Deteriorated | No Difference | Total Brain Volume | Healthy Controls | - | Yes | - | (Czepielewski et al., 2017) |
| Whole Brain |  | Compromised | Reduction | Total Brain Volume | Healthy Controls | - | Yes | - | (Czepielewski et al., 2017) |
| Whole Brain |  | Deteriorated | No Difference | Total Brain Volume | Preserved | - | Yes | - | (Czepielewski et al., 2017) |
| Whole Brain |  | Compromised | No Difference | Total Brain Volume | Preserved | - | Yes | - | (Czepielewski et al., 2017) |
| Whole Brain |  | Compromised | Reduction | Total Brain Volume | Deteriorated | - | Yes | - | (Czepielewski et al., 2017) |
| Whole Brain |  | Preserved | Reduction | Total Brain Volume (corrected) | Healthy Controls | - | Yes | - | (Czepielewski et al., 2017) |
| Whole Brain |  | Deteriorated | No Difference | Total Brain Volume (corrected) | Healthy Controls | - | Yes | - | (Czepielewski et al., 2017) |
| Whole Brain |  | Compromised | Reduction | Total Brain Volume (corrected) | Healthy Controls | - | Yes | - | (Czepielewski et al., 2017) |
| Whole Brain |  | Deteriorated | No Difference | Total Brain Volume (corrected) | Preserved | - | Yes | - | (Czepielewski et al., 2017) |
| Whole Brain |  | Compromised | No Difference | Total Brain Volume (corrected) | Preserved | - | Yes | - | (Czepielewski et al., 2017) |
| Whole Brain |  | Compromised | Reduction | Total Brain Volume (corrected) | Deteriorated | - | Yes | - | (Czepielewski et al., 2017) |
| Whole Brain |  | Preserved | No Difference | Total Cortical Grey Matter | Healthy Controls | Yes | - | - | (Czepielewski et al., 2017) |
| Whole Brain |  | Deteriorated | Reduction | Total Cortical Grey Matter | Healthy Controls | Yes | - | - | (Czepielewski et al., 2017) |
| Whole Brain |  | Compromised | Reduction | Total Cortical Grey Matter | Healthy Controls | Yes | - | - | (Czepielewski et al., 2017) |
| Whole Brain |  | Deteriorated | No Difference | Total Cortical Grey Matter | Preserved | Yes | - | - | (Czepielewski et al., 2017) |
| Whole Brain |  | Compromised | Reduction | Total Cortical Grey Matter | Preserved | Yes | - | - | (Czepielewski et al., 2017) |
| Whole Brain |  | Compromised | Reduction | Total Cortical Grey Matter | Deteriorated | Yes | - | - | (Czepielewski et al., 2017) |
| Whole Brain |  | Preserved | No Difference | Total Cortical Thickness | Healthy Controls | Yes | - | - | (Czepielewski et al., 2017) |
| Whole Brain |  | Deteriorated | Reduction | Total Cortical Thickness | Healthy Controls | Yes | - | - | (Czepielewski et al., 2017) |
| Whole Brain |  | Compromised | Reduction | Total Cortical Thickness | Healthy Controls | Yes | - | - | (Czepielewski et al., 2017) |
| Whole Brain |  | Deteriorated | No Difference | Total Cortical Thickness | Preserved | Yes | - | - | (Czepielewski et al., 2017) |
| Whole Brain |  | Compromised | Reduction | Total Cortical Thickness | Preserved | Yes | - | - | (Czepielewski et al., 2017) |
| Whole Brain |  | Compromised | No Difference | Total Cortical Thickness | Deteriorated | Yes | - | - | (Czepielewski et al., 2017) |
| Whole Brain |  | Preserved | No Difference | Total Grey Matter Volume | Healthy Controls | Yes | - | - | (Czepielewski et al., 2017) |
| Whole Brain |  | Deteriorated | Reduction | Total Grey Matter Volume | Healthy Controls | Yes | - | - | (Czepielewski et al., 2017) |
| Whole Brain |  | Compromised | Reduction | Total Grey Matter Volume | Healthy Controls | Yes | - | - | (Czepielewski et al., 2017) |
| Whole Brain |  | Deteriorated | Reduction | Total Grey Matter Volume | Preserved | Yes | - | - | (Czepielewski et al., 2017) |
| Whole Brain |  | Compromised | Reduction | Total Grey Matter Volume | Preserved | Yes | - | - | (Czepielewski et al., 2017) |
| Whole Brain |  | Compromised | Reduction | Total Grey Matter Volume | Deteriorated | Yes | - | - | (Czepielewski et al., 2017) |
| Whole Brain |  | Preserved | No Difference | Total Subcortical Grey Matter Volume | Healthy Controls | - | - | - | (Czepielewski et al., 2017) |
| Whole Brain |  | Deteriorated | No Difference | Total Subcortical Grey Matter Volume | Healthy Controls | - | - | - | (Czepielewski et al., 2017) |
| Whole Brain |  | Compromised | No Difference | Total Subcortical Grey Matter Volume | Healthy Controls | - | - | - | (Czepielewski et al., 2017) |
| Whole Brain |  | Deteriorated | No Difference | Total Subcortical Grey Matter Volume | Preserved | - | - | - | (Czepielewski et al., 2017) |
| Whole Brain |  | Compromised | No Difference | Total Subcortical Grey Matter Volume | Preserved | - | - | - | (Czepielewski et al., 2017) |
| Whole Brain |  | Compromised | No Difference | Total Subcortical Grey Matter Volume | Deteriorated | - | - | - | (Czepielewski et al., 2017) |
| Whole Brain |  | Preserved | No Difference | Total White Matter Volume | Healthy Controls | Yes | - | - | (Czepielewski et al., 2017) |
| Whole Brain |  | Deteriorated | Increase | Total White Matter Volume | Healthy Controls | Yes | - | - | (Czepielewski et al., 2017) |
| Whole Brain |  | Compromised | Increase | Total White Matter Volume | Healthy Controls | Yes | - | - | (Czepielewski et al., 2017) |
| Whole Brain |  | Deteriorated | Increase | Total White Matter Volume | Preserved | Yes | - | - | (Czepielewski et al., 2017) |
| Whole Brain |  | Compromised | Increase | Total White Matter Volume | Preserved | Yes | - | - | (Czepielewski et al., 2017) |
| Whole Brain |  | Compromised | Increase | Total White Matter Volume | Deteriorated | Yes | - | - | (Czepielewski et al., 2017) |
| Whole Brain |  | Diminished Verbal/Motor Control | No Difference | Cortical Thickness | Healthy Controls | - | - | - | (Geisler et al., 2015) |
| Whole Brain |  | Diminished Intellectual Functioning | Reduction | Cortical Thickness | Healthy Controls | - | - | - | (Geisler et al., 2015) |
| Whole Brain |  | Intact | Reduction | Grey Matter Volume | Healthy Controls | - | - | Yes | (Gould et al., 2014) |
| Whole Brain |  | Impaired | Reduction | Grey Matter Volume | Healthy Controls | - | - | Yes | (Gould et al., 2014) |
| Whole Brain |  | Impaired | Reduction | Grey Matter Volume | Intact | - | - | Yes | (Gould et al., 2014) |
| Whole Brain |  | Intact | Reduction | Total Brain Volume | Healthy Controls | - | - | Yes | (Gould et al., 2014) |
| Whole Brain |  | Impaired | Reduction | Total Brain Volume | Healthy Controls | - | - | Yes | (Gould et al., 2014) |
| Whole Brain |  | Impaired | Reduction | Total Brain Volume | Intact | - | - | Yes | (Gould et al., 2014) |
| Whole Brain |  | Intact | Reduction | White Matter Volume | Healthy Controls | - | - | Yes | (Gould et al., 2014) |
| Whole Brain |  | Impaired | Reduction | White Matter Volume | Healthy Controls | - | - | Yes | (Gould et al., 2014) |
| Whole Brain |  | Impaired | Reduction | White Matter Volume | Intact | - | - | Yes | (Gould et al., 2014) |
| Whole Brain |  | Intact | No Difference | Grey Matter Volume | Impaired | - | - | - | (Ortiz-Gil et al., 2011) |
| Whole Brain |  | Intact | No Difference | White Matter Volume | Healthy Controls | - | - | - | (Ortiz-Gil et al., 2011) |
| Whole Brain |  | Intact | No Difference | White Matter Volume | Impaired | - | - | - | (Ortiz-Gil et al., 2011) |
| Whole Brain |  | Impaired | No Difference | White Matter Volume | Intact | - | - | - | (Poletti et al., 2014) |
| Whole Brain |  | Intact | Reduction | Total Grey Matter Volume | Healthy Controls | - | Yes | - | (Rusch et al., 2007) |
| Whole Brain |  | Impaired | Reduction | Total Grey Matter Volume | Healthy Controls | - | Yes | - | (Rusch et al., 2007) |
| Whole Brain |  | Impaired | No Difference | Total Grey Matter Volume | Intact | - | Yes | - | (Rusch et al., 2007) |
| Whole Brain |  | Impaired | No Difference | White Matter Volume | Intact | - | - | - | (Shepherd et al., 2015) |
| Whole Brain |  | Intact | No Difference | White Matter Volume | Healthy Controls | - | - | - | (Shepherd et al., 2015) |
| Whole Brain |  | Impaired | No Difference | White Matter Volume | Healthy Controls | - | - | - | (Shepherd et al., 2015) |
| Whole Brain |  | Intact | No Difference | Grey Matter Volume | Impaired | - | - | - | (Torres et al., 1997) |
| Whole Brain |  | Preserved | No Difference | Intracranial Brain Volume | Healthy Controls | - | - | - | (Van Rheenen et al., 2018) |
| Whole Brain |  | Compromised | No Difference | Intracranial Brain Volume | Healthy Controls | - | - | - | (Van Rheenen et al., 2018) |
| Whole Brain |  | Deteriorated | No Difference | Intracranial Brain Volume | Healthy Controls | - | - | - | (Van Rheenen et al., 2018) |
| Whole Brain |  | Preserved | Reduction | Intracranial Brain Volume (corrected) | Healthy Controls | - | Yes | - | (Van Rheenen et al., 2018) |
| Whole Brain |  | Compromised | Reduction | Intracranial Brain Volume (corrected) | Healthy Controls | - | Yes | - | (Van Rheenen et al., 2018) |
| Whole Brain |  | Deteriorated | Reduction | Intracranial Brain Volume (corrected) | Healthy Controls | - | Yes | - | (Van Rheenen et al., 2018) |
| Whole Brain |  | Preserved | No Difference | Total Brain Volume (corrected) | Healthy Controls | - | - | - | (Van Rheenen et al., 2018) |
| Whole Brain |  | Compromised | No Difference | Total Brain Volume (corrected) | Healthy Controls | - | - | - | (Van Rheenen et al., 2018) |
| Whole Brain |  | Deteriorated | No Difference | Total Brain Volume (corrected) | Healthy Controls | - | - | - | (Van Rheenen et al., 2018) |
| Whole Brain |  | Preserved | No Difference | Total Grey Matter Volume | Healthy Controls | - | - | - | (Van Rheenen et al., 2018) |
| Whole Brain |  | Compromised | No Difference | Total Grey Matter Volume | Healthy Controls | - | - | - | (Van Rheenen et al., 2018) |
| Whole Brain |  | Deteriorated | No Difference | Total Grey Matter Volume | Healthy Controls | - | - | - | (Van Rheenen et al., 2018) |
| Whole Brain |  | Preserved | No Difference | White Matter Volume | Healthy Controls | - | - | - | (Van Rheenen et al., 2018) |
| Whole Brain |  | Compromised | No Difference | White Matter Volume | Healthy Controls | - | - | - | (Van Rheenen et al., 2018) |
| Whole Brain |  | Deteriorated | No Difference | White Matter Volume | Healthy Controls | - | - | - | (Van Rheenen et al., 2018) |
| Whole Brain |  | Compromised | Reduction | White Matter Volume | Preserved | - | - | - | (Van Rheenen et al., 2018) |
| Whole Brain |  | Compromised | Reduction | White Matter Volume | Deteriorated | - | - | - | (Van Rheenen et al., 2018) |
| Whole Brain |  | Deteriorated | Reduction | White Matter Volume | Preserved | - | - | - | (Van Rheenen et al., 2018) |
| Whole Brain |  | Preserved | No Difference | Cortical Grey Matter Volume | Healthy Controls | - | - | - | (Weinberg et al., 2016) |
| Whole Brain |  | Moderately Impaired | No Difference | Cortical Grey Matter Volume | Healthy Controls | - | - | - | (Weinberg et al., 2016) |
| Whole Brain |  | Severely Impaired | Reduction | Cortical Grey Matter Volume | Healthy Controls | - | - | - | (Weinberg et al., 2016) |
| Whole Brain |  | Moderately Impaired | No Difference | Cortical Grey Matter Volume | Preserved | - | - | - | (Weinberg et al., 2016) |
| Whole Brain |  | Severely Impaired | No Difference | Cortical Grey Matter Volume | Preserved | - | - | - | (Weinberg et al., 2016) |
| Whole Brain |  | Severely Impaired | No Difference | Cortical Grey Matter Volume | Moderately Impaired | - | - | - | (Weinberg et al., 2016) |
| Whole Brain |  | Preserved | No Difference | Cortical White Matter Volume | Healthy Controls | - | - | - | (Weinberg et al., 2016) |
| Whole Brain |  | Moderately Impaired | No Difference | Cortical White Matter Volume | Healthy Controls | - | - | - | (Weinberg et al., 2016) |
| Whole Brain |  | Severely Impaired | Reduction | Cortical White Matter Volume | Healthy Controls | - | - | - | (Weinberg et al., 2016) |
| Whole Brain |  | Moderately Impaired | No Difference | Cortical White Matter Volume | Preserved | - | - | - | (Weinberg et al., 2016) |
| Whole Brain |  | Severely Impaired | No Difference | Cortical White Matter Volume | Preserved | - | - | - | (Weinberg et al., 2016) |
| Whole Brain |  | Severely Impaired | No Difference | Cortical White Matter Volume | Moderately Impaired | - | - | - | (Weinberg et al., 2016) |
| Whole Brain |  | Preserved | No Difference | Intracranial Brain Volume | Healthy Controls | - | - | - | (Weinberg et al., 2016) |
| Whole Brain |  | Moderately Impaired | No Difference | Intracranial Brain Volume | Healthy Controls | - | - | - | (Weinberg et al., 2016) |
| Whole Brain |  | Severely Impaired | No Difference | Intracranial Brain Volume | Healthy Controls | - | - | - | (Weinberg et al., 2016) |
| Whole Brain |  | Moderately Impaired | No Difference | Intracranial Brain Volume | Preserved | - | - | - | (Weinberg et al., 2016) |
| Whole Brain |  | Severely Impaired | No Difference | Intracranial Brain Volume | Preserved | - | - | - | (Weinberg et al., 2016) |
| Whole Brain |  | Severely Impaired | No Difference | Intracranial Brain Volume | Moderately Impaired | - | - | - | (Weinberg et al., 2016) |
| Whole Brain |  | Preserved | No Difference | Total Grey Matter Volume | Healthy Controls | - | - | - | (Weinberg et al., 2016) |
| Whole Brain |  | Moderately Impaired | No Difference | Total Grey Matter Volume | Healthy Controls | - | - | - | (Weinberg et al., 2016) |
| Whole Brain |  | Severely Impaired | Reduction | Total Grey Matter Volume | Healthy Controls | - | - | - | (Weinberg et al., 2016) |
| Whole Brain |  | Moderately Impaired | No Difference | Total Grey Matter Volume | Preserved | - | - | - | (Weinberg et al., 2016) |
| Whole Brain |  | Severely Impaired | No Difference | Total Grey Matter Volume | Preserved | - | - | - | (Weinberg et al., 2016) |
| Whole Brain |  | Severely Impaired | No Difference | Total Grey Matter Volume | Moderately Impaired | - | - | - | (Weinberg et al., 2016) |
| Whole Brain |  | Intact | Reduction | Grey Matter Volume | Healthy Controls | - | Yes | - | (Wexler et al., 2009) |
| Whole Brain |  | Impaired | Reduction | Grey Matter Volume | Healthy Controls | - | Yes | - | (Wexler et al., 2009) |
| Whole Brain |  | Preserved | No Difference | Total Brain Volume | Healthy Controls | - | - | - | (Ho et al., 2020) |
| Whole Brain |  | Deteriorated | Reduction | Total Brain Volume | Healthy Controls | - | - | - | (Ho et al., 2020) |
| Whole Brain |  | Compromised | No Difference | Total Brain Volume | Healthy Controls | - | - | - | (Ho et al., 2020) |
| Whole Brain |  | Deteriorated | No Difference | Total Brain Volume | Preserved | - | - | - | (Ho et al., 2020) |
| Whole Brain |  | Compromised | No Difference | Total Brain Volume | Preserved | - | - | - | (Ho et al., 2020) |
| Whole Brain |  | Compromised | No Difference | Total Brain Volume | Deteriorated | - | - | - | (Ho et al., 2020) |
| Whole Brain |  | Preserved | Reduction | Total Brain Volume (corrected) | Healthy Controls | - | Yes | - | (Ho et al., 2020) |
| Whole Brain |  | Deteriorated | Reduction | Total Brain Volume (corrected) | Healthy Controls | - | Yes | - | (Ho et al., 2020) |
| Whole Brain |  | Compromised | No Difference | Total Brain Volume (corrected) | Healthy Controls | - | Yes | - | (Ho et al., 2020) |
| Whole Brain |  | Deteriorated | No Difference | Total Brain Volume (corrected) | Preserved | - | Yes | - | (Ho et al., 2020) |
| Whole Brain |  | Compromised | No Difference | Total Brain Volume (corrected) | Preserved | - | Yes | - | (Ho et al., 2020) |
| Whole Brain |  | Compromised | No Difference | Total Brain Volume (corrected) | Deteriorated | - | Yes | - | (Ho et al., 2020) |
| Whole Brain |  | Preserved | Reduction | Total Cortical Grey Matter Volume (corrected) | Healthy Controls | - | Yes | - | (Ho et al., 2020) |
| Whole Brain |  | Deteriorated | Reduction | Total Cortical Grey Matter Volume (corrected) | Healthy Controls | - | Yes | - | (Ho et al., 2020) |
| Whole Brain |  | Compromised | No Difference | Total Cortical Grey Matter Volume (corrected) | Healthy Controls | - | Yes | - | (Ho et al., 2020) |
| Whole Brain |  | Deteriorated | No Difference | Total Cortical Grey Matter Volume (corrected) | Preserved | - | Yes | - | (Ho et al., 2020) |
| Whole Brain |  | Compromised | No Difference | Total Cortical Grey Matter Volume (corrected) | Preserved | - | Yes | - | (Ho et al., 2020) |
| Whole Brain |  | Compromised | No Difference | Total Cortical Grey Matter Volume (corrected) | Deteriorated | - | Yes | - | (Ho et al., 2020) |
| Whole Brain |  | Preserved | No Difference | Total Subcortical Volume | Healthy Controls | - | - | - | (Ho et al., 2020) |
| Whole Brain |  | Deteriorated | Reduction | Total Subcortical Volume | Healthy Controls | - | - | - | (Ho et al., 2020) |
| Whole Brain |  | Compromised | No Difference | Total Subcortical Volume | Healthy Controls | - | - | - | (Ho et al., 2020) |
| Whole Brain |  | Deteriorated | No Difference | Total Subcortical Volume | Preserved | - | - | - | (Ho et al., 2020) |
| Whole Brain |  | Compromised | No Difference | Total Subcortical Volume | Preserved | - | - | - | (Ho et al., 2020) |
| Whole Brain |  | Compromised | No Difference | Total Subcortical Volume | Deteriorated | - | - | - | (Ho et al., 2020) |
| Whole Brain |  | Preserved | No Difference | Total Cortical White Matter Volume | Healthy Controls | - | - | - | (Ho et al., 2020) |
| Whole Brain |  | Deteriorated | Reduction | Total Cortical White Matter Volume | Healthy Controls | - | - | - | (Ho et al., 2020) |
| Whole Brain |  | Compromised | No Difference | Total Cortical White Matter Volume | Healthy Controls | - | - | - | (Ho et al., 2020) |
| Whole Brain |  | Deteriorated | No Difference | Total Cortical White Matter Volume | Preserved | - | - | - | (Ho et al., 2020) |
| Whole Brain |  | Compromised | No Difference | Total Cortical White Matter Volume | Preserved | - | - | - | (Ho et al., 2020) |
| Whole Brain |  | Compromised | No Difference | Total Cortical White Matter Volume | Deteriorated | - | - | - | (Ho et al., 2020) |
| Whole Brain |  | Deteriorated | Reduction | Total Brain Volume | Healthy Controls | - | - | - | (Yasuda et al., 2020) |
| Whole Brain |  | Preserved | No Difference | Total Brain Volume | Healthy Controls | - | - | - | (Yasuda et al., 2020) |
| Whole Brain |  | Deteriorated | No Difference | Total Brain Volume | Preserved | - | - | - | (Yasuda et al., 2020) |
| Whole Brain |  | Deteriorated | Reduction | Total Grey Matter Volume | Healthy Controls | Yes | - | - | (Yasuda et al., 2020) |
| Whole Brain |  | Preserved | No Difference | Total Grey Matter Volume | Healthy Controls | Yes | - | - | (Yasuda et al., 2020) |
| Whole Brain |  | Deteriorated | Reduction | Total Grey Matter Volume | Preserved | Yes | - | - | (Yasuda et al., 2020) |
| Whole Brain |  | Deteriorated | Reduction | Total Cortical Grey Matter Volume | Healthy Controls | Yes | - | - | (Yasuda et al., 2020) |
| Whole Brain |  | Preserved | No Difference | Total Cortical Grey Matter Volume | Healthy Controls | Yes | - | - | (Yasuda et al., 2020) |
| Whole Brain |  | Deteriorated | Reduction | Total Cortical Grey Matter Volume | Preserved | Yes | - | - | (Yasuda et al., 2020) |
| Whole Brain |  | Compromised | Reduction | Intracranial Brain Volume | Healthy Controls | Yes | - | - | (Woodward and Heckers, 2015) |
| Whole Brain |  | Deteriorated | No Difference | Intracranial Brain Volume | Healthy Controls | Yes | - | - | (Woodward and Heckers, 2015) |
| Whole Brain |  | Preserved | No Difference | Intracranial Brain Volume | Healthy Controls | Yes | - | - | (Woodward and Heckers, 2015) |
| Whole Brain |  | Compromised | Reduction | Intracranial Brain Volume | Deteriorated | Yes | - | - | (Woodward and Heckers, 2015) |
| Whole Brain |  | Compromised | Reduction | Intracranial Brain Volume | Preserved | Yes | - | - | (Woodward and Heckers, 2015) |
| Whole Brain |  | Compromised | Reduction | Total Brain Volume | Healthy Controls | Yes | - | - | (Woodward and Heckers, 2015) |
| Whole Brain |  | Deteriorated | No Difference | Total Brain Volume | Healthy Controls | Yes | - | - | (Woodward and Heckers, 2015) |
| Whole Brain |  | Preserved | No Difference | Total Brain Volume | Healthy Controls | Yes | - | - | (Woodward and Heckers, 2015) |
| Whole Brain |  | Compromised | Reduction | Total Brain Volume | Preserved | Yes | - | - | (Woodward and Heckers, 2015) |
| Whole Brain |  | Compromised | No Difference | Total Brain Volume (corrected) | Healthy Controls | - | Yes | - | (Woodward and Heckers, 2015) |
| Whole Brain |  | Deteriorated | Reduction | Total Brain Volume (corrected) | Healthy Controls | - | Yes | - | (Woodward and Heckers, 2015) |
| Whole Brain |  | Preserved | Reduction | Total Brain Volume (corrected) | Healthy Controls | - | Yes | - | (Woodward and Heckers, 2015) |
| Whole Brain |  | Compromised | No Difference | Total Grey Matter Volume | Healthy Controls | - | Yes | - | (Woodward and Heckers, 2015) |
| Whole Brain |  | Deteriorated | Reduction | Total Grey Matter Volume | Healthy Controls | - | Yes | - | (Woodward and Heckers, 2015) |
| Whole Brain |  | Preserved | Reduction | Total Grey Matter Volume | Healthy Controls | - | Yes | - | (Woodward and Heckers, 2015) |
| Whole Brain |  | Compromised | No Difference | Total White Matter Volume | Healthy Controls | - | Yes | - | (Woodward and Heckers, 2015) |
| Whole Brain |  | Deteriorated | Reduction | Total White Matter Volume | Healthy Controls | - | Yes | - | (Woodward and Heckers, 2015) |
| Whole Brain |  | Preserved | Reduction | Total White Matter Volume | Healthy Controls | - | Yes | - | (Woodward and Heckers, 2015) |

*Note.* This table of findings works in conjunction with Figure 1, and infers if regions are associated with cognitive impairment (Figure 1a), disease presence (Figure 1b), or the interaction between these two (Figure 1c). Findings are organized by ‘lobe’ and then ‘region in alphabetical order, and are only reflect what is reported in each given paper. Banding of rows is to increase readability. When ‘Whole Brain’ is listed for lobe, but region is blank it is indicative that no significant association was identified in any region/cluster across the entire brain; HC = healthy controls; ‘≠’ is indicative that there is a significant difference in morphology between (sub)groups; ‘=’ is indicative that there is no significant difference in morphology between (sub)groups. The dash ‘ – ’ indicates that with the available evidence, the relevant findings do not support our models presented in Figure 1. For brevity, the term ‘intact’ is used synonymously with ‘relatively intact’.

Alonso-Lana, Goikolea, Bonnin, Sarro, Segura, Amann, Monte, Moro, Fernandez-Corcuera, Maristany, Salvador, Vieta, Pomarol-Clotet, & McKenna. (2016). Structural and Functional Brain Correlates of Cognitive Impairment in Euthymic Patients with Bipolar Disorder. *PLoS One, 11*(7), e0158867. doi: 10.1371/journal.pone.0158867

Ayesa-Arriola, Roiz-Santianez, Perez-Iglesias, Ferro, Sainz, & Crespo-Facorro. (2013). Neuroanatomical Differences between First-Episode Psychosis Patients with and without Neurocognitive Deficit: A 3-Year Longitudinal Study. *Front Psychiatry, 4*, 134. doi: 10.3389/fpsyt.2013.00134

Cobia, Csernansky, & Wang. (2011). Cortical thickness in neuropsychologically near-normal schizophrenia. *Schizophrenia Research, 133*(1), 68-76. doi: 10.1016/j.schres.2011.08.017

Colibazzi, Wexler, Bansal, Hao, Liu, Sanchez-Peña, Corcoran, Lieberman, & Peterson. (2013). Anatomical Abnormalities in Gray and White Matter of the Cortical Surface in Persons with Schizophrenia. *PLoS One, 8*(2). doi: 10.1371/journal.pone.0055783

Czepielewski, Wang, Gama, & Barch. (2017). The Relationship of Intellectual Functioning and Cognitive Performance to Brain Structure in Schizophrenia. *Schizophr Bull, 43*(2), 355-364. doi: 10.1093/schbul/sbw090

Geisler, Walton, Naylor, Roessner, Lim, Charles Schulz, Gollub, Calhoun, Sponheim, & Ehrlich. (2015). Brain structure and function correlates of cognitive subtypes in schizophrenia. *Psychiatry Res, 234*(1), 74-83. doi: 10.1016/j.pscychresns.2015.08.008

Gould, Shepherd, Laurens, Cairns, Carr, & Green. (2014). Multivariate neuroanatomical classification of cognitive subtypes in schizophrenia: a support vector machine learning approach. *Neuroimage: Clinical, 6*, 229-236. doi: 10.1016/j.nicl.2014.09.009

Guimond, Chakravarty, Bergeron-Gagnon, Patel, & Lepage. (2016). Verbal memory impairments in schizophrenia associated with cortical thinning. *Neuroimage: Clinical, 11*, 20-29. doi: 10.1016/j.nicl.2015.12.010

Ho, Lee, Tng, Lam, Chen, Wang, Zhou, Keefe, & Sim. (2020). Corticolimbic brain anomalies are associated with cognitive subtypes in psychosis: A longitudinal study. *European Psychiatry, 63*(1), e40. doi: 10.1192/j.eurpsy.2020.36

Ortiz-Gil, Pomarol-Clotet, Salvador, Canales-Rodriguez, Sarro, Gomar, Guerrero, Sans-Sansa, Capdevila, Junque, & McKenna. (2011). Neural correlates of cognitive impairment in schizophrenia. *British Joural of Psychiatry, 199*(3), 202-210. doi: 10.1192/bjp.bp.110.083600

Poletti, Radaelli, Bosia, Buonocore, Pirovano, Lorenzi, Cavallaro, Smeraldi, & Benedetti. (2014). Effect of glutamate transporter EAAT2 gene variants and gray matter deficits on working memory in schizophrenia. *European Psychiatry, 29*(4), 219-225. doi: 10.1016/j.eurpsy.2013.07.003

Rusch, Spoletini, Wilke, Bria, Di Paola, Di Iulio, Martinotti, Caltagirone, & Spalletta. (2007). Prefrontal-thalamic-cerebellar gray matter networks and executive functioning in schizophrenia. *Schizophr Res, 93*(1-3), 79-89. doi: 10.1016/j.schres.2007.01.029

Shepherd, Quide, Laurens, O'Reilly, Rowland, Mitchell, Carr, & Green. (2015). Shared intermediate phenotypes for schizophrenia and bipolar disorder: neuroanatomical features of subtypes distinguished by executive dysfunction. *J Psychiatry Neurosci, 40*(1), 58-68.

Torres, Flashman, O’Leary, Swayze, & Andreasen. (1997). Lack of an association between delayed memory and hippocampal and temporal lobe size in patients with schizophrenia and healthy controls. *Society of Biological Psychiatry, 42*, 1087-1096.

Van Rheenen, Cropley, Zalesky, Bousman, Wells, Bruggemann, Sundram, Weinberg, Lenroot, Pereira, Shannon Weickert, Weickert, & Pantelis. (2018). Widespread Volumetric Reductions in Schizophrenia and Schizoaffective Patients Displaying Compromised Cognitive Abilities. *Schizophrenia Bulletin, 44*(3), 560-574. doi: 10.1093/schbul/sbx109

Vaskinn, Hartberg, Sundet, Westlye, Andreassen, Melle, & Agartz. (2015). Brain structure characteristics in intellectually superior schizophrenia. *Psychiatry Res, 232*(1), 123-129. doi: 10.1016/j.pscychresns.2015.02.005

Weinberg, Lenroot, Jacomb, Allen, Bruggemann, Wells, Balzan, Liu, Galletly, Catts, Weickert, & Weickert. (2016). Cognitive Subtypes of Schizophrenia Characterized by Differential Brain Volumetric Reductions and Cognitive Decline. *JAMA Psychiatry, 73*(12), 1251-1259. doi: 10.1001/jamapsychiatry.2016.2925

Wexler, Zhu, Bell, Nicholls, Fulbright, Gore, Colibazzi, Amat, Bansal, & Peterson. (2009). Neuropsychological near normality and brain structure abnormality in schizophrenia. *Am J Psychiatry, 166*(2), 189-195. doi: 10.1176/appi.ajp.2008.08020258

Woodward, & Heckers. (2015). Brain Structure in Neuropsychologically Defined Subgroups of Schizophrenia and Psychotic Bipolar Disorder. *Schizophrenia Bulletin, 41*(6), 1349-1359. doi: 10.1093/schbul/sbv048

Yasuda, Okada, Nemoto, Fukunaga, Yamamori, Ohi, Koshiyama, Kudo, Shiino, Morita, Morita, Azechi, Fujimoto, Miura, Watanabe, Kasai, & Hashimoto. (2020). Brain morphological and functional features in cognitive subgroups of schizophrenia. *Psychiatry Clin Neurosci, 74*(3), 191-203. doi: 10.1111/pcn.12963
